# Ataxic speech disorders and Parkinson’s disease diagnostics via stochastic embedding of empirical mode decomposition

**DOI:** 10.1371/journal.pone.0284667

**Published:** 2023-04-26

**Authors:** Marta Campi, Gareth W. Peters, Dorota Toczydlowska

**Affiliations:** 1 CERIAH, Institut de L’Audition, Institut Pasteur, Paris, France; 2 Department of Statistics & Applied Probability, University of California, Santa Barbara (UCSB), Santa Barbara, California, United States of America; 3 School of Mathematics and Physical Science, University of Technology Sydney, Sydney, Australia; Vinnytsia National Technical University, UKRAINE

## Abstract

Medical diagnostic methods that utilise modalities of patient symptoms such as speech are increasingly being used for initial diagnostic purposes and monitoring disease state progression. Speech disorders are particularly prevalent in neurological degenerative diseases such as Parkinson’s disease, the focus of the study undertaken in this work. We will demonstrate state-of-the-art statistical time-series methods that combine elements of statistical time series modelling and signal processing with modern machine learning methods based on Gaussian process models to develop methods to accurately detect a core symptom of speech disorder in individuals who have Parkinson’s disease. We will show that the proposed methods out-perform standard best practices of speech diagnostics in detecting ataxic speech disorders, and we will focus the study, particularly on a detailed analysis of a well regarded Parkinson’s data speech study publicly available making all our results reproducible. The methodology developed is based on a specialised technique not widely adopted in medical statistics that found great success in other domains such as signal processing, seismology, speech analysis and ecology. In this work, we will present this method from a statistical perspective and generalise it to a stochastic model, which will be used to design a test for speech disorders when applied to speech time series signals. As such, this work is making contributions both of a practical and statistical methodological nature.

## 1 Introduction

Numerous degenerative neurological diseases require continuous monitoring of the patient’s status to ensure treatment regimes are up to date. Furthermore, the same symptoms manifest in multiple of these conditions [1], demanding expensive equipment and advanced expertise for the correct diagnosis. As a solution, the developments of artificial intelligence in biotechnology have started to support these medical settings with automated computational tools that can increasingly identify disorders’ abnormalities in real-life-sensing environments [2–5]. The challenge in detecting symptoms of such nervous system disorders through a computerised practice is accomplished via several modalities (such as speech, handwriting, radiology, gait, etc.) which are employed to reveal indicators of discriminant symptoms associated with neurodegenerative disorders, see [3, 4]. The idea is to map different modality-derived features to the various symptoms and obtain discriminant information about the studied illness. In such a way, what is usually referred to as a “biomarker” could be defined.

This work focuses on Parkinson’s disease, the degenerative disorder of the central nervous system resulting from the death of dopamine-containing cells in the substantia nigra, a midbrain region [1]. It includes both motor and non-motor signs, worsening with disease progression [6, 7]. Medical treatments can alleviate the course of the disease, but no definite cure exists, and an early diagnosis and remote monitoring are critical for prolonging quality of life in those diagnosed, see [8, 9]. The modality in focus in this work is speech which sets our goal as characterising speech anomalies of such a disorder for implementing a pre-screening diagnostic tool and promoting remote telemedicine practices for understanding disease progression. Thus, our interest is restricted to voice symptoms that manifest from this neurodegenerative disorder, part of the speech-motor disease (SMD) class and markers of what is known as *dysarthria*.

Dysarthria refers to a group of divergent SMDs often secondary to neurologic injury (but not limited to it) and exhibits highly variable speech patterns within and across individuals [10]. One of the most established clinical taxonomy for SMD corresponds to the Darley, Aronson, and Brown (DAB) model [11] that foresees 38 atypical speech features rated on a 7-point scale and groups dysarthria types based on speech feature profiles [10]. The DAB model split SMD into two classes, apraxia and dysarthria, and dysarthria into five clusters, flaccid, spastic, ataxic, hypokinetic, and hyperkinetic. Patients often show a combination of the five subtypes (i.e., mixed dysarthria) independently of the final diagnosis, and no speech feature (or a set) has yet to be found discriminative of the different types [1, 12–14]. Furthermore, this clinical system relies entirely on subjective auditory-perceptual observations requiring advanced expert clinical training [10, 13]. Automatic Speaker Recognition (ASR) represent the ideal tool for automatically detecting and monitoring the range of diversity in dysarthria symptoms.

Different types of ASR systems could be used [15, 16]. For example, there are ASR speaker-independent (SI) systems, trained on large multispeaker datasets, or ASR speaker-dependent (SD) systems, trained by an existing SI model to a target speaker or by a unique target speaker’s speech data [10, 17]. Commercially developed SI have low error rates for healthy speakers but appear to perform considerably worse with speech impairments tasks [10, 18]. Thus, extensive work has been conducted on SD systems for speech impairments showing stronger performances than SI [10, 19, 20]. The speech task used for the discrimination might vary and be dependent on the speech methodology or the final goal. These are repeating syllables, spontaneous dialogue, improvised description of a figure, etc. [2]. An ASR system can use several speech features descriptive of the different phases of speech production process, extensively reviewed by [2, 4, 21, 22]. Amongst many, acoustic or vocal tract features describing the articulatory phase are the ones that correlate the most with neurodegenerative disorders. Under the source-filter model [23], a speech signal results from the glottal airflow shaped by the vocal tract filter as it passes through it. Numerous studies in ASR prove that vocal folds features are not as discriminatory as vocal tract features [24]. In particular, representations containing information about the vocal tract’s resonance properties, also known as *formants*. An individual’s speech formant structures are analogous to that individual’s speech fingerprint, thereby characterising unique traits of the filter model specific to a human [17]. Following the introduced evidence, an ASR-SD system, relying on acoustic features and describing the speech formant structure, would represent a powerful solution for characterising different symptoms of dysarthria.

**Fig 1:**
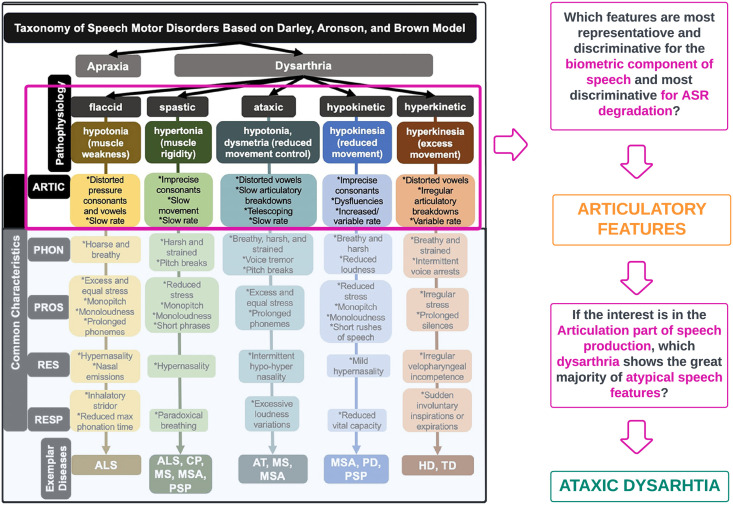
Figure describing the taxonomy of SMD according to the Darley, Aronson, and Brown model. Note that the taxonomy panel was produced by [10] and modified in this paper. Acoustic features representing the vocal tract and capturing formant structure are amongst the most discriminant in ASR tasks. Our interest is to detect the presence or absence of Parkinson’s through such acoustic features. Hence, since one of the early symptoms of Parkinson’s is ataxic speech, which implies several speech abnormalities in the vocal tract, this will be the set of anomalies we aim to discriminate. Furthermore, based on [17], our goal is to construct an ASR-SD system able to deal with complex settings such as non-stationarity of the speech, small sample sizes, unbalanced data, and interpretation of the obtained results concerning gender voices, carrying different formant structure.

**Fig 2:**
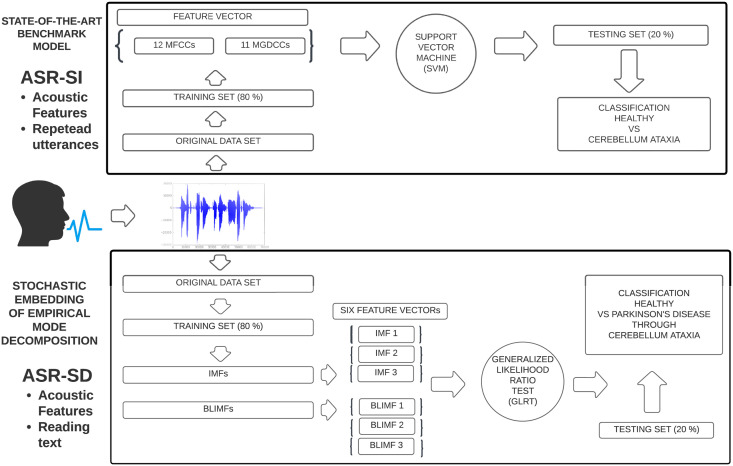
Figure showing the ASR systems detecting ataxic speech. The top panel represents the ASR-SI system implemented by [29], which has been exploited to develop our technique. After having collected the speech data and split it into training and testing sets, the authors extracted (amongst others) Mel Frequency Cepstral Coefficients (MFCCs) and phase-based cepstral coefficients (MGDCCs) and combined them into a unique feature vector to then perform a classification task with a Support Vector Machine (SVM) for the diagnosis of cerebellar ataxia. The bottom panel of the plot shows the steps of our ASR system, which instead is SD and relies on read text as the speech task performed by the participants. The considered data set is given at [38], with people affected by Parkinson’s disease. We constructed the training and testing set and then extracted (amongst others) six different feature vectors, which we have been tested individually through a Generalized Likelihood Ratio Test (GLRT). The classification task targets the detection of ataxic speech with an equivalent statistical framework for diagnosing Parkinson’s disease. Note that an extension of the bottom panel including all the novel features will be presented in Fig 3.

Our work is built upon the following considerations. Firstly, we consider the speech taxonomy provided by the DAB model shown in Fig 1 (produced by [10]). Secondly, we consider Parkinson’s disease and aim to discriminate the presence or absence of such disorder by quantifying ataxic dysarthria or *ataxic speech*. Fig 1 shows that articulatory speech abnormalities are prevalent in this kind of dysarthria and correspond to distorted vowels, slow articulatory breakdowns, telescoping, and slow rate [25–27]. Such abnormalities must be detected through time-varying features of formant structures. Thus, we will consider data for which the assigned speech task is “reading text” to observe the evolution of speech over time rather than using repeated syllables. Ataxic speech is chosen as the discriminant factor for Parkinson’s disease since, beyond being characterised by several abnormalities of the articulatory tract, whose features best capture biometric properties of a human voice, several studies reported a 70–90% of its prevalence once Parkinson’s appears [28]). Moreover, ataxic speech might be one of the earliest indicators of Parkinson’s [6]. Hence, we aim to construct a biomarker that efficiently detects formant structures of ataxic speech abnormalities based on acoustic features formulated through a sophisticated time-series signal processing technique. Fig 1 shows the steps of this procedure. This idea is based on the work proposed in [29], which sought to detect the presence of ataxic speech in participants with cerebellar ataxia using standard acoustic features. By presenting an ad hoc ASR-SD system substituting the one of [29] and efficiently targeting the formant structure of Parkinson’s subjects, we can characterise such a condition through ataxic speech anomalies. Our method is directly comparable to the one proposed by [29] and hence interpretable. Fig 2 shows the two ASR systems and their differences. The top diagram represents the ASR-SI system implemented by [29], while the bottom panel represents the one proposed in this work. Features and classification information will be provided in the text below since the methodologies must be introduced first. Note that, only the novel features are represented in the plot.

**Fig 3:**
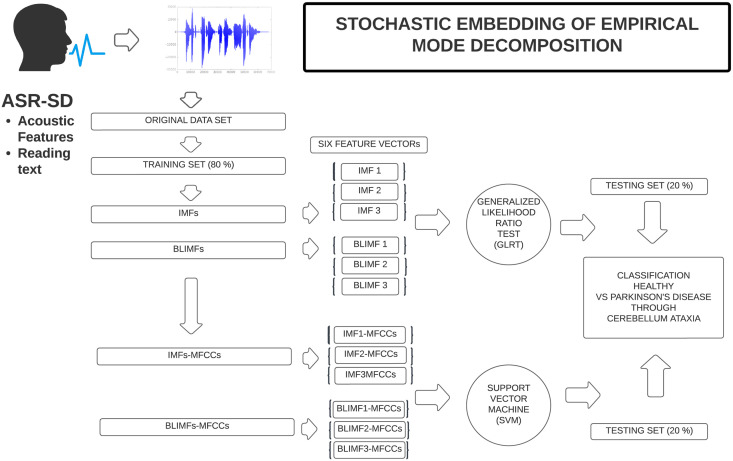
Figure showing the proposed ASR system detecting ataxic speech. It corresponds to an extension of Fig 2 and presenting all the novel features used, hence, the IMFs and the BLIMFs (output of SM2 and SM3) and, further, MFCCs will be extracted on these and an SVM equivalent the one performed by [29] will be carried. Note that only the first 3 bases are retained. Reasons behind this choice will be later introduced.

The research question we want to address is whether it is possible to quantify ataxic speech, as done in [29], more robustly and if, by considering that there will be further statistical confounders, i.e. other types of dysarthria, such ataxic quantification will be discriminative for Parkinson’s disease. In doing so, the following components must be taken into account. Firstly, the developed method should be robust to small sample sizes, often affecting medical diagnostic studies. Secondly, if the data is unbalanced, the designed training and testing procedure combined with the classification method must handle such an issue to avoid introducing undesired bias. The standard practice followed by ASR methodologies is to refine standard glottal/voice features for the classification task or search for a more complex classifier based on deep learning techniques ([30–36]). The third point is that [17] the ASR speech method should account for gender since male and female voices enclose distinct resonant frequencies of the vocal cords and a joint classification would reduce accuracy of the classifier. Furthermore, the classifier must guarantee a physical interpretation of the obtained results, i.e. features better performing should reflect the discriminatory power carried by female or male voices. The other relevant aspect is that considering an ASR-SD is more powerful nowadays in medical settings since averaging results often employed in standard ASR or Speaker Verification tasks might still be too general for such medical biomarker discovery settings, given the lack of substantial reference data sets for specific diseases. Further, before moving to a generalisation protocol, experts providing the final diagnosis and treatments would need highly tested models already studied on several data.

Furthermore, since accuracy levels of at least 80% are required in health diagnostics, such challenges just discussed will require the development of tailored solutions involving sophisticated speech analysis methodologies that should be interpretable in order for them to be relevant for medical practitioners to interpret and trust. This paper aims to address these challenges by providing a novel method for a modelling methodology for ataxic speech symptom detection associated with Parkinson’s disease by addressing two core components of statistical speech analysis for medical diagnosis. The first involves detecting and quantifying ataxic speech anomalies in the case of Parkinson’s disease, with the case study considering speech recordings of patients at various stages of this disease. Secondly, it makes statistical contributions related to developing non-linear and non-stationary time-series methods based on Empirical Mode Decomposition (EMD) [37], where a novel stochastic model representation is established for the EMD which then allows a statistical treatment of EMD to be considered. This is important to undertake statistical analysis tasks such as estimation and inference and to accurately incorporate statistical uncertainty quantification in out-of-sample predictions and forecasts, distinct from model from naive extrapolation, often used in the absence of a stochastic model for EMD. We will show that the implemented methodology outperforms traditional speech methodologies with accuracy scores greater than 80% on the data set collected and provided by King’s College given at [38], available at https://zenodo.org/record/2867216#.ZAiHuRWZO3B.

### 1.1 Introduction to time series empirical mode decomposition

Speech data represents a complex data type that can be analysed through advanced time series decomposition methods since, if appropriately designed, the extracted bases reveal hidden insights into the data generating process, often not visible via the analysis of the original signal. We focus on the time-frequency method [39, 40] known as the EMD. Compared to traditional Fourier-like methods, the EMD is not prescriptive of the functional form of the basis used (as cosine for Fourier, for example) and only specifies the properties its basis functions must satisfy. Further, the EMD can relax requirements for statistical assumptions such as linearity or stationarity. Despite these critical practical features, there has been no statistical formalisation of a stochastic representation or embedding of the empirical algorithm that the EMD offers, and we address this challenge in this manuscript.

The EMD basis functions, known as Intrinsic Mode Functions (IMFs), carry the advantage of being monocomponent [41]. A monocomponent signal is described in the time-frequency (t,f)-domain by one single “ridge” corresponding to an elongated region of energy concentration. In addition, considering the crest of the ridge as a graph of Instantaneous Frequency (IF) vs time, one requires the IF of a monocomponent signal to be a scalar-valued function of time. In such a way, one is allowed to form the analytic extensions of each of the basis function IMFs via a well-defined Huang-Hilbert transform to characterise the collection of frequency representations obtained explicitly, i.e. the IFs of the signal, in our case the speech signals, see discussion in [39, 42–44]. The EMD method then utilises the fact that a multicomponent signal may be described as the sum of two or more monocomponent signals. A basis decomposition method utilising such characterising features can capture both time and frequency events in a localised fashion, which is extremely useful when there are non-stationarity effects present, as in speech.

Developing a stochastic representation or embedding along with a family of statistical model representations for the EMD method to complement its algorithmic formulation will be achieved by considering three methodological problem statements (PS1, PS2, PS3) addressed in this paper. The first problem is establishing a path-wise statistical model for the IMFs, satisfying the definitions provided in [37] that will also be consistent with the developed stochastic representation. The second problem statement considers the assumption that the EMD is algorithmically applied to the realisation of a time series signal sampled from an unknown stochastic process. Given the realised time series, the IMFs, per path, are then considered deterministic unknown functions that must be estimated from the samples. Therefore, in PS2, we seek to determine a stochastic version of the IMF decomposition compatible at a population process level with the pathwise representation of the deterministic decomposition being estimated under the solution to PS1.

Given that we will work with spline model representations as the solution to PS1, it becomes natural to consider whether Gaussian Processes (GP) [45] stochastic model embeddings will satisfy the solution to PS2 when stochastically embedding the IMFs. In this work’s context, a Gaussian process will be considered a continuous-time stochastic process for which all finite-dimensional distributions follow multivariate normal distributions. One may then interpret the GP as a random variable on *L*^2^([0, 1]) such that the individual sample paths mapping [0,1]→R are considered random functions. In particular, there is a known connection between such functions when they are represented by splines, which under appropriate conditions are known to be suitable sample path realisations for GPs, see [46]. The challenge will be to ascertain whether this class of GP stochastic models will sufficiently satisfy the requirements imposed on the characteristic properties that such a representation should capture if it is to represent an EMD decomposition as a stochastic representation adequately.

Furthermore, GPs are a robust inference supervised machine learning technique used in many applications, given that they can be entirely specified by their mean and covariance, or kernel, functions. This will allow the definition of a stochastic representation with practical utility in performing tasks such as estimation, inference and forecasting. We will demonstrate that the GP stochastic representation we will develop for EMD basis functions IMFs when aggregated together to represent the original signal, can be considered as a special class of multi-kernel (MKL) GP (see review in [47]) stochastic model representation of the original time series signal. In practice, the EMD is then learning the multi-kernel spectral decomposition in terms of the number of kernel components to consider and their characteristic time-frequency structure for each kernel component. MKL representations can be achieved through multiple strategies developed in the literature ([48–51]).

The third problem addressed (PS3) pertains to the suitable selection of the covariance function used to capture the IMFs being stochastically modelled by GPs adequately. Since IMFs correspond to a collection of non-stationary basis functions, there is a requirement to properly design the family of kernel functions to accurately model the IMF spline representations estimated under the EMD basis extraction procedure, known as sifting. In this regard, in non-trivial applications such as speech analysis focused on in this manuscript, standard parametric kernels such as the Matern kernel and the RBF kernel (see [45]) will not suffice. Instead, we will develop two classes of solutions to this problem that generate two different families of stochastic model GP representations of EMD decompositions. The first is based on a family of data-adaptive kernels known as the Fisher kernel [52–55], which provides a generic mechanism incorporating generative probability models into the development of the covariance operator that will be data-adaptive and act as a flexible time series kernel. The second approach is based on a novel framework to learn optimal partitions of the time-frequency plane that utilises the IFs obtained from the EMD basis IMFs to partition the energy spectrum into localised regions that can then be modelled via localised GPs. One of the challenges with this second approach is how best to learn the time-frequency partition rule. This is solved via a novel application of Cross Entropy optimisation (CEM), which is a stochastic optimisation technique that Rubinstein first presented in 1999 (see [56, 57]). Once the optimal core bandwidths are computed, a new set of frequency band-limited bases we term “band-limited” IMFs (BLIMFs) will be derived. These new set of basis functions are obtained by aggregating the original IMFs sample points according to the location of their IFs within the regions of the computed optimal bandwidths partition. With such a partition model, we can characterise adaptive local bandwidths of the IMFs frequency domain with a kernel function in a GP setting.

### 1.2 Contributions, notation and structure

There are multiple contributions made by this work both in the direction of medical diagnosis for ataxic speech in Parkinson’s and for signal processing decomposition methods in speech analysis. These are given as follows.

- A stochastic embedding model is developed for the EMD method that is consistent with the properties of the IMFs. The stochastic model for the IMFs is compatible with statistical representation comprised of B-spline and P-spline and proposes flexible statistical models that readily lend themselves to estimation, inference and statistical forecasting methods for EMD decompositions. Yet, this needed to be improved in the time-series signal processing literature, since traditionally the EMD method did not admit a probabilistic model representation, so we have developed one in this work.
- The following notation will be used throughout: *t*_0_ < *t*_1_ < … < *t*_*N*_ denotes signal observation times; the time series signal is denoted by s(t):T→R and is observed at ${\left\{s(t_{i})\right\}}_{i=1}^{N}$; the continuous time spline reconstruction of the signal is denoted by $\tilde{s}\left(t\right):T\to R$; the *L* IMF basis function from the EMD method are denoted by ${\left\{\gamma_{l}\left(t\right)\right\}}_{l=1}^{L}$ such that each satisfies $\gamma_{l}\left(t\right):T\to R$; *L* generically denoted the total number of IMFs extracted for a given signal; the analytic extension of the *l*-th IMF will be denoted by ${\overset{ˇ}{\gamma }}_{l}\left(t\right)=H[\gamma_{l}\left(t\right)]$ where H[·] denotes the Hilbert transform which produces the analytic signal $z_{l}\left(t\right)=\gamma_{l}\left(t\right)+i{\overset{ˇ}{\gamma }}_{l}\left(t\right)$; F[·] will denoted the Fourier transform; when extracting IMF basis functions under the EMD method sifting algorithm, we will denote by ${\tilde{s}}^{U_{l}}\left(t\right)$ the upper envelope used in sifting that is a spline interpolating the maximum of the current best estimate of the *l*-th IMF and analogously by ${\tilde{s}}^{B_{l}}\left(t\right)$ the lower envelope of the *l*-th IMF interpolating the minimum of the current best estimate of the *l*-th IMF in the iterative IMF extraction algorithm known as sifting; finally, we will denote the collection of frequency band limited IMFs by ${\left\{\gamma_{m}^{\left(BL\right)}\left(t\right)\right\}}_{m=1}^{M}$ the band-limited IMF construction based on *M* total specified bandwidths.;
  The paper is organised as follows: firstly, a review of the EMD method is shown. We refer to [17] as main reference. Secondly, the EMD stochastic embedding set up is proposed with a set of objectives that must be satisfied. Afterwards, the stochastic embedding is formally developed, with the required notions presented to achieve it. Note that, three different system models will be formulated in this section: one for the stochastic embedding of the original signals and two which are the ones relating to the EMD and proposed in this manuscript. Section 5 presents how to develop a generative embedding kernel based on the Fisher kernel. Furthermore, the formulation of the cross-entropy problem with the derived solution used to formalise an optimal time-frequency partition for the second stochastic embedding is presented. Section 6 introduces the framework of speech based medical diagnostic with a subsection on motivation for Parkinson’s speech detection, a subsection standard benchmark model solving this task and the GLRT Test used to test the presence or absence of Parkinson’s disease developed in this paper. The last section shows the experiments results and discussion conducted on the speech data for Parkinson’s detection.

## 2 Statistical model framework for empirical mode decomposition

This section introduces a formalism required to understand the EMD method and builds upon the work presented in [17]. EMD basis characteristics of IMFs have been defined in [37] through a set of non-constructive properties only and are obtained via a procedure known as sifting, based on a recursive extraction of the signal energy associated with the intrinsic time scales of the original signal. They are therefore ordered according to their number of oscillations or convexity changes, and they furthermore satisfy the property that their sum reproduces the original realised signal path. Hence, the observed time series is reconstructed in principle exactly when the resulting IMFs are estimated or extracted numerically in a manner that perfectly satisfies the characterising properties of the EMD method.

Consider a continuous non-stationary speech signal *s*(*t*) observed as a sample recording at times 0 = *t*_1_ < … < *t*_*N*_ = *T*. When applying the EMD basis decomposition framework, we first convert the partially observed discrete time signal *s*(*t*) into a continuous time analog signal, denote by $\tilde{s}\left(t\right)$. To achieve this we use a natural cubic polynomial spline. We will also express the EMD bases ${\left\{\gamma_{l}\left(t\right)\right\}}_{l=1}^{L}$ as natural cubic splines, derived from representation $\tilde{s}\left(t\right)$.

**Definition 2.1**. *Given a set of l knots a* = *τ*_1_ < *τ*_2_ < … < *τ*_*l*_ = *b*, *a function* $\tilde{s}:[a,b]\to R$ *is called a cubic polynomial spline if*:

-
$\tilde{s}\left(\cdot \right)$ is a polynomial of degree 3 on each interval (*τ*_*j*_, *τ*_*j*+1_) (*j* = 1, …, *l* − 1)
-
$\tilde{s}\left(\cdot \right)$ is twice continuously differentiable

*It is then a natural cubic spline when* ${\tilde{s}}^{\prime \prime }\left(a\right)={\tilde{s}}^{\prime \prime }\left(b\right)=0$ .

Hence, the speech signal representation $\tilde{s}\left(t\right)$ is expressed in the class of truncated power basis, where the knot points are placed at the sampling times (*τ*_*i*_ = *t*_*i*_)

$$\begin{array}{c} \tilde{s}\left(t\right)=a_{0}+a_{1}t+a_{2}t^{2}+a_{3}{\left(t-\tau_{1}\right)}_{+}^{3}+\ldots +a_{3+l-2}{\left(t-\tau_{l-1}\right)}_{+}^{3}. \end{array}$$

The coefficients are estimated by standard penalised least squares

$$\begin{array}{c} \sum_{i=1}^{N-1}{\left(s\left(t_{i}\right)-\tilde{s}\left(t_{i}\right)\right)}^{2}+\lambda \int_{t_{i}}^{t_{i+1}}{\tilde{s}}^{\prime \prime }{\left(t\right)}^{2}dt \end{array}$$

with natural cubic spline constraints ${\tilde{s}}^{\prime \prime }\left(0\right)={\tilde{s}}^{\prime \prime }\left(t_{N}\right)=0$ and where λ > 0 controls smoothness of the representation. In this case, the number of total convexity changes (oscillations) of the analog signal $\tilde{s}\left(t\right)$ within the time domain [0, *t*_*N*_] is denoted by to L∈N. One may now define the EMD decomposition of a speech signal $\tilde{s}\left(t\right)$ as follows.

**Definition 2.2 (Empirical Mode Decomposition)**. *The Empirical Mode Decomposition of signal* $\tilde{s}\left(t\right)$ *is represented by the finite number of non-stationary basis functions known as Intrinsic Mode Functions (IMFs), denoted by* {*γ*_*l*_(*t*)}, *such that*

$$\begin{array}{c} \tilde{s}\left(t\right)=\sum_{l=1}^{L}\gamma_{l}(t)+r(t) \end{array}$$ (1)

*where r*(*t*) *represents the final residual (or final tendency) extracted, which has only a single convexity. In general the γ*_*l*_ *basis will have l-convexity changes throughout the domain* (*t*_1_, *t*_*N*_) *and each IMF satisfies*:

- ***Oscillation** The number of extrema and zero-crossing must either equal or differ at most by one;*
$$\begin{array}{c} abs(|\{\frac{d\gamma_{l}\left(t\right)}{dt}=0:\;t\in (t_{1},t_{N})\}|-|\{\gamma_{l}\left(t\right)=0:\;t\in (t_{1},t_{N})\}|)\in \{0,1\} \end{array}$$ (2)
- ***Local Symmetry** The local mean value of the envelope defined by a spline through the local maxima denoted* ${\tilde{s}}^{U_{l}}\left(t\right)$ *and the envelope defined by a spline through the local minima denoted by* ${\tilde{s}}^{B_{l}}\left(t\right)$ *is equal to zero pointwise i.e.*
$$\begin{array}{c} m_{l}\left(t\right)=(\frac{{\tilde{s}}^{U_{l}}\left(t\right)+{\tilde{s}}^{B_{l}}\left(t\right)}{2})I(t\in [t_{1},t_{N}])=0 \end{array}$$ (3)
  *The minimum requirements of the upper and lower envelopes are:*
$$\begin{array}{c} \begin{array}{cc} {\tilde{s}}^{U_{l}}\left(t\right) & =\gamma_{l}\left(t\right),\;\;if\;\;\frac{d\gamma_{l}\left(t\right)}{dt}=0\;\&\;\frac{d^{2}\gamma_{l}\left(t\right)}{dt^{2}}<0,\\ {\tilde{s}}^{U_{l}}\left(t\right) & \geq \gamma_{l}\left(t\right)\;\forall t\in \left(t_{1},t_{N}\right)\\ {\tilde{s}}^{B_{l}}\left(t\right) & =\gamma_{l}\left(t\right),\;\;if\;\;\frac{d\gamma_{l}\left(t\right)}{dt}=0\;\&\;\frac{d^{2}\gamma_{l}\left(t\right)}{dt^{2}}>0,\\ {\tilde{s}}^{B_{l}}\left(t\right) & \leq \gamma_{l}\left(t\right)\;\forall t\in \left(t_{1},t_{N}\right). \end{array} \end{array}$$ (4)

This definition provides characteristic properties that an IMF basis, *γ*_*l*_(*t*), under the EMD method should satisfy. Evidently, it is not constructive, i.e. prescriptive of the functional form of the basis. Therefore, in this manuscript, we opt to utilise throughout the same flexible natural cubic spline representation as used to represent the speech signal interpolation $\tilde{s}\left(t\right)$ also for the IMFs. Such a B-spline based representation for the realised deterministic basis decomposition that makes up the statistical model for the EMD pathwise representation will be essential to motivate the use of the Gaussian process stochastic model embedding for the stochastic process based representation we develop for the EMD method.

One can note that each IMF carries a unique number of convexity changes that can occur at any time spacings. Typically, the times of convexity change are irregularly spaced and reflect non-stationarity in a local bandwidth of the frequencies that characterize the signal at that time instant. As a result of this property, one can still order the basis IMF’s naturally according to the unique number of total convexity changes they produce in (*t*_1_, *t*_*N*_).

As outlined in [37], the construction of an IMF basis is directly linked to the concept of local symmetry required to handle non-stationary data. This notion is enclosed by the mean envelope that captures a local time scale, and the definition of a local averaging time scale is hence bypassed. Such a requirement is fundamental to avoid asymmetric waves affecting the concept of instantaneous frequency, formalised below.

### 2.1 Extraction of EMD basis functions Intrinsic Mode Functions (IMFs): The sifting procedure

We briefly outline the process applied to extract recursively the IMF basis representations, which is a procedure known as *sifting*, see [58]. To extract the *l*-th IMF The first step consists of computing extrema of the current signal representation after having removed the previously extracted IMFs by ${\tilde{s}}_{l}\left(t\right):=\tilde{s}\left(t\right)-\sum_{i=1}^{l-1}\gamma_{i}\left(t\right)$, which still admits a spline representation. Using the spline representation of ${\tilde{s}}_{l}\left(t\right)$ one needs to find the roots of the first derivative ${\tilde{s}}_{l}^{\prime }\left(t\right)$ to produce the sequence of time points for successive maxima and minima given by:

$$\begin{array}{c} {\left\{t_{j}^{*}\right\}}_{l=1}^{L}=\{t\in \left[t_{1},t_{N}\right]\;:\;a_{1}+2a_{2}t+3\sum_{i=3}^{3+l-2}a_{i}{\left(t-\tau_{1}\right)}_{+}^{2}=0\}. \end{array}$$

Without loss of generality, we assume the maxima occur at odd intervals, i.e. $t_{2j+1}^{*}$, and minima occur at even intervals, i.e. $t_{2j}^{*}$. The second step of sifting builds an upper (${\tilde{s}}^{U_{l}}\left(t\right)$) and lower (${\tilde{s}}^{B_{l}}\left(t\right)$) envelope of ${\tilde{s}}_{l}\left(t\right)$ using two natural cubic splines through the sequence of maxima and the sequence of minima respectively:

$$\begin{array}{c} \begin{array}{cc} {\tilde{s}}^{U_{l}}\left(t\right) & =a_{0}^{U_{l}}+a_{1}^{U_{l}}t+a_{2}^{U_{l}}t^{2}+\sum_{i=0}^{\left\lfloor L/2\right\rfloor }a_{i+3}^{U_{l}}{\left(t-t_{2i+1}^{*}\right)}_{+}^{3},\\ {\tilde{s}}^{B_{l}}\left(t\right) & =a_{0}^{L_{k}}+a_{1}^{B_{l}}t+a_{2}^{B_{l}}t^{2}+\sum_{i=0}^{\left\lfloor L/2\right\rfloor }a_{i+3}^{B_{l}}{\left(t-t_{2i}^{*}\right)}_{+}^{3}, \end{array} \end{array}$$

such that ${\tilde{s}}^{U_{l}}\left(t\right)\geq {\tilde{s}}_{l}\left(t\right)$ ∀*t* with ${\tilde{s}}^{U_{l}}\left(t_{2j+1}^{*}\right)={\tilde{s}}_{l}\left(t_{2j+1}^{*}\right)$ for all odd $t_{j}^{*}$ and strictly greater otherwise; and equivalently ${\tilde{s}}^{B_{l}}\left(t\right)\leq {\tilde{s}}_{l}\left(t\right)$ ∀*t* with ${\tilde{s}}^{B_{l}}\left(t_{2j}^{*}\right)={\tilde{s}}_{l}\left(t_{2j}^{*}\right)$ for all even $t_{j}^{*}$ and strictly less than otherwise. One then utilises these envelopes to construct the mean signal denoted by *m*_*l*_(*t*) given in Eq (3), which will then be used to compensate the current representation of the speech signal by ${\tilde{s}}_{l}\left(t\right)={\tilde{s}}_{l}\left(t\right)-m_{l}\left(t\right)$ at each time point *t* ∈ [*t*_1_, *t*_*N*_]. This procedure is then repeated on the compensated signal, where again the current maxima and minima are obtained to produce envelopes which in turn produce a new estimate of the mean *m*_*l*_(*t*) which in turn is used in a defluctuation step to compensate the signal ${\tilde{s}}_{l}\left(t\right)$. This is repeated until the conditions specified in Definition 2.2 for the envelope and mean functions are satisfied, which when achieved produce the current deflucuated version of the signal ${\tilde{s}}_{l}\left(t\right)$ as the *l*-th IMF *γ*_*l*_(*t*). This procedure then repeats again for the *l* + 1-th IMF extraction working now on signal ${\tilde{s}}_{l+1}\left(t\right):=\tilde{s}\left(t\right)-\sum_{i=1}^{l}\gamma_{i}\left(t\right)$, and the entire sifting process terminates when the *L* + 1-st IMF is extracted and it corresponds to the IMF ‘tendency’ which only has one convexity change in [*t*_1_, *t*_*N*_] and is often denoted distinctly by *r*(*t*), see [17] for an algorithm and further details.

### 2.2 Obtaining Instantaneous Frequencys (IFs) from IMF basis functions

The EMD method extracts a set of basis functions (IMFs), each of which will admit a time-varying frequency structure that can be characterized by their corresponding instantaneous frequeny (IF) signal. The IF of a given IMF basis is extracted in the following stages.

First, one takes the Hilbert Transform of each IMF ${\left\{\gamma_{l}\left(t\right)\right\}}_{l=1}^{L}$, in order to construct a set of analytic extensions ${\left\{{\overset{ˇ}{\gamma }}_{l}\left(t\right)\right\}}_{l=1}^{L}$ via the Hilbert transform as follows:

$$\begin{array}{c} {\overset{ˇ}{\gamma }}_{l}\left(t\right)=H[\gamma_{l}\left(t\right)]=\frac{1}{\pi }\lim_{\epsilon \to \infty }\int_{-\epsilon }^{+\epsilon }\frac{\gamma_{l}\left(\tau \right)}{t-\tau }d\tau \end{array}$$

which then produces the collection of analytic signals {*z*_*l*_(*t*)} with $z_{l}\left(t\right)=\gamma_{l}\left(t\right)+{\overset{ˇ}{\gamma }}_{l}\left(t\right)$. We observe that when *γ*_*l*_(*t*) is a proper IMF such that it respects the restrictions defined in (4), its Hilbert transform can be obtained in closed form. The complex analytical signal *z*_*l*_(*t*) can be then represented by the polar representation $z_{l}=a_{l}\left(t\right)e^{ȷ\theta_{l}\left(t\right)}$ with time varying amplitude $a_{l}\left(t\right)=\sqrt{\gamma_{l}^{2}\left(t\right)+{\overset{ˇ}{\gamma }}_{l}^{2}\left(t\right)}$ and time varying phase $\theta_{l}\left(t\right)=\text{arctan}\frac{{\tilde{\gamma }}_{l}\left(t\right)}{\gamma_{l}\left(t\right)}$.

The instantaneous frequency *ω*_*l*_(*t*) for IMF *γ*_*l*_(*t*) is then found from the time-varying phased of *z*_*l*_(*t*) as the rate of change given by:

$$\begin{array}{c} \omega_{l}\left(t\right)=\frac{1}{2\pi }\frac{d\theta_{l}\left(t\right)}{dt}=\frac{1}{2\pi }\frac{{\overset{ˇ}{\gamma }}_{l}^{\prime }\left(t\right)\gamma_{l}\left(t\right)-{\overset{ˇ}{\gamma }}_{l}\left(t\right)\gamma_{l}^{\prime }\left(t\right)}{\gamma_{l}^{2}\left(t\right)+{\overset{ˇ}{\gamma }}_{l}^{2}\left(t\right)}. \end{array}$$

As observed in [37] conditions (4) that characterize the IMF properties are specified to ensure that the instantaneous frequency remains positive and therefore admits a meaningful physical interpretation.

Since, we adopt a statistical model representation for the IMFs based on cubic splines one can utilise this representation of the *l*-th IMF to obtain the Hilbert transform of the sum of local cubic polynomial transforms, see for details [59]:

$$\begin{array}{c} {\overset{ˇ}{\gamma }}_{l}\left(t\right)=H[\gamma_{l}\left(\tau \right)]=\frac{1}{\pi }\sum_{i=1}^{N-1}{\overset{ˇ}{\gamma }}_{l_{i}}\left(t\right)\;\tau_{i-1}<t\leq \tau_{i} \end{array}$$

where △_*i*_ = *τ*_*i*_ − *τ*_*i*−1_ and ${\overset{ˇ}{\gamma }}_{l_{i}}\left(t\right)$ is the Hilbert transform of the i-th polynomial:

$$\begin{array}{c} \begin{array}{cc} {\overset{ˇ}{\gamma }}_{l_{i}}\left(t\right) & =(a_{l_{i}}t^{3}+b_{l_{i}}t^{2}+c_{l_{i}}t+d_{l_{i}})\text{log}(\frac{t}{t-{△}_{i}})\\ & +a_{l_{i}}(\frac{{△}_{i}^{2}t}{2}-{△}_{i}t^{2}-\frac{{△}_{i}^{3}}{3})+b_{l_{i}}(-{△}_{i}t-\frac{{△}_{i}^{2}}{2})-c_{l_{i}}{△}_{i}. \end{array} \end{array}$$

Such a representation for the IMF *γ*_*l*_(*t*) produces a smooth, differentiable, continuous function, it is approximated by the class of polynomial basis in the *L*^2^ space.

## 3 EMD stochastic embedding set-up

We have shown in Section 2 that working with cubic splines for the representation of the EMD method is advantageous from many perspectives. Firstly it is suitable to represent the interpolated signal $\tilde{s}\left(t\right)$ from the observed time series ${\left\{s(t_{i})\right\}}_{i=1}^{N}$ in an optimal fashion based on minimising mean squared error. Secondly, it allows one to perform the sifting procedure readily when representing the envelope functions and results in a collection of IMF basis functions ${\left\{\gamma_{l}\right\}}_{l=1}^{L}$ representations that are also cubic splines. Thirdly, the analytic extension via the Huang Hilbert transform, used to obtain the instantaneous frequency, admits closed form solutions for the representations of the IFs ${\left\{\omega_{l}\right\}}_{l=1}^{L}$ which is also characterised readily by cubic splines. Lastly, and most importantly, when considering moving from the path-wise EMD method basis extraction for one of the time series realised trajectories to a stochastic process embedding representation, the representation of IMFs via cubic splines allows one to utilise the established connection between Gaussian processes and B-splines to motivate working with Gaussian process stochastic embeddings.

### 3.1 EMD stochastic embedding objectives

In developing the stochastic embedding of the EMD, we will distinguish between the deterministic (realised) or empirical EMD decomposition for a given signal trajectory, satisfying at any time *t* ∈ [0, *T*] the property of EMD decomposition

$$\begin{array}{c} s\left(t\right)=\sum_{l=1}^{L}\gamma_{l}\left(t\right)+r\left(t\right) \end{array}$$

for IMF *γ*_*l*_(*t*) satisfying the mathematical characterisation given in Definition 2.2; and the stochastic process embedding of the EMD representation, denoted at any time *t* ∈ [0, *T*], by the random variables (upper case for random variables)

$$\begin{array}{c} S\left(t\right)\overset{d}{=}\sum_{l=1}^{L}\Gamma_{l}\left(t\right)+R\left(t\right) \end{array}$$

The challenge with developing a stochastic embedding for EMD method is that it will be required to satisfy a few core features:

1. Sample paths of the embedded EMD stochastic process should be able to be consistent with the basis functions for the IMFs obtained from the empirical sample based characteristics that represent the classical EMD method as set-up in Definition 2.2.;
2. Since the EMD method satisfies for each realised sample time-series trajectory $\tilde{s}\left(t\right)$ that
  $$\begin{array}{c} \tilde{s}\left(t\right)=\sum_{l=1}^{L}\gamma_{l}\left(t\right)+r\left(t\right) \end{array}$$
  then one would naturally require such a property to be inherited at the population stochastic process level such that:
  $$\begin{array}{c} \tilde{S}\left(t\right)\overset{d}{=}\sum_{l=1}^{L+1}\Gamma_{l}\left(t\right) \end{array}$$
  where we have denoted the stochastic process for *R*(*t*) by Γ_*L*_(*t*) to reduce notational burden.
  Ideally the representations of processes $\tilde{S}\left(t\right)$ and IMF stochastic processes ${\left\{\Gamma_{l}\left(t\right)\right\}}_{l=1}^{L}$ would satisfy:
  1. Stochastic processes used to model $\tilde{S}\left(t\right)$ and IMF processes ${\left\{\Gamma_{l}\left(t\right)\right\}}_{l=1}^{L+1}$ have known finite dimensional distributions and are from family of known stochastic process models which are easily parameterised and characterised. We will denote this family of models for distributions at time *t*
  2. Stochastic processes used to model $\tilde{S}\left(t\right)$ and IMF processes ${\left\{\Gamma_{l}\left(t\right)\right\}}_{l=1}^{L+1}$ would also ideally be easily calibrated to realised EMD sample based decompositions via standard estimation methods like maximum likelihood estimation with closed form expressions for the likelihood of the model for the stochastic embedding.
  3. IMF stochastic processes ${\left\{\Gamma_{l}\left(t\right)\right\}}_{l=1}^{L}$ are of the same family of stochastic process model as that which represents the signal stochastic process $\tilde{S}\left(t\right)$. In other words if, for each time *t*, one has that random variable $\tilde{S}\left(t\right)∼F\in F$ is distributed by *F* in a family of distribution models F where
    $$\begin{array}{c} \tilde{S}\left(t\right)∼F(a;\Psi_{\tilde{S}}):=\int_{-\infty }^{a}\ldots \int_{-\infty }^{a}f_{\Gamma_{1},\ldots ,\Gamma_{L+1}}(\gamma_{1},\ldots ,\gamma_{L+1})d\gamma_{1}\ldots d\gamma_{L+1} \end{array}$$
    with $\Psi_{\tilde{S}}$ denoting the parameters of the model that indexes the family member from F and furthermore, where $f_{\Gamma_{1},\ldots ,\Gamma_{L+1}}$ is the joint distribution of the IMF random variables and tendency at time *t*, then it also holds that for each *t* ∈ [0, *T*] and *l* ∈ {1, …, *L* + 1} the distribution of the IMF random variables satisfies that it is also a member of this family of distribution models such that
    $$\begin{array}{c} \Gamma_{l}\left(t\right)∼F(s,\Psi_{\Gamma_{l}})\in F, \end{array}$$
    indexed by parameter vectors Ψ_*l*_.
  4. Another desirable property for the stochastic embedding representation of EMD would be to have the conditional distributions also members of the same family of distributions of $\tilde{S}\left(t\right)$, such that for each *t* ∈ [0, *T*] and any combination of *J* ≤ *L* + 1 indexes denoted by subset K⊆{1,…,L+1} one has that the random variable
    $$\begin{array}{c} \sum_{i\in K}\Gamma_{i}\left(t\right)|\Gamma_{1,\ldots ,L\backslash K}∼F(s;\Psi_{K})∼F \end{array}$$
    Note: In the case one assumes an independence model approximation for the joint distribution of the IMF random variables and tendency at each time *t* ∈ [0, *T*] such that
    $$\begin{array}{c} f_{\Gamma_{1},\ldots ,\Gamma_{L},R}(\gamma_{1},\ldots ,\gamma_{L},r)=\prod_{l=1}^{L}f_{\Gamma_{l}}(\gamma_{l})f_{R}(r) \end{array}$$
    Then the EMD method decomposition implies that the stochastic representation of the IMFs are closed under convolution. This means that at each time *t* the random variable for the signal *S*(*t*)∼*F*(*s*;Ψ_*S*_) and the random variables for the IMFs $\Gamma_{i}∼F(s;\Psi_{\Gamma_{i}})$ satisfy that
    $$\begin{array}{c} F(s;\Psi_{S})={⊛}_{i=1}^{L}F(s;\Psi_{\Gamma_{i}})⊛F(s;\Psi_{R}) \end{array}$$
    such that $F(s;\Psi_{S}),F(s;\Psi_{\Gamma_{1}}),\ldots ,F(s;\Psi_{\Gamma_{L}}),F(s;\Psi_{R})\in F$

## 4 Developing a stochastic embedding of EMD

In this section we develop two approaches for the stochastic embedding of the EMD method which will be consistent with the EMD empirical decomposition whilst also concurrently satisfying the properties set out for such a stochastic representation of EMD given in Section 3.1. To achieve this we will develop two different system models each of which will be based on versions of multi-kernel Gaussian Processes models with specially selected kernel structures. The reference baseline or benchmark model we will compare to these two novel system models for EMD stochastic representation will be a Gaussian process fit directly to the original signal *s*(*t*).

Gaussian Processes (GPs) are a highly expressive family of stochastic models widely adopted in machine learning, see [45]. Formally, a Gaussian process is a collection of random variables, any finite number of which have a joint Gaussian distribution, which is entirely described by its mean and kernel covariance function as detailed in Definition 4.1. The positive definite covariance function often referred to as kernel determines the class of functions from which such processes sample paths take support.

**Definition 4.1 (Gaussian Process (GP))**. *Denote by* f(x):X→R *a stochastic process, parametrised with state-space* {x}∈X, *where* $X\subseteq R^{d}$. *The random function f*(*x*) *is a Gaussian Process if all finite dimensional distributions are Gaussian, where for any* n∈N, the random vector (*f*(*x*_1_), *f*(*x*_2_), …, *f*(*x*_*n*_)) *is jointly normally distributed. We can therefore interpret a GP formally defined by the following class of random functions*:

$$\begin{array}{c} f:=\{f\left(\cdot \right):X\to R:f\left(\cdot \right)∼GP(\mu \left(\cdot ,\psi_{f}\right),k\left(\cdot ,\theta_{f}\right))\} \end{array}$$ (5)

with $\mu (\cdot ,\psi_{f}):X\to R$, $k\left(\cdot ,\theta_{f}\right):X\times X\to R^{+}$,

$$\begin{array}{c} \begin{array}{cc} & \mu \left(\cdot ,\psi_{f}\right)=E[f(\cdot )]\\ & k\left(\cdot ,\theta_{f}\right)=E[(f\left(\cdot \right)-\mu \left(\cdot ,\theta_{\mu }\right))(f\left(\cdot \right)-\mu \left(\cdot ,\theta_{\mu }\right))] \end{array} \end{array}$$ (6)

The properties of the functions, i.e. smoothness, periodicity, etc., are determined by the sufficient statistic given by the covariance kernel function.

Before introducing these GP models, we will motivate theoretically why the class of GP models is suitable for a stochastic embedding that will be shown to be both meaningful for regularised spline representations of IMFs as well as suitable to satisfy the properties outlined for such a stochastic embedding of EMD discussed in Section 3.1.

### 4.1 Spline representations of an IMF and reproducing kernel hilbert spaces

In order to make explicit the connection between using spline models to represent the path-wise empirical EMD decomposition of $\tilde{s}\left(t\right)$ and the stochastic embedding via a multi-kernel Gaussian process, we will recall briefly known connections between splines and Gaussian Processes (GPs). Splines may be viewed as limits of interpolations related to stationary Gaussian processes. Hence, we will explore further this connection as follows.

Consider seeking to recover the *l*-th unknown IMF function *γ*_*l*_(*t*) for *t* ∈ [0, *T*] based on current sifting defluctuation step data ${\tilde{s}}_{l}\left(t\right):=\tilde{s}\left(t\right)-\sum_{i=1}^{l-1}\gamma_{i}\left(t\right)$ at time points *t*_1_, …, *t*_*N*_ denoted as observations here generically by $y_{i}:={\tilde{s}}_{l}(t_{i})$. That is one has data $\{t_{i},y_{i}\}\in T\times R$ and we seek the function representation for the *l*-th IMF $\gamma_{l}\left(t\right):T\to R$ that minimizes the objective given generically in Eq (7), for instance which may be the familiar penalised residual sum-of-squares,

$$\begin{array}{c} Q\left(\gamma_{l}\right)=\sum_{i=1}^{N}L(y_{i},\gamma_{l}(t_{i}))+\lambda J(\gamma_{l}) \end{array}$$ (7)

where *L* is a loss function, λ ≥ 0 is regularisation strength and *J* is a functional imposing smoothness on the IMF representation *γ*_*l*_. One can connect the regularised spline solution to GPs by considering Reproducing Kernel Hilbert Spaces (RKHS) to explore the unifying framework to motive the GP stochastic embedding model, see details in [60] and more recent works in [46, 61, 62].

A Hilbert space H is an inner-product space which is complete in the metric induced by its norm. For every Hilbert space of functions on a set T, one may define for each t∈T the evaluation functional *f*: *t* ↦ *f*(*t*). If every evaluation functional in the Hilbert space is bounded, then one obtains a Reproducing Kernel Hilbert Space (RKHS). Note *L*^2^ is not an RKHS since the Dirac-delta function is not in *L*^2^. In an RKHS the Riesz representation theorem states that one may find, for each *t* a representer $k_{t}\in H$ such that

$$\begin{array}{c} f\left(t\right)=\left\langle f,k_{t}\right\rangle . \end{array}$$

Then one can define a function known as the kernel k:T×T→R by *k*(*s*, *t*) = *k*_*s*_(*t*). This function will be unique to a given RKHS H and has the properties of symmetry, nonnegative definiteness and satisfies the reproducing property 〈*k*(⋅, *s*), *k*(⋅, *t*)〉 = *k*(*s*, *t*).

To understand why the RKHS space and reproducing kernel *K* are introduced, consider the space of all finite linear combinations of functions {k(·,s)|s∈T} with the inner product given by 〈*k*_*s*_, *k*_*t*_〉 = *k*(*s*, *t*) along with linearity. It is then the case that *k* is a kernel for this space with the property, according to the Representer Theorem, that solutions to the regularised empirical risk given in Eq (7) take the form

$$\begin{array}{c} f\left(\cdot \right)=\sum_{i=1}^{N}\alpha_{i}k\left(\cdot ,t_{i}\right) \end{array}$$

for $\alpha_{i}\in R$ for all *i* ∈ {1, …, *N*}. The conditions under which such a representer theorem exists are studied in [63].

Given these results one may then link the estimation problem for representing each IMF to the case of polynomial smoothing splines, used to represent the IMF basis functions under the EMD method proposed. To see this consider, without loss of generality T=[0,1], penalty function $J(\gamma_{l})=\int_{0}^{1}{\left(\gamma_{l}^{\left(m\right)}\left(t\right)\right)}^{2}dt$ which acts to penalise irregularity and induce smoothness in the spline representation of IMF basis. One can then construct an RKHS whose norm corresponds to this smoothing penalty *J*. Hence, the kernel needs to be made explicit.

Using Taylor’s theorem in one dimension with integral remainder term to express the IMF function *γ*_*l*_, which is assumed to have at least *m* − 1 order absolutely continuous derivative in [0, 1] and $\gamma_{l}^{\left(m\right)}\in L^{2}\left[0,1\right]$, then

$$\begin{array}{c} \gamma_{l}\left(t\right)=\sum_{i=1}^{m-1}\frac{t^{i}}{i!}\gamma_{l}^{\left(i\right)}\left(0\right)+\int_{0}^{1}\frac{{\left(t-s\right)}_{+}^{m-1}}{(m-1)!}\gamma_{l}^{\left(m\right)}\left(s\right)ds, \end{array}$$

where (⋅)_+_ is the positive part only and zero otherwise. If functions with this series representation with the first *m* − 1 derivatives being 0 at *t* = 0 are denoted by $W_{m}^{0}$, then for $\gamma_{l}\in W_{m}^{0}$ one has

$$\begin{array}{c} \gamma_{l}\left(t\right)=\int_{0}^{1}G_{m}\left(t,s\right)\gamma_{l}^{\left(m\right)}\left(s\right)ds \end{array}$$

where $G_{m}\left(t,s\right):={\left(t-s\right)}_{+}^{m}/\left(m-1\right)!$. Now observe that one can obtain an RKHS space from $W_{m}^{0}$ with the inner product

$$\begin{array}{c} \left\langle f,g\right\rangle =\int_{0}^{1}f^{\left(m\right)}\left(s\right)g^{\left(m\right)}\left(s\right)ds \end{array}$$

and kernel $k_{1}\left(t,s\right)=\int_{0}^{1}G_{m}\left(t,r\right)G_{m}\left(s,r\right)dr$. Now if one defines the null space of the penalty function as $H_{0}=span({\left\{\varphi_{i}\left(t\right)\right\}}_{i=1}^{m})$ with *φ*_*i*_(*t*) = *t*^*i*−1^/(*i* − 1)!. Then the kernel for $H_{0}$ is $k_{0}\left(t,s\right)=\sum_{i=1}^{m}\varphi_{i}\left(s\right)\varphi_{i}\left(t\right)$. As shown in [60] the space $W_{m}$ of functions with *m* − 1 absolutely continuous derivatives and *m* derivatives can be written as a direct sum $H=H_{0}⊕W_{m}^{0}$ with kernel *k* = *k*_1_ + *k*_0_. Furthermore, *J*(*γ*_*l*_) will be the square norm of the projection *Pγ*_*l*_ of *γ*_*l*_ onto $W_{M}^{0}$ so the PRSS estimation objective in Eq (7) with $J\left(\gamma_{l}\right)=\int_{0}^{1}{\left(\gamma_{l}^{\left(m\right)}\left(s\right)\right)}^{2}ds$ becomes

$$\begin{array}{c} Q(\gamma_{l})=\sum_{i=1}^{N}L(y_{i},\gamma_{l}(t_{i}))+\lambda {∥P\gamma_{l}∥}^{2} \end{array}$$ (8)

for $\gamma_{l}\in H$. By Representer Theorem, the solution is the generalised form given by

$$\begin{array}{c} \gamma_{l}^{\lambda }\left(s\right)=\sum_{i=1}^{N}\alpha_{i}k_{1}(s,t_{i})+\sum_{j=1}^{m}\beta_{j}\varphi_{j}\left(s\right) \end{array}$$

is comprised of two parts: an unpenalized component of $H_{0}$ and a linear combination of the projections onto $W_{m}^{0}$ of the representers of evaluation at the N time points *t*_1_, …, *t*_*N*_. For the squared error loss *L*(*y*_*i*_, *γ*_*l*_(*t*_*i*_)) = *L*(*y*_*i*_ − *γ*_*l*_(*t*_*i*_))^2^ the solution corresponds to the natural polynomial spline, see discussion in [64].

Hence, we have been able to motivate the spline representation of the IMF as the solution to a generalised estimation problem in an RKHS regularised function space. Now we will endeavour to connection this through the RKHS theory to the Gaussian process embedding.

### 4.2 Relating spline representations of an IMF and a gaussian processes stochastic embedding

Now we will treat Γ_*l*_(*t*) as a random function modelled by a GP and we will illustrate the mathematical connection between the spline representation on the pathwise EMD method decomposition of an IMF and the stochastic embedding developed in this work via GP models.

For Gaussian process prediction with likelihoods that involve the observed values of the IMF *γ*_*l*_ at *N* training points, extracted by the EMD method sifting algorithm, the empirical loss *L*(*y*_*i*_, *γ*_*l*_(*t*_*i*_)) can be expressed according to the negative log-likelihood. Then the analog of the representer theorem, as detailed in [65] is given as follows.

Since the predictive distribution of Γ_*l*_(t_*_) at test point t_*_ given observations *y*_1_, …, *y*_*N*_ is given by

$$\begin{array}{c} 0.8p(\gamma_{l}\left(t_{*}\right)|y_{1},\ldots ,y_{N})=\int p(\gamma_{l}\left(t_{*}\right)|\gamma_{l}\left(t_{1}\right),\ldots ,\gamma_{l}\left(t_{N}\right))p(\gamma_{l}\left(t_{1}\right),\ldots ,\gamma_{l}\left(t_{N}\right)|y_{1},\ldots ,y_{N})d\gamma_{l}\left(t_{1}\right)\ldots d\gamma_{l}\left(t_{N}\right) \end{array}$$

which in the GP case is expressed in terms of the GP covariance kernel *k* by

$$\begin{array}{c} \begin{array}{cc} E[\gamma_{l}\left(t_{*}\right)|y_{1},\ldots ,y_{N}] & ={\left[k\left(t_{*},t_{1}\right),\ldots ,k\left(t_{*},t_{N}\right)\right]}^{T}K^{-1}E[\gamma_{l}\left(t_{1}\right),\ldots ,\gamma_{l}\left(t_{N}\right)|y_{1},\ldots ,y_{N}]\\ & =\sum_{i=1}^{N}\alpha_{i}k\left(t_{*},t_{i}\right) \end{array} \end{array}$$ (9)

with $\left[\alpha_{1},\ldots ,\alpha_{N}\right]=K^{-1}E[\gamma_{l}\left(t_{1}\right),\ldots ,\gamma_{l}\left(t_{N}\right)|y_{1},\ldots ,y_{N}]$ where *K* is the *N*×*N* Kernel matrix (Gram matrix).

One then obtains the regularized solution to Eq (7) from a GP perspective by noting that for the specific choice of loss and penalty given by

$$\begin{array}{c} Q(\gamma_{l})=\frac{1}{\sigma_{N}^{2}}\sum_{i=1}^{N}{\left(y_{i}-\gamma_{l}\left(t_{i}\right)\right)}^{2}+\frac{1}{2}{∥\gamma_{l}∥}_{H}^{2} \end{array}$$

where the loss function is set to the negative log-likelihood in which $\sigma_{N}^{2}$ is the Gaussian noise model variance. The solution for the estimated IMF using this regularized estimation produces ${\hat{\gamma }}_{l}={\text{argmin}}_{\gamma_{l}}Q(\gamma_{l})$ which if one substitutes $\gamma_{l}\left(t\right)=\sum_{i=1}^{N}\alpha_{i}k\left(t_{*},t_{i}\right)$ and uses the fact of RKHS space $\langle k\left(\cdot ,t_{i}\right),k{\left(\cdot ,t_{j}\right)}_{H}=k\left(t_{i},t_{j}\right)$ can be re-expressed by an estimation objective explicitly in terms of the GP model as follows:

$$\begin{array}{c} \begin{array}{cc} Q(\alpha ) & =\frac{1}{2}\alpha^{T}K\alpha +\frac{1}{2\sigma_{N}^{2}}{\left|y-K\alpha \right|}^{2}\\ & =\frac{1}{2}\alpha^{T}(K+\frac{1}{2\sigma_{N}^{2}}K^{2})\alpha -\frac{1}{\sigma_{N}^{2}}y^{T}Ky+\frac{1}{2\sigma_{N}^{2}}y^{T}y. \end{array} \end{array}$$

Rewriting the objective in this manner expresses it as a parameter optimization problem in terms of coefficient vector ***α***, this is the advantage of knowing that a Representer Theorem can be applied. If one then minimizes *Q* w.r.t. vector of coefficients ***α*** one obtains

$$\begin{array}{c} \hat{\alpha }={\left(K+\sigma_{N}^{2}I_{N}\right)}^{-1}y \end{array}$$

which gives the prediction at test point t_*_

$$\begin{array}{c} \gamma_{l}\left(t_{*}\right)={\left[k\left(t_{*},t_{1}\right),\ldots ,k\left(t_{*},t_{N}\right)\right]}^{T}{\left(K+\sigma_{N}^{2}I_{N}\right)}^{-1}y \end{array}$$

which is exactly the predictive mean given in Eq (9).

Now to explicitly recover the solution to the smooth spline interpolation for the IMF representation obtained via solving Eq (8) using *m* = 2 and the regularised GP solution just presented we can use the result of [66] which shows that in this case if one considers a random function representation of the IMF given by

$$\begin{array}{c} \gamma_{l}\left(t\right)=\sum_{j=0}^{1}\beta_{j}t^{j}+f\left(t\right) \end{array}$$

where $\beta ∼N(0,\sigma_{\beta }^{2}I)$ and *f*(⋅) a GP with covariance $\sigma_{f}^{2}k_{sp}\left(t,t^{\prime }\right)$ given by

$$\begin{array}{c} k_{sp}\left(t,t^{\prime }\right)=\int_{0}^{1}{\left(t-s\right)}_{+}{\left(t^{\prime }-s\right)}_{+}ds=\frac{|t-t^{\prime }|\text{min}{\left(t,t^{\prime }\right)}^{2}}{2}+\frac{\text{min}{\left(t,t^{\prime }\right)}^{3}}{3}. \end{array}$$

Then to complete the example of the regularizer in the cubic spline case, we must remove penalties on polynomial terms in the null space by making taking *σ*_*β*_ → ∞. This produces the final predictive mean solution for the GP representation of the cubic spline characterisation of the IMF given by

$$\begin{array}{c} {\bar{\gamma }}_{l}\left(t_{*}\right)={\left[k\left(t_{*},t_{1}\right),\ldots ,k\left(t_{*},t_{2}\right)\right]}^{T}K_{y}^{-1}(y-H^{T}\bar{\beta })+{\left[(1,t_{*})\right]}^{T}\bar{\beta } \end{array}$$

with Kernel covariance matrix *K*_*y*_ corresponding to elements $\sigma_{f}^{2}k_{sp}\left(t_{i},t_{j}\right)+\sigma_{N}^{2}\delta_{ij}$ evaluated at all training points, *H* the matrix collecting the vector of polynomial basis terms (1, *t*) at training points and kernel least squares coefficient estimator given by

$$\begin{array}{c} \bar{\beta }={\left(HK_{y}^{-}1H^{T}\right)}^{-1}HK_{y}^{-1}y. \end{array}$$

From this solution, one can see that the resulting solution for the predictive mean function for the GP representation of the IMF for *γ*_*l*_ will have a cubic polynomial form.

### 4.3 Gaussian processes based stochastic EMD embeddings

Having established how the GP representations is connected mathematically to the empirical path-wise cubic spline representation for an IMF in the EMD method, we now generalise the stochastic embedding from a single IMF to the entire collection of IMFs under two different system models proposed. Each of these will be designed to satisfy the properties proposed for the stochastic embedding objectives set out in Section 3.1.

To achieve the desired embedding, consider first the stochastic process associated with the observed sampled signal converted from samples {*s*(*t*_1_), …, *s*(*t*_*N*_)} to spline $\tilde{s}\left(t\right)$ which when considered as the realisation of stochastic process will be denoted by *S*(*t*) and $\tilde{S}\left(t\right)$ respectively. The reference model used for comparison to the stochastic EMD models will involve directly modelling the process $\tilde{S}\left(t\right)$ without the EMD method signal decomposition information, via a GP model given in System Model 1 (SM1).

#### 4.3.1 System Model 1 (SM1): Gaussian process for $\tilde{S}\left(t\right)$

For SM1 there is a choice to calibrate the GP model directly to observations of the process *S*(*t*) or to set up the model alternatively as follows, using the values of $\tilde{s}\left(t\right)$ for estimation of the GP model. This second choice will often be both more aligned as a reference model to the EMD method stochastic embedding as well as more robust to noise due to the regularisation that can be adopted when obtaining $\tilde{s}\left(t\right)$. Therefore, under SM1 the GP model for signal *S*(*t*) is obtained via

$$\begin{array}{c} S\left(t\right)\overset{d}{=}\tilde{S}\left(t\right)+\epsilon \left(t\right) \end{array}$$

where we treat $\tilde{S}\left(t\right)$ as a GP

$$\begin{array}{c} \tilde{S}\left(t\right)∼GP(\mu \left(t;\psi_{\tilde{S}}\right);k\left(t,t^{\prime };\theta_{\tilde{S}}\right)), \end{array}$$ (10)

with $\mu (t;\psi_{\tilde{S}})$ and $k(t,t^{\prime };\theta_{\tilde{S}})$ representing the mean and kernel functions respectively, $\psi_{\tilde{S}}$ and $\theta_{\tilde{S}}$ are the sets of hyperparameters of the mean and the kernel respectively. The additive error *ϵ*(*t*) corresponds to a regression error based on using the spline representation $\tilde{s}\left(t\right)$ for the representation and potentially calibration of the SM1.

#### 4.3.2 System Model 2 (SM2): Gaussian processses for IMFs ${\left\{\Gamma_{l}\left(t\right)\right\}}_{l=1}^{L}$

When the EMD is applied to signal $\tilde{s}\left(t\right)$ and the set of basis functions are extracted, each IMF *γ*_*l*_(*t*) will be considered as the realised path of the stochastic process denoted as Γ_*l*_(*t*) and the one for the residual *r*(*t*) denoted as *R*(*t*). This will produce the following stochastic embedding of the EMD given:

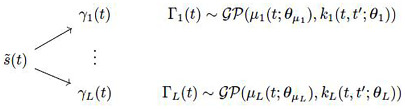

with

$$\begin{array}{c} \tilde{S}\left(t\right)\overset{d}{=}\sum_{l=1}^{L}\Gamma_{l}\left(t\right)+R\left(t\right) \end{array}$$

where *ϵ*(*t*)∼*N*(0, *σ*_*ϵ*_) and Γ_*l*_(*t*) represents the GP for IMF *l* and there are *l* = 1, …, *L* of them and *R*(*t*) represents the GP on the residual tendency component. This general structure will form the basic structure for the two stochastic embeddings proposed for the EMD method and we will refer to these two models as System Model 2 (SM2) and System Model 3 (SM3).

Therefore one can see that the resulting model is still a GP model but differs from the baseline benchmark model in Eq (10) as follows

$$\begin{array}{c} \tilde{S}\left(t\right)∼GP(\sum_{l=1}^{L}\mu \left(t;\psi_{\Gamma_{l}}\right)+\mu \left(t;\psi_{R}\right);\sum_{l=1}^{L}k\left(t,t^{\prime };\theta_{\Gamma_{l}}\right)+k\left(t,t^{\prime };\theta_{R}\right)+\sigma_{\epsilon }\delta_{t,t^{\prime }}) \end{array}$$ (11)

It is apparent that the proposed GP model for the stochastic embedding of the EMD method differs from a direct GP model on the signal as detailed in reference model directly in how the sufficient statics are designed. The key point of the stochastic embedding of the EMD method GP framework is that the kernel of the GP is now comprised of a multi-kernel framework, where each kernel can be specifically calibrated to the extracted EMD’s basis functions. Furthermore, it is trivially to verify that this stochastic embedding of the EMD method satisfies the objectives set-out in Section 3.1.

### 4.4 Treatment of the residual tendency stochastic embedding

As detailed in Section 3 last component extracted by the EMD corresponds to the residual or tendency component *r*(*t*). By definition, this last component has only one convexity within the domain [0, *T*]. Therefore, it is possible, without loss of generality, to partition it in two subregions [0, *s*] and [*s*, *T*] in which monotonicity applies locally in each. Consequently one could then impose the following structure on the GP model for *R*(*t*) over each region that enforces a stochastic monotonicity as discussed in [67], producing an isotonic restriction on the Gaussian Process. This is achieved by imposing derivative constraints on the sufficient statistics. Effectively, this utilises the fact that a derivative of a Gaussian process is a Gaussian process ([65]) and therefore a convexity constraint will result in conditions on the mean as outlined below:

$$\begin{array}{c} E[\frac{\partial \mu (t;\theta_{R})}{\partial t}]=\{\begin{array}{cc} \frac{\partial E[R(t)]}{\partial t}>0, & \forall t\in [0,s]\\ \frac{\partial E[R(t)]}{\partial t}<0, & \forall t\in (s,T]. \end{array} \end{array}$$

One can then consider to impose these conditions at all out-of-sample points *R*(t_*_) in such a manner that on average one preserves monotonicity. Given the conditional distribution for *R*(t_*_)|*R*(*t*_1_), …, *R*(*t*_*N*_) one imposes the following conditions on the predictive distribution:

$$\begin{array}{c} \begin{array}{cc} E[\frac{\partial R(t_{*})}{\partial t}|R\left(t_{1}\right),\ldots ,R\left(t_{N}\right)] & =\frac{\partial k(t_{*},t)}{\partial t_{*}}{\left(K+\sigma_{\epsilon }^{2}I\right)}^{-1}{\left[R\left(t_{1}\right),\ldots ,R\left(t_{N}\right)\right]}^{T}>0\\ Var[\frac{\partial R(t_{*})}{\partial t}|R\left(t_{1}\right),\ldots ,R\left(t_{N}\right)] & =\frac{\partial^{2}k\left(t_{*},t\right)}{\partial t_{*}\partial t_{*}}-\frac{\partial k(t_{*},t)}{\partial t_{*}}{\left(K+\sigma_{\epsilon }^{2}I\right)}^{-1}\frac{\partial k(t,t_{*})}{\partial t_{*}}>0 \end{array} \end{array}$$

where **t** = [*t*_1_, …, *t*_*N*_]^*T*^ and

$$\begin{array}{c} C\text{ov}[\frac{\partial r{\left(t\right)}^{\left(i\right)}}{\partial t},r{\left(t\right)}^{\left(i\right)}]=\frac{\partial }{\partial t}C\text{ov}[r{\left(t\right)}^{\left(i\right)},r{\left(t\right)}^{\left(i\right)}],\;\;\;\;C\text{ov}[\frac{\partial r{\left(t\right)}^{\left(i\right)}}{\partial t_{i}},\frac{\partial r{\left(t\right)}^{\left(j\right)}}{\partial t_{j}}]=\frac{\partial }{\partial t_{j}}C\text{ov}[r{\left(t\right)}^{\left(i\right)},r{\left(t\right)}^{\left(j\right)}]. \end{array}$$

There exists a second option for the stochastic embedding of EMD to treat the tendency, which involves rewriting the model in a conditional form as follows:

$$\begin{array}{c} \tilde{S}\left(t\right)|r\left(t\right)∼GP(\sum_{l=1}^{L}\mu \left(t;\theta_{\Gamma_{l}}\right)+r\left(t\right);\sum_{l=1}^{L}k\left(t,t^{\prime };\theta_{\Gamma_{l}}\right)+\sigma_{\epsilon }\delta_{t,t^{\prime }}). \end{array}$$

Under this formulation, the monotonicity of the tendency is obtained using the EMD methods pathwise extracted tendency function *r*(*t*). This is equivalent to developing an empirical Bayes formulation of the stochastic EMD embedding, see discussion in [68].

### 4.5 Adaptive band-limited IMF partitions

Consider the extracted instantaneous frequencies (IFs) *ω*_1_(*t*), *ω*_2_(*t*), …, *ω*_*L*_(*t*) which were constructed from the IMFs *γ*_1_(*t*), …, *γ*_*L*_(*t*) as described in Section 2.2. The EMD method extracts these functions in decreasing order according to the oscillation index of the IMFs, i.e. *osc*[*ω*_1_(*t*)] > *osc*[*ω*_2_(*t*)] > … > *osc*[*ω*_*L*_(*t*)], where *osc*[⋅] is an operator that counts the number of turning points ie. convexity changes of a signal. Notice, that in non-stationary settings, the number of oscillations will not correspond to particular stationarity in the frequency plane, and in fact the IMFs can have time-varying IFs that move around the frequency plane but remain ordered in general by their oscillation. Therefore, in order to use the EMD extracted IMFs for a stochastic embedding that is aligned with a traditional notion of bandwidth based analysis, we develop the concept of the Band Limited IMFs (BLIMFs). This allows for the development of a stochastic representation of an EMD signal decomposition that is guaranteed to be characteristic of a particular frequency band. This leads to the third system model (SM3) which is formulated based on the idea of aggregating the IMFs samples whose IFs lie within the same frequency band. Such newly formulated Quasi-IMFs are named band-limited IMFs and denoted as BLIMFs and are then modelled according to the same GP. To define the model, one needs first to introduce a partition rule which identifies different local frequency bandwidths.

In order to develop SM3 based on BILMFs we need to first present the formalism of what we refer to as an adaptive partition of the (time,frequency) plane based on the EMD extracted instantaneous frequencies (IFs) *ω*_1_(*t*), *ω*_2_(*t*), …, *ω*_*L*_(*t*). We will construct a partition based on the observed IF samples, denoted by ${\left\{p_{l,n}\right\}}_{l=1,n=1}^{L,N}$ where $p_{l,n}=\left(t_{n},\omega_{l}\left(t_{n}\right)\right)\in \Pi :=T\times I$ with time interval $T=[t_{0},t_{N}]$ and frequency interval $I=\left[\omega_{0},\omega_{M}\right]=[{\text{min}}_{n,l}\omega_{l}\left(t_{n}\right),{\text{max}}_{n,l}\omega_{l}\left(t_{n}\right)]$, where Π denotes the partition region. In developing the BLIMFs, a criteria and estimation objective will be established that will allow for the definition of an optimal partition, denoted by Π*, for the collection of empirical samples ${\left\{p_{l,n}\right\}}_{l=1,n=1}^{L,N}$. To define Π* we will segregate Π into an *M*×*D* partition. The partition of *M* non-overlapping bandwidths, denoted ${\left\{I_{m}\right\}}_{m=1}^{M}$, in the frequency domain satisfy

$$\begin{array}{c} I=\overset{M}{\underset{m=1}{⋃}}I_{m}\text{,}\;\text{s.t.}\;\overset{M}{\underset{m=1}{⋃}}I_{m}=\emptyset \;\text{and}\;|I|=\sum_{m=1}^{M}\left|I_{m}\right|. \end{array}$$

Within each bandwidth $I_{m}$ a time domain partition is sought, that can be unique to each bandwidth, corresponding to *D* total time partitions per bandwidth. This produces a set of time partitions for the *m*-th bandwidth given by

$$\begin{array}{c} T=\overset{D}{\underset{d=1}{⋃}}T_{m,d}\text{,}\;\text{s.t.}\;\overset{D}{\underset{d=1}{⋃}}T_{m,d}=\emptyset \;\text{and}\;|T|=\sum_{d=1}^{D}\left|T_{m,d}\right|. \end{array}$$

As noted, it is not necessary that $|T_{m,d}\left|=\right|T_{m^{\prime },d}|$ for *m* ≠ *m*′ and *m*, *m*′ ∈ {1, …, *M*}. From this formulation of time partitioned bandwidths we can arrive at a partition of Π by defining *MD* rectangles, each denoted by $\Pi_{m,d}=I_{m}\times T_{m,d}$ for *m* = 1, …, *M* and *d* = 1, …, *D* which are non-overlapping and satisfy

$$\begin{array}{c} \Pi =\overset{D}{\underset{m,d}{⋃}}\Pi_{m,d}\text{,}\;\text{s.t.}\;\underset{m,d}{⋃}\Pi_{m,d}=\emptyset \;\text{and}\;|\Pi |=\sum_{m,d}\left|\Pi_{m,d}\right|. \end{array}$$

**Fig 4:**
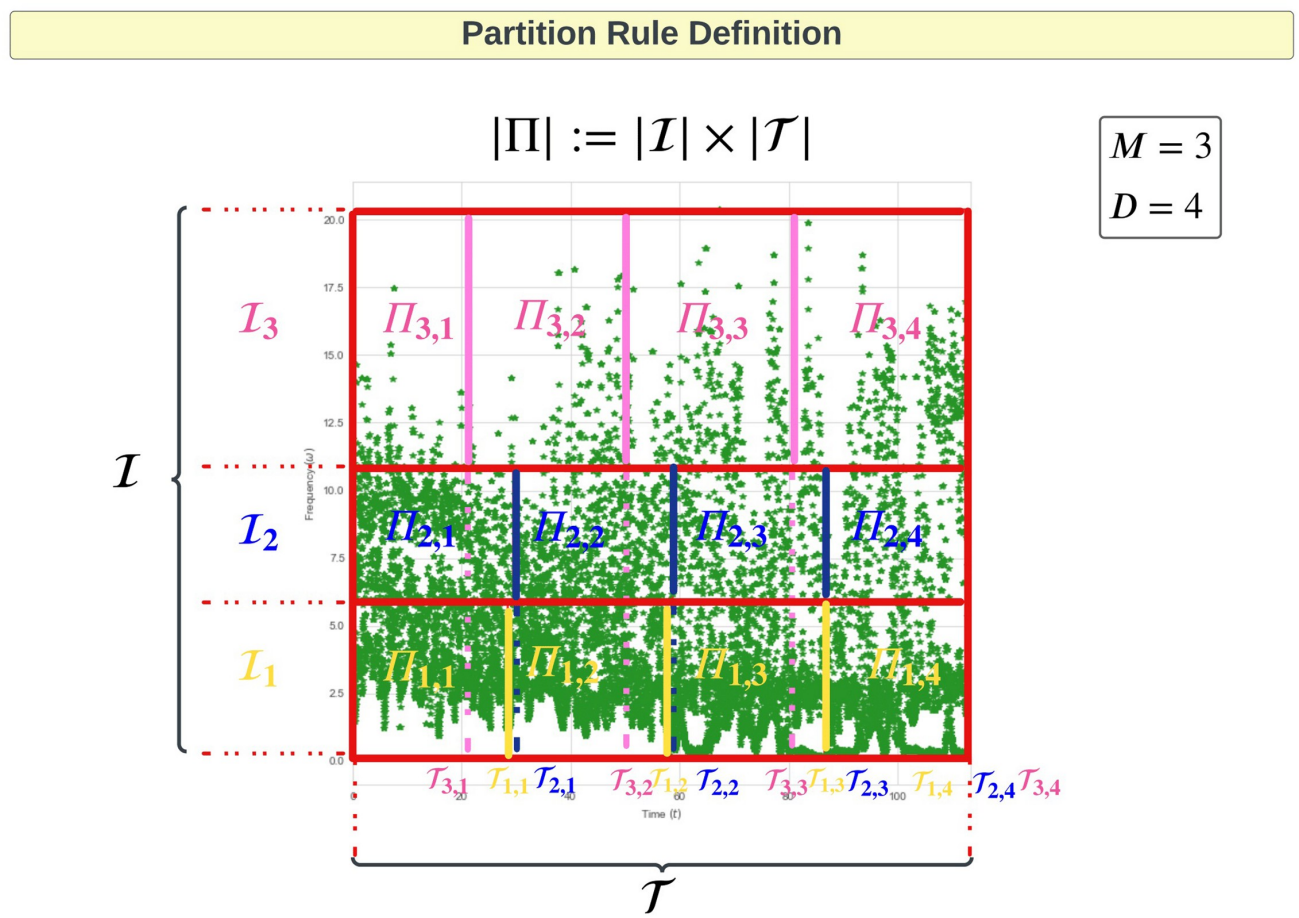
Partition Rule Definition showing how the empirical IFs samples ${\left\{p_{l,n}\right\}}_{l=1,n=1}^{L,N}$ (colored in green) within region Π are partitioned into 12 time-frequency sub-regions that are defined by running the CEM method deriving Π*. Note that, for this figure, we used only the first three IMFs, hence the first three IFs. This means that *L* = 3 in the Figure. The three IFs corresponds to the first three IFs of a speech segment used within the application of interest. Therefore, as it will be later in the paper highlighted, we consider speech segments with length *N* = 5000 samples.

See a diagramatic example of such a partition in Fig 4. In this illustration the frequency domain is partitioned into three intervals and the time domain into four intervals.

#### System Model 3 (SM3): Gaussian Processses for BLIMFs ${\left\{\Gamma_{m}^{\left(BL\right)}\left(t\right)\right\}}_{m=1}^{M}$

Given a partition Π* with *M* bandwidth we can develop the BLIMFs as follows

$$\begin{array}{c} \left\{ \begin{array}{c} \gamma_{1}^{\left(\text{BL}\right)}\left(t\right)=\gamma_{1}\left(t\right)I_{\left\{\omega_{1}\left(t\right)\in {⋃}_{d=1}^{D}\Pi_{1,d}^{*}\right\}}+\ldots +\gamma_{L}\left(t\right)I_{\left\{\omega_{L}\left(t\right)\in {⋃}_{d=1}^{D}\Pi_{1,d}^{*}\right\}}\\ \gamma_{2}^{\left(\text{BL}\right)}\left(t\right)=\gamma_{1}\left(t\right)I_{\left\{\omega_{1}\left(t\right)\in {⋃}_{d=1}^{D}\Pi_{2,d}^{*}\right\}}+\ldots +\gamma_{L}\left(t\right)I_{\left\{\omega_{L}\left(t\right)\in {⋃}_{d=1}^{D}\Pi_{2,d}^{*}\right\}}\\ \vdots \\ \gamma_{M}^{\left(\text{BL}\right)}\left(t\right)=\gamma_{1}\left(t\right)I_{\left\{\omega_{1}\left(t\right)\in {⋃}_{d=1}^{D}\Pi_{M,d}^{*}\right\}}+\ldots +\gamma_{L}\left(t\right)I_{\left\{\omega_{L}\left(t\right)\in {⋃}_{d=1}^{D}\Pi_{M,d}^{*}\right\}} \end{array}\right. \end{array}$$ (12)

these extracted BLIMFs in turn lead to the band-limited stochastic embedding of EMD method that we denoted as System Model 3 (SM3) given as follows

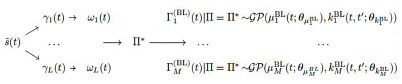

where $\Gamma_{l}^{\left(BL\right)}\left(t\right)$ denote the stochastic GP embedding of the *l*-th BLIMF. We note that since the BLIMF construction satisfies that

$$\begin{array}{c} \tilde{s}\left(t\right)=\sum_{m=1}^{M-1}\gamma_{m}^{\left(\text{BL}\right)}\left(t\right)=\sum_{i=1}^{L}\gamma_{i}\left(t\right) \end{array}$$

one can see that there will be no loss of information. However, the advantage will be in bandwidth selectivity as well as producing a frequency band-limited multi-kernel GP formulation where under SM3 one represents the stochastic process $\tilde{S}\left(t\right)$ via multi-kernel representation given by

$$\begin{array}{c} \tilde{S}\left(t\right)|\Pi^{*}\overset{d}{=}\sum_{m=1}^{M}\Gamma_{m}^{\text{(BL)}}\left(t\right)∼GP(\mu_{s}\left(t;\theta_{\mu_{s}}\right),k_{s}\left(t,t^{\prime };\theta_{k_{s}}\right)), \end{array}$$

where $\mu_{s}\left(t;\theta_{\mu_{s}}\right)=\sum_{m=1}^{M}\mu_{m}^{BL}\left(t\right)$ and $k_{s}\left(t,t^{\prime };\theta_{k_{s}}\right)=\sum_{m=1}^{M}k_{m}^{BL}\left(t,t^{\prime };\theta_{k_{M}^{BL}}\right)$.

**Fig 5:**
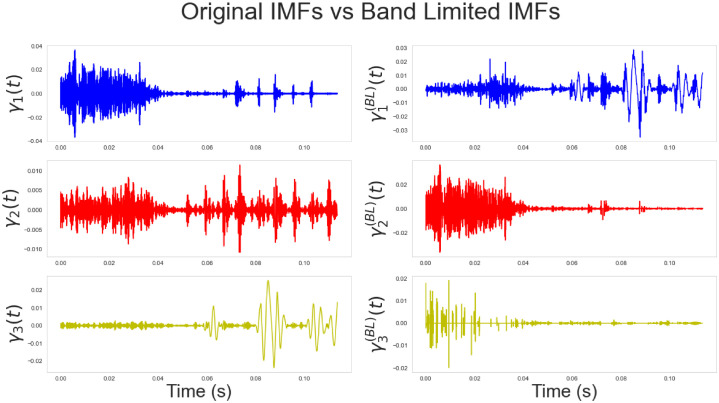
Comparison of the original extracted IMFs (left panels) and the obtained band-limited IMFs. (right panels). The original signal is a segment of the speech signals considered in section 7. The x-axis represents time and is given in seconds. It corresponds to 0.13 seconds, or, 130 milliseconds approximately (given that the speech segments is 5000 samples recorded at 44.kHz). The y-axis shows the amplitudes of the IMFs (left panels) and the band-limited IMFs (right panels).

To demonstrate such a construction, consider the illustration in Fig 5. The left panels show the first three IMFs *γ*_1_(*t*), *γ*_2_(*t*), *γ*_3_(*t*) extracted on a given speech signal. The x-axis represents the time (in seconds). Only three IMFs have been considered in this example since, for speech analysis in general, the first 3 IMFs capture the majority of the frequency content (corresponding to formant frequencies, i.e. the frequencies at which the vocal folds vibrate) required to describe, capture or classify voices in general (see [17]). The right panels present the first three BLIMFs, which are obtained according to the model given in Eq (12). It is possible to observe how the time sample points have been reassigned within a new basis since its related frequency sample points fell into a different sub-region.

## 5 Time series covariance functions for multi-kernel GP stochastic EMD embeddings

In this section we discuss how to develop a generative embedding kernel based on the Fisher kernel first proposed in [52]. This kernel family has the advantage that it can be developed to produce a time series kernel for a GP that will adapt to the local structure of the observed process being modelled. It does this through a generative embedding mechanism that transfers the observed signal into a model space and then develops a subsequent sequence of feature vectors captured by the covariance operator that makes up the kernel. When the feature vectors represent summary statistics of a fitted model over the observed signal, such as the Fisher score, one produces the Fisher kernel embedding. We will use this Fisher kernel structure for SM1, SM2 (per IMF) and SM3 (per BLIMF). We begin this section by presenting the Fisher kernel basic details. We then subsequently discuss how we obtain the partition Π* for SM3 definition of the optimal BLIMFs.

### 5.1 Generative embedding kernel

The idea of a generative embedding kernel is to map the original time series data into a model derived sequence of feature vectors that form an embedded time series representations. Think of, for instance, a time series of summary statistics. When the summary statistics are based on a model representation, this is known as a generative embedding as the model generates the feature time series upon which the GP kernel is designed from the original input time series data. In [52] a generative embedding approach was developed where the kernel used was termed a Fisher kernel. It was given this name as the final stage of the generative embedding map was determined by the gradient of the log-likelihood of the parameters of an underlying generative model, which subsequently defined a new feature space called the Fisher score space. It describes how that parameter contributes to the process of generating a particular input data. The gradient maintains all the structural assumptions that the model encodes about the generation process.

The Fisher kernel has been successfully employed within speech verification and recognition tasks by [69] and [70]. Its role in this work consists of detecting voice disturbances in displacement, direction, and velocity to differentiate between healthy and ill subjects. The adopted generative models used to produce the Fisher score feature space were intentionally kept simple and utilised basic time series models to represent the generative model embedding selected to produce the speech signal IMF based feature vectors. The model for the generative embedding of the *l*-th IMF will be denoted by *g*(*γ*_*l*_(*t*);***θ***_*k*_) with model parameters ***θ***_*k*_. Such generative models are not designed to be perfect representations of the original time series but rather to capture summary features of the IMF over time that, in turn could produce an adaptive Fisher kernel structure that could adapt locally to a time varying frequency characteristics of each IMF.

One defines the Fisher score at time *t*, denoted by $U_{\theta_{k}}\left(t\right)$ as follows:

$$\begin{array}{c} U_{\theta_{k}}\left(t\right)=\nabla \theta_{k}\text{ln}g\left(\gamma_{l}\left(t\right);\theta_{k}\right) \end{array}$$

where ∇***θ***_*k*_ denotes the gradient operator with respect to ***θ***_*k*_ of the time *t* of the log-likelihood term ln*g*(*γ*_*l*_(*t*);***θ***_*k*_). In so doing, one constructs an embedding into a generative model feature space which allows one to subsequently define the Fisher kernel via the inner product in this space:

$$\begin{array}{c} k\left(t,t^{\prime }\right)=U_{\theta_{k}}{\left(t\right)}^{⊺}\;I^{-1}\;U_{\theta_{k}}\left(t^{\prime }\right) \end{array}$$

where I is the Fisher Information Matrix $I:=E[U_{\theta_{k}}\left(t\right)\;U_{\theta_{k}}{\left(t\right)}^{⊤}]$. Hence, the Fisher score is a feature mapping such that $U_{\theta_{k}}\left(t\right)$ maps *γ*_*l*_(*t*) into a feature vector that is a point in the gradient space of the manifold $M_{\Theta_{k}}$, see [52]. The gradient $U_{\theta_{k}}\left(t\right)$ defines the direction *δ* which maximizes ln*g*(*γ*_*l*_(*t*);***θ***_*k*_) while traversing the minimum distance in the manifold given by *D*(***θ***_*k*_, ***θ***_*k*_ + *δ*), where *D*(**x**, **y**) = ‖**x** − **y**‖. This latter gradient is usually known as natural gradient and is obtained from the ordinary gradient via $\phi_{\theta_{k}}\left(t\right)=I^{-1}U_{\theta_{k}}\left(t\right)$. Hence, the mapping $\gamma_{l}\left(t\right)\to \phi_{\theta_{k}}\left(t\right)$ is called the natural mapping and the natural kernel associated to it corresponds to the inner product between these feature vectors relative to the local Riemannian metric. Note that the information matrix is asymptotically immaterial and so often one works with the simplified kernel given by setting I=I.

### 5.2 Adaptive gaussian kernel design through optimal time-frequency EMD partitions

In SM3, where the BLIMFs are used to define the inputs to the GP models, one has a choice to either select the desirable time-frequency partitions Π* based on apriori information about the signal spectrum or frequency bands of interest over time. Alternatively, in many settings, such apriori beliefs about the partition may not be available and one instead seeks an optimal partition Π* according to a desirable data-driven criterion. This section develops a solution to the optimal data-driven partition rule for SM3.

Many possible objectives could be considered. The one considered in this work is to determine the optimal partition for a given number of bandwidths that achieves empirical coverage of the sample IFs per time-frequency slot with most uniform coverage over Π. Such a partition is based on a discretised representation of the time-frequency plane that uses the IFs samples so that these can be allocated to frequency bandwidths whose distribution is as close as possible to uniform such that each band selected will have equivalent total spectral energy contributions from each BLIMF. This problem corresponds to a combinatorial search which becomes highly computational when it comes to standard optimisation techniques like simulated annealing, tabu search, MCMC algorithms. In this section an effective solution is proposed using the cross-entropy method (CEM) of [71] which has been shown to be highly effective in solving hard COPs.

**Fig 6:**
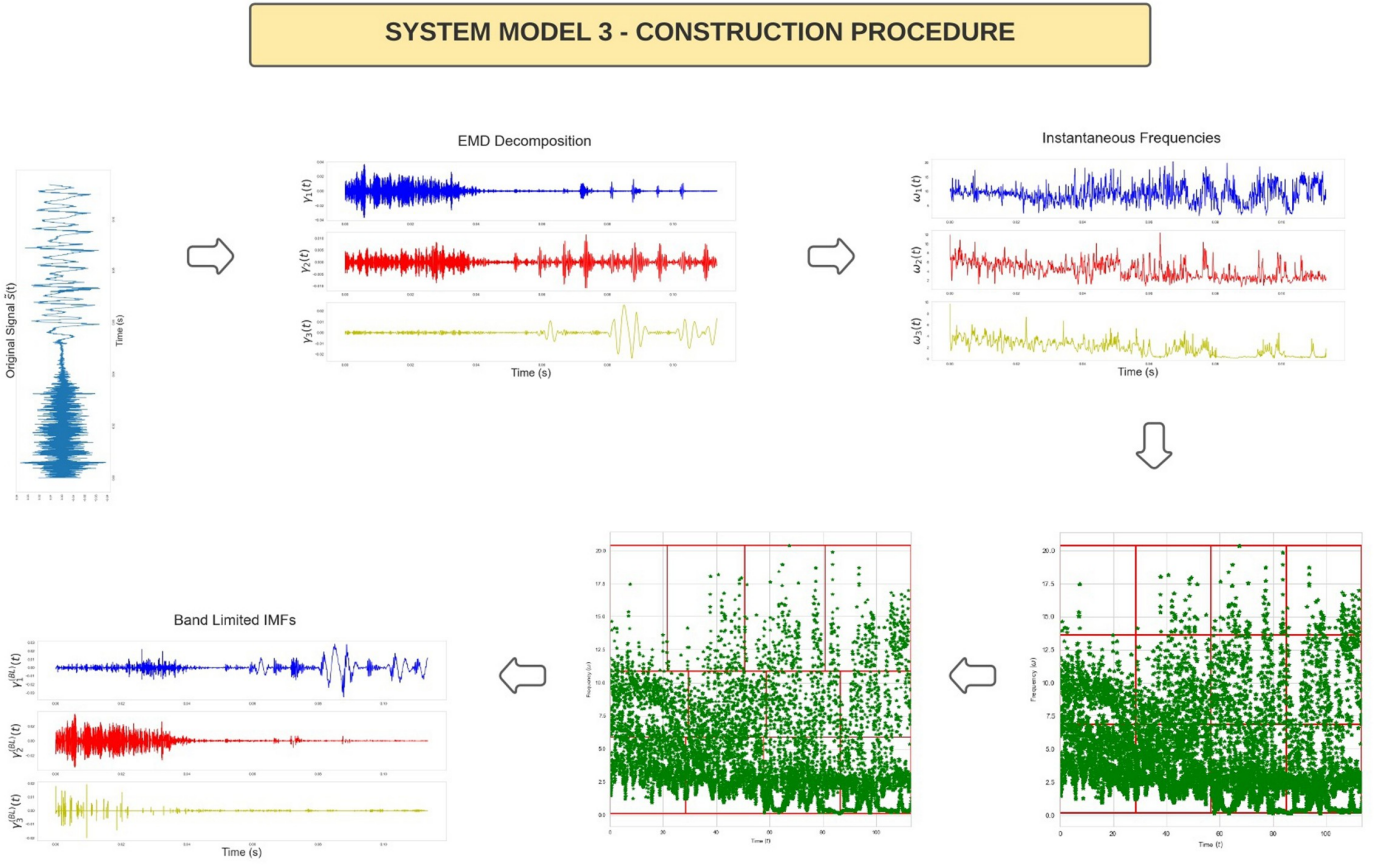
Figure presenting the steps required for the implementation of System Model 3. The first plot represents the original interpolated signal $\tilde{s}\left(t\right)$. This is a segment of speech signal used within the experiments section and corresponds to 0.13 seconds of speech. The x-axis corresponds to time (measures in seconds) and the y-axis to the amplitude. In the following plots, equivalent settings for the axes apply. Afterwards, the EMD is applied and the first three IMFs *γ*_1_(*t*), *γ*_2_(*t*), *γ*_3_(*t*) are plotted. The related IFs *ω*_1_(*t*), *ω*_2_(*t*), *ω*_3_(*t*) are extracted and plotted. After, the empirical sample points of the IFs are passed to the CEM method. The fourth step of this procedure is the initial partition Π^0^ used to initialise the cross-entropy algorithm, while the fifth step represents the CEM estimated optimal partition Π*. Lastly, the reconstructed BLIMFs are provided.

A core component of CEM is that it exploits an Importance Sampling (IS) framework to approximate the optimal solution. In the main literature of CEM minimising the Kullback–Leibler (KL) divergence, the distributions are commonly referred to as the target (true) distribution treated as an ideal model for the data (in this case, a uniform distribution) and an empirical distribution (an approximation of the true distribution), in this case, based on the empirical distribution of the sample IFs obtained from a given partition rule. An overview of the process of constructing IMFs followed by IFs then an optimal partition rule Π* via CEM followed by construction of the subsequent BLIMFs given the partition rule is provided in Fig 6.

#### 5.2.1 Formulation of the time-frequency partition optimisation problem

This subsection formalises the optimisation problem that estimates the optimal partition Π*. A given partition of Π according to *M* frequency bands is structured according to an increasing sequence of parameters *ω*_1_, …, *ω*_*M*−1_, defining frequency bandwidth subintervals of I. In addition, for each bandwith there are *D* time partitions determined, for the *m*-th bandwidth, by an increasing sequences of parameters *s*_*m*,1_, …, *s*_*m*,*D*−1_, which defines the subintervals of T. Hence, we denote the set of parameters to be estimated to determine the partition by vector:

$$\begin{array}{c} \psi =[\omega_{1},\ldots ,\omega_{M-1},s_{1,1},\ldots ,s_{1,D-1},\ldots ,s_{m,1},\ldots ,s_{m,D-1},\ldots ,s_{M,1},\ldots ,s_{M,D-1}]\in \Psi . \end{array}$$ (13)

We will next introduce the CEM importance sampling structure. Consider $X={\left\{(m,d)\right\}}_{m=1,d=1}^{M,D}$, the set of *DM* tuples and a random variable X:X→R with a target uniform density *π*(*x*) given on support X by:

$$\begin{array}{c} \text{Target:}\;\pi \left(x\right)=\prod_{m,d}\pi_{m,d}^{1_{\left\{x=(m,d)\right\}}}\;\;\text{for}\;\;\pi_{m,d}=P(X=(m,d))=\frac{|\Pi_{m,d}|}{\left|\Pi \right|}. \end{array}$$

such that the probability of drawing tuple (*m*, *d*) is proportional to the area of rectangle Π_*m*,*d*_ versus Π. Given a current estimate of the partition Π* one can also construct the empirical distribution from *N* time samples of the *L* set of IFs denoted by $\hat{\pi }\left(x\right)$ such that

$$\begin{array}{c} \text{Empirical}:\;\hat{\pi }\left(x\right)=\prod_{m,d}{\hat{\pi }}_{m,d}^{1_{\left\{x=(m,d)\right\}}}\;\;\text{for}\;\;{\hat{\pi }}_{m,d}=\hat{P}(X=(m,d))=\frac{|P_{m,d}|}{LN}, \end{array}$$

where $P_{m,d}=\{\omega_{l}\left(t_{n}\right)\in \Pi_{m,d}^{*}:l\in \{1,\ldots ,L\},n\in \{1,\ldots ,N\}\}$. Therefore, the probability of drawing tuple (*m*, *d*) reflects the proportion of the number of points *p*_*l*,*n*_ = (*t*_*n*_, *ω*(*t*_*n*_)) that lay within the rectangle $\Pi_{m,d}^{*}\subset \Pi^{*}$ to the overall sample size. Furthermore, the distribution $\hat{\pi }\left(x\right)$ is clearly then a function of the parameter vector Ψ, which has parameters that satisfy the conditions for each bandwith:

$$\begin{array}{c} \Psi =\{\begin{array}{c} \omega_{1},\ldots ,\omega_{M-1}\in \left(\omega_{0},\omega_{M}\right)\;\text{such}\;\text{that}\;\omega_{0}<\omega_{1}<\ldots <\omega_{M-1}<\omega_{M},\\ s_{1,1},\ldots ,s_{1,N_{1}-1}\in \left(t_{0},t_{N}\right)\;\text{such}\;\text{that}\;t_{0}<s_{1,1}<\ldots <s_{1,D-1}<t_{N},\\ \vdots \\ s_{m,1},\ldots ,s_{m,N_{m}-1}\in \left(t_{0},t_{N}\right)\;\text{such}\;\text{that}\;t_{0}<s_{m,1}<\ldots <s_{m,D-1}<t_{N},\\ \vdots \\ s_{M,1},\ldots ,s_{M,N_{M}-1}\in \left(t_{0},t_{N}\right)\;\text{such}\;\text{that}\;t_{0}<s_{M,1}<\ldots <s_{M,D-1}<t_{N}. \end{array} \end{array}$$

and characterise the partition Π*. From these definitions, it is clear that under these definitions one has that *π*_*m*,*d*_ and ${\hat{\pi }}_{m,d}$ are valid probabilities and satisfy

$$\begin{array}{c} \sum_{m,d}\pi_{m,d}=1\;\text{and}\;\sum_{m,d}{\hat{\pi }}_{m,d}=1. \end{array}$$

The optimization objective can then be formed under the CEM which in this problem formulation involves selecting the support of *X* in such a way that the Kullback-Leibler divergence,

$$\begin{array}{c} KL\left(\hat{\pi },\pi \right)=\int_{x\in X}\pi \left(x\right)\text{log}(\frac{\pi (x)}{\hat{\pi }\left(x\right)})dx, \end{array}$$

measuring the similarity between the two proposed distributions target and empirical partitioned density, is minimised based on determining an optimal choice of the parameters that define the partition *ψ*^⋆^, given as follows:

$$\begin{array}{c} \psi^{*}=\underset{\psi \in \Psi }{\text{argmin}}KL\left(\hat{\pi },\pi ;\psi \right)=\underset{\psi \in \Psi }{\text{argmax}}-KL\left(\hat{\pi },\pi ;\psi \right) \end{array}$$ (14)

Since this is a discrete problem, this objective can be simplified as follows:

$$\begin{array}{cc} KL(\hat{\pi },\pi ;\psi ) & =\sum_{m=1}^{M}\sum_{d=1}^{d}\pi (x=(m,d))\text{log}(\frac{\pi (x=(m,d))}{\hat{\pi }(x=(m,d))})\\ & =\text{log}LN-\text{log}|\Pi |+\frac{1}{\left|\Pi \right|}\sum_{m=1}^{M}\sum_{d=1}^{d}\{|\Pi_{m,d}|(\text{log}|\Pi_{m,d}\left|-\text{log}\right|P_{m,d}|)\}. \end{array}$$ (15)

The derivation is provided in SI, section 6 in S1 File.

#### 5.2.2 Kernel density estimator smoothing of kullback-leibler divergence in optimal partitioning problem

For a given current estimate of the partition Π*, it can arise for a given emprical sample of the IFs that certain sub-rectangles $\Pi_{m,d}^{*}$ might not contain any of the sample points **p**_*l*,*n*_ = (*t*_*n*_, *ω*_*l*_(*t*_*n*_)) ∈ Π. As a result, the corresponding set $P_{m,d}$ will be empty, i.e. $P_{m,d}=\emptyset$. Consequently, the probabilities ${\hat{\pi }}_{m,d}\left(x\right)=\frac{|P_{m,d}|}{LN}$ equal zero and their logarithms used to calculate $KL(\hat{\pi },\pi ;\psi )$ in Eq (15) tend to infinity. To avoid these numerical difficulties one can approximate ${\hat{\pi }}_{m,d}\left(x\right)$ by a kernel density estimator ${\hat{\pi }}_{m,d}^{e}\left(x;k,h\right)$ parametrised by kernel k:Π×Π→R and bandwidth *h* > 0 such that

$$\begin{array}{c} {\hat{\pi }}_{m,d}^{e}\left(x;k,h\right)=\int_{\Pi_{m,d}}\hat{\pi }\left(p;k;h\right)dp=\int_{\omega_{m-1}}^{\omega_{m}}\int_{s_{m,d-1}}^{s_{m,d}}\hat{\pi }\left(p;k;h\right)dp, \end{array}$$

where $\hat{\pi }\left(p;k;h\right):\Pi \to \left[0,1\right]$ is a kernel density estimator of points **p** = (*t*, *ω*(*t*)) ∈ Π specified on a sample set **p**_*n*,*l*_

$$\begin{array}{c} \hat{\pi }\left(p;k;h\right)=\frac{1}{Nh}\prod_{n=1}^{N}\prod_{l=1}^{L}k(\frac{p-p_{n,l}}{h})\;\;\text{such}\;\text{that}\;\int_{\Pi }\hat{\pi }\left(p;k,h\right)dp=1. \end{array}$$

By using the above, the objective function of the partitioning problem in (15) is reformulated to be the Kullback-Leibler divergence between *π*(*x*) and

$$\begin{array}{c} {\hat{\pi }}^{e}\left(x;k,h\right)=\prod_{m,d}{\left({\hat{\pi }}_{m,d}^{e}\left(x;k,h\right)\right)}^{1_{\left\{x=(m,d)\right\}}}, \end{array}$$ (16)

given by

$$\begin{array}{c} \begin{array}{cc} KL\left(\psi \right):=KL\left({\hat{\pi }}^{e},\pi ;\psi \right) & =\int_{x\in X}\pi \left(x\right)\text{log}(\frac{\pi (x)}{{\hat{\pi }}^{e}(x;k,h)})dx\\ & =\sum_{m=1}^{M}\sum_{d=1}^{d}\pi (x=(m,d))\text{log}(\frac{\pi (x=(m,d))}{{\hat{\pi }}^{e}\left(x=\left(m,d\right);k,h\right)})\\ & =-\text{log}|\Pi |-\text{log}C\\ & +\frac{1}{\left|\Pi \right|}\sum_{m=1}^{M}\sum_{d=1}^{d}\left|\Pi_{m,d}\right|(\text{log}|\Pi_{m,d}|-\text{log}\frac{{\hat{\pi }}^{e}\left(x=\left(m,d\right);k,h\right)}{C}) \end{array} \end{array}$$ (17)

with *C* > 0 and set to a very small number, ie *C* = 10^−100^. The derivation of the above is provided in SI section 7.

### 5.3 Stochastic optimisation of optimal time-frequency partition via cross entropy

Given the formulated objective function for the partition problem defined in (14) one can now define the CEM approach to stochastic optimisation used to solve for the optimal partition given the IFs. Recall, such an objective utilises the *KL*(⋅) divergence as a similarity measure between two distributions, empirical and target. This must be optimised with respect to the vector of parameters *ψ*. The CEM process to undertake this stochastic optimisation is developed by considering the level sets of the objective function {*ψ*: *KL*(*ψ*)≥*ζ*} for ζ∈R, such that at the point that $\zeta =\hat{KL}={\text{argmax}}_{\psi \in \Psi }KL\left(\psi \right)$, we have {*ψ*: *KL*(*ψ*)≥*ζ*} = {*ψ*^⋆^}. We can formulate the importance sampling solution to achieving this outcome through a sequence of *K* intermediate solutions each based on a progressively less relaxed level set constraint i.e. *ζ*_1_ < *ζ*_2_ < ⋯ < *ζ*_*K*_ where $\zeta_{K}\approx {\text{argmax}}_{\psi \in \Psi }KL\left(\psi \right)$ and at each iteration one updates the importance distribution to increase the chance of sampling solutions that are feasible according to the current level set constraint. Next we define the IS formulation of the CEM stochastic optimisation solution. This will involve defining an IS sampling distribution for the parameters **ψ** as given in Eq (13) that make up the specification of the current estimate of the optimal partition Π*. In order to achieve this we consider a family of probability measure $\left\{P_{\varphi^{\prime }}:\varphi^{\prime }\in \Phi \right\}$ with support Ψ that admits a density {*f*_*φ*_: *φ* ∈ *Φ*} also parametrised by *φ* ∈ *Φ*. Let $E_{\varphi }$ denote the expectation taken with respect to $P_{\varphi }$. Let us fix *φ* and *ζ* and define a rare event probability problem:

$$\begin{array}{c} P_{\varphi }[KL(\psi )\geq \zeta ]=E_{\varphi }[I_{\left\{KL(\psi )\leq \zeta \right\}}]=\int_{\Psi }I_{\left\{KL(\psi )\leq \zeta \right\}}f_{\varphi }\left(\psi \right)d\psi \end{array}$$

Instead of approximating this probability naively by sampling from *f*_*φ*_, the importance sampling method is used. Let *g*_*φ*′_ denote the importance sampler with *φ*′ ∈ *Φ*. Importance sampling approximates the rare event probability by

$$\begin{array}{c} \begin{array}{cc} P_{\varphi }[KL(\psi )\geq \zeta ] & =\int_{\Psi }I_{\left\{KL(\psi )\leq \zeta \right\}}f_{\varphi }\left(\psi \right)d\psi =\int_{\Psi }I_{\left\{KL(\psi )\leq \zeta \right\}}\frac{f_{\varphi }\left(\psi \right)}{g_{\varphi^{\prime }}\left(\psi \right)}g_{\varphi^{\prime }}\left(\psi \right)d\psi \\ & =E_{\varphi^{\prime }}[I_{\left\{KL(\psi )\leq \zeta \right\}}\frac{f_{\varphi }\left(\psi \right)}{g_{\varphi^{\prime }}\left(\psi \right)}]\approx \frac{1}{S}\sum_{i=1}^{S}\{I_{\left\{KL\left(\psi^{i}\right)\leq \zeta \right\}}\frac{f_{\varphi }\left(\psi^{i}\right)}{g_{\varphi^{\prime }}\left(\psi^{i}\right)}\} \end{array} \end{array}$$

where vectors *ψ*^*i*^ for *i* = 1, …, *S* are iid samples generated from IS density *g*_*φ*′_(*ψ*). The optimal importance sampler densities (*g*_*φ*′_) parameters *φ*′ are then obtained progressively in the CEM iterations for a given level set *ζ* by:

$$\begin{array}{c} \begin{array}{cc} \varphi^{⋆} & =\underset{\varphi^{\prime }\in \Phi }{\text{argmax}}\int_{\Psi }I_{\left\{KL(\psi )\leq \zeta \right\}}f_{\varphi }\left(\psi \right)\text{log}\frac{f_{\varphi }\left(\psi \right)}{g_{\varphi^{\prime }}\left(\psi \right)}d\psi \\ & \approx \underset{\varphi^{\prime }\in \Phi }{\text{argmax}}\frac{1}{S}\sum_{i=1}^{S}I_{\left\{KL\left(\psi^{i}\right)\leq \zeta \right\}}\text{log}g_{\varphi^{\prime }}\left(\psi^{i}\right) \end{array} \end{array}$$ (18)

where vectors *ψ*^*i*^ for *i* = 1, …, *S* are iid samples generated from *f*_*φ*′(*ψ*)_. Notice that the last line of 18 corresponds to the maximum likelihood estimation (MLE) of *φ*′ when the samples are {*ψ*^*i*^: *KL*(*ψ*^*i*^)≥*ζ*}. The CEM starts from an initial sampling distribution $g_{\varphi_{0}^{⋆}}$ and iteratively updates the threshold $\hat{\zeta }$ and the sampling distribution *g*_*φ*′_. For a detailed introduction to cross-entropy, the reader should refer to [57].

### 5.4 Design of the cross entropy importance sampling distribution

In this manuscript the optimisation problem is over a discrete support and so we have utilised a Multinomial distribution for the importance sampling distribution. In order to specify this distribution, consider a discretisation of the intervals I and T. The importance sampling distribution must reflect the distribution of discrete random variables that partition the rectangle Π. Consider regular dense grids of I and T constructed by:

1. Partition of I into small *N*_*ω*_ intervals of size $\Delta_{\omega }=\frac{\omega_{M}-\omega_{0}}{N_{\omega }}$, and we define $I_{n_{\omega }}^{grid}=\omega_{0}+\left[n_{\omega }-1,n_{\omega }\right]\Delta_{\omega }$ for *n*_*ω*_ = 1, …, *N*_*ω*_, therefore $|I_{a}^{grid}|=\Delta_{\omega }$;
2. We partition T into small *N*_*τ*_ intervals of size $\Delta_{\tau }=\frac{t_{N}-t_{0}}{N_{\tau }}$, and we define $T_{n_{\tau }}^{grid}=\omega_{0}+\left[n_{\tau }-1,n_{\tau }\right]\Delta_{\tau }$ for *n*_*τ*_ = 1, …, *N*_*τ*_, therefore, $|T_{\tau }^{grid}|=\Delta_{\tau }$.

Now define the probabilistic model to partition I into *M* subintervals, $I_{m}$ for *m* = 1, …, *M* according to an (*M*)-dimensional multinomial random vector **X** with entries *X*_*m*_ on the support of {0, …, *N*_*ω*_} which indicate how many subsequent grids $I_{n_{\omega }}^{grid}$ are connected to construct partitions $I_{m}$ and corresponding break points $\omega_{m-1},\omega_{m}\in I$. Therefore, the multinomial random vector **X** models the number of grid points out of *N*_*ω*_ that belong to each of *M* intervals with probabilities of being in an interval being 0 ≤ *p*_1_, …, *p*_*M*_ ≤ 1 for $\sum_{m=1}^{M}p_{m}=1$. The distribution function of **X** is formulated as

$$\begin{array}{c} \pi \left(x;p\right)=\pi \left(x_{1},\ldots ,x_{M};p_{1},\ldots ,p_{M}\right)=\frac{N_{\omega }!}{\prod_{m=1}^{M}x_{m}!}\prod_{m=1}^{M}p_{m}^{x_{m}}. \end{array}$$

for **p** = [*p*_1_, …, *p*_*M*_]. Recall that $\sum_{m=1}^{M}X_{m}=N_{\omega }$ since **X** divides *N*_*ω*_ points into *M* subsets. For instance, for realisations of *X*_1_, Â *X*_2_ such that *x*_1_ = 2 and *x*_2_ = 5, the partitions $I_{1}=\left[\omega_{0},\omega_{1}\right]$ and $I_{2}=\left[\omega_{1},\omega_{2}\right]$ are given by

$$\begin{array}{c} \omega_{1}=\omega_{0}+\Delta_{\omega }x_{1}\;\text{and}\;\omega_{1}=\omega_{1}+\Delta_{\omega }x_{2}=\omega_{0}+\Delta_{\omega }\left(x_{1}+x_{2}\right) \end{array}$$

This example gives an intuition for the general rule

$$\begin{array}{c} \omega_{m}=\omega_{0}+\Delta_{\omega }\sum_{m^{\prime }=1}^{m}x_{m^{\prime }}\;\text{for}\;m=1,\ldots M-1. \end{array}$$

and defines the approach to sample *W*_1_, …, *W*_*M*−1_ via change of variables such that $W_{m}=\omega_{0}+\Delta_{\omega }\sum_{m^{\prime }=1}^{m}X_{m^{\prime }}$ for *m* = 1, …*M* − 1. The realisation of *W*_1_, …, *W*_*M*−1_, denoted by *ω*_1_, …, *ω*_*M*−1_, represent the break points defining partitions $I_{1},\ldots ,I_{M}$. Also, we recall that *ω*_0_ and *W*_*M*_ = *ω*_*M*_ are fixed.

We model *M* independent not identical partitions of the time-domain interval T into *D* subintervals by following the same steps. We define *M* independent multinomial random variables that are *D*-dimensional, each, denoted by $X_{m}^{\prime }$ for *m* = 1, …, *M*, which entries $X_{m,d}^{\prime }$ on the support of {0, …, *N*_*τ*_}, for *d* = 1, …, *D*, specify how many subsequent grids $T_{n_{\tau }}^{grid}$ are connected to construct partitions $T_{m,d}$ of T and determine break points $s_{m,d-1},s_{m,d}\in T$. We denote their distributions by $\pi (x_{m}^{\prime };p_{m}^{\prime })$ for $p_{m}^{\prime }=\left[p_{m,1}^{\prime },\ldots ,p_{m,D}^{\prime }\right]$ such that $\sum_{d=1}^{D}p_{m,d}^{\prime }=1$. For every *m* = 1, …, *M* this construction satisfies $\sum_{d=1}^{D}X_{m,d}^{\prime }=N_{\tau }$ and

$$\begin{array}{c} s_{m,d}=t_{0}+\Delta_{\tau }\sum_{d^{\prime }=1}^{d}x_{m,d^{\prime }}^{\prime }\;\text{for}\;d=1,\ldots ,D-1,\;m=1,\ldots M. \end{array}$$

where $x_{m,d}^{\prime }$ is a realisation of $X_{m,d}^{\prime }$. Therefore, the random variables *S*_*m*,1_, …, *S*_*m*,*D*−1_ for *m* = 1, …, *M* are defined via change of variables such that $S_{m,d}=t_{0}+\Delta_{\tau }\sum_{d^{\prime }=1}^{d}X_{m,d^{\prime }}^{\prime }$ for *d* = 1, …*D* − 1 with realisations *s*_*m*,1_, …, *s*_*m*,*D*−1_ representing the break points of the partitions $T_{m,1},\ldots ,T_{m,D}$. Again, we recall that *t*_0_ and *S*_*m*,*D*_ = *t*_*N*_ are fixed for every *m* = 1, …, *M*.

We can now connect this formulation back to the IS framework in the previous section as follows. Given this model, the joint distribution of Ψ = [*W*_1_, …, *W*_*M*−1_, *S*_1,1_, …, *S*_*M*,*D*−1_] can we written as

$$\begin{array}{c} g\left(\psi ;\varphi \right)=C\;\pi \left(x_{m};p\right)\prod_{m=1}^{M}\pi \left(x_{m}^{\prime };p_{m}^{\prime }\right). \end{array}$$

Using this IS distribution we can now rewrite the IS parameter estimation rule under CEM framework, according to Eq (18) as follows, using

$$\begin{array}{cc} \text{log}g(\psi ;\varphi )= & \text{log}C+\text{log}\left(N_{\omega }!\right)+\sum_{m=1}^{M}\{\text{log}\left(x_{m}!\right)+x_{m}\text{log}\left(p_{m}\right)\}\\ & +M\text{log}\left(N_{\omega }!\right)+\sum_{m=1}^{M}\sum_{d=1}^{D}\{\text{log}\left(x_{m,d}^{\prime }!\right)+x_{m,d}^{\prime }\text{log}\left(p_{m,d}^{\prime }\right)\}. \end{array}$$

to obtain the estimation equation for the IS parameters with constraint imposed on $P=\left[p,p_{1}^{\prime },\ldots ,p_{M}^{\prime }\right]\in \left[0,1\right]$ under a Lagrangian constrained parameter estimation given as follows:

$$\begin{array}{cc} \Lambda (P,\lambda )= & \sum_{s=1}^{S}\{1_{\left\{KL(\hat{\pi },\pi ;\psi^{\left(s\right)})\leq \gamma \right\}}(\text{log}C+\text{log}\left(N_{\omega }!\right)+\sum_{m=1}^{M}\{\text{log}\left(x_{m}^{\left(s\right)}!\right)+x_{m}^{\left(s\right)}\text{log}\left(p_{m}\right)\}\\ & +M\text{log}\left(N_{\omega }!\right)+\sum_{m=1}^{M}\sum_{d=1}^{D}\{\text{log}\left(x_{m,d}^{{}^{\prime }\left(s\right)}!\right)+x_{m,d}^{{}^{\prime }\left(s\right)}\text{log}\left(p_{m,d}^{\prime }\right)\}.)\}\\ & +\lambda (1-\sum_{m=1}^{M}p_{m})+\sum_{m=1}^{M}\lambda_{m}(1-\sum_{d=1}^{D}p_{m,d}^{\prime }). \end{array}$$

where **P** represents the IS distribution parameters to be estimated and vector $\lambda \in R^{M+1}$ are the Lagrangian multipliers. If one then seeks the First Order Conditions for this Lagrangian, one obtains the system of equations that admit a feasible solution as follows:

$$\begin{array}{c} \begin{array}{cc} & \left\{ \begin{array}{cc} & \frac{\partial \Lambda (P,\lambda )}{\partial p_{1}}=\sum_{s=1}^{S}\{1_{\left\{KL(\hat{\pi },\pi ;\psi^{\left(s\right)})\leq \gamma \right\}}\frac{x_{1}^{\left(s\right)}}{p_{1}}\}-\lambda =0\\ & \vdots \\ & \frac{\partial \Lambda (P,\lambda )}{\partial p_{M}}=\sum_{s=1}^{S}\{1_{\left\{KL(\hat{\pi },\pi ;\psi^{\left(s\right)})\leq \gamma \right\}}\frac{x_{M}^{\left(s\right)}}{p_{M}}\}-\lambda =0\\ & 1-\sum_{m=1}^{M}p_{m}=0 \end{array}\right.\\ \to & \left\{ \begin{array}{cc} & p_{1}^{*}=\frac{1}{\lambda }\sum_{s=1}^{S}\{1_{\left\{KL(\hat{\pi },\pi ;\psi^{\left(s\right)})\leq \gamma \right\}}x_{1}^{\left(s\right)}\}\\ & \vdots \\ & p_{M}^{*}=\frac{1}{\lambda }\sum_{s=1}^{S}\{1_{\left\{KL(\hat{\pi },\pi ;\psi^{\left(s\right)})\leq \gamma \right\}}x_{M}^{\left(s\right)}\}\\ & \sum_{m=1}^{M}p_{m}=1. \end{array}\right. \end{array} \end{array}$$

These solutions to the IS distribution parameter estimates can be further simplified by noting that since $\sum_{m=1}^{M}p_{m}=1$ and $\sum_{m=1}^{M}x_{m}^{\left(s\right)}=N_{\omega }$ one can obtain:

$$\begin{array}{c} \frac{1}{\lambda }\sum_{s=1}^{S}\{1_{\left\{KL(\hat{\pi },\pi ;\psi^{\left(s\right)})\leq \gamma \right\}}\sum_{m=1}^{M}x_{m}^{\left(s\right)}\}=1\Rightarrow \lambda =N_{\omega }\sum_{s=1}^{S}1_{\left\{KL(\hat{\pi },\pi ;\psi^{\left(s\right)})\leq \gamma \right\}} \end{array}$$

and finally

$$\begin{array}{c} {\hat{p}}_{m}=\frac{\sum_{s=1}^{S}\{1_{\left\{KL(\hat{\pi },\pi ;\psi^{\left(s\right)})\leq \gamma \right\}}\frac{x_{m}^{\left(s\right)}}{N_{\omega }}\}}{\sum_{s=1}^{S}1_{\left\{KL(\hat{\pi },\pi ;\psi^{\left(s\right)})\leq \gamma \right\}}} \end{array}$$ (19)

Following the same steps, we have that

$$\begin{array}{c} {\hat{p}}_{m,d}^{\prime }=\frac{\sum_{s=1}^{S}\{1_{\left\{KL(\hat{\pi },\pi ;\psi^{\left(s\right)})\leq \gamma \right\}}\frac{x_{m,d}^{{}^{\prime }\left(s\right)}}{N_{\tau }}\}}{\sum_{s=1}^{S}1_{\left\{KL(\hat{\pi },\pi ;\psi^{\left(s\right)})\leq \gamma \right\}}} \end{array}$$ (20)

Note that the support of the random variables introduced in this subsection includes zero, and this may lead to the situation that some partitions are of zero length. If that happens, the breakpoints *ω*_1_, …, *ω*_*M*_ and *s*_1,1_, …, *s*_*M*,*D*−1_ are not admissible as they may not form increasing sequence. Consequently, they do not belong to the feasible set Ψ. To address this difficulty, we may consider two procedures

1. sample directly from the conditional distribution
  $$\begin{array}{cc} & X_{1},\ldots ,X_{M}|X_{1}\neq 0,\ldots ,X_{M}\neq 0\\ & X_{1,1}^{\prime },\ldots ,X_{M,D}^{\prime }|X_{1,1}^{\prime }\neq 0,\ldots ,X_{M,D}^{\prime }\neq 0. \end{array}$$
2. sampling from the Multinomial distribution and and force non zero realisation by removing any realisations that contain 0 entry to meet the conditions of the feasible set.

An algorithm for the CEM method based on this IS distribution construction is provided in the, section 8 in S1 File.

## 6 Application: Speech based medical diagnostics

In this section, we introduce how we will adopt the aforementioned Stochastic Embedding of the EMD method into a medical signal processing application based on the diagnostics of Parkinson’s Disease. The goal is to detect ataxic speech by constructing a probabilistic model for the speech signal whose tested properties will reveal the presence or absence of acoustic feature abnormalities consistent with ataxia. Before proceeding to the experiments and the obtained results, we first review speech medical diagnostic frameworks and benchmark models used for Parkinson’s disease.

### 6.1 Comparative benchmark models for Parkinson disease speech analysis

Among the various empirical tests considered for Parkinson’s disease dysfunctions evaluation, there are also speech and voice tests, based on auditory-perceptual subjective assessments of the patient’s ability to perform a range of tasks. The standard metric designed to follow Parkinson’s disease progression, introduced in 1987, is called the “Unified Parkinson’s Disease Rating Scale” (UPDRS) [72, 73]. A UPDRS assessment produces an integer number providing information about the stage of symptoms, where speech has two explicit labels, namely UPDRS II-5 and UPDRS III-18, ranging between 0–4. The label 0 represents the less severe stage, given as “Normal speech”, and 4 is the most severe stage, given as “Unintelligible most of the time”.

One challenge with such a survey-based diagnosis is that even for expert specialist doctors, it is difficult to find standardised reference baselines. This leads to a desire for a standardised objective based on formulation of a statistical model based solution that can be used for detecting the presence of the disease and surveilling its progression, see discussion in [74]. The biomarker used in this work corresponds to formant structure in speech, and the symptoms of interest are the ones affecting the vocal tract that result in ataxic speech in people with Parkinson’s disease. Hence, the objective is to identify acoustic disturbances in displacement, direction and rate (or velocity); see discussion in [29]. For further discussion on how to detect ataxic speech symptoms in Parkinson’s disease, the given speech tasks used or the employed acoustic features the reader might refer to [9, 74, 75] as references for further description of both tasks and features.

**Fig 7:**
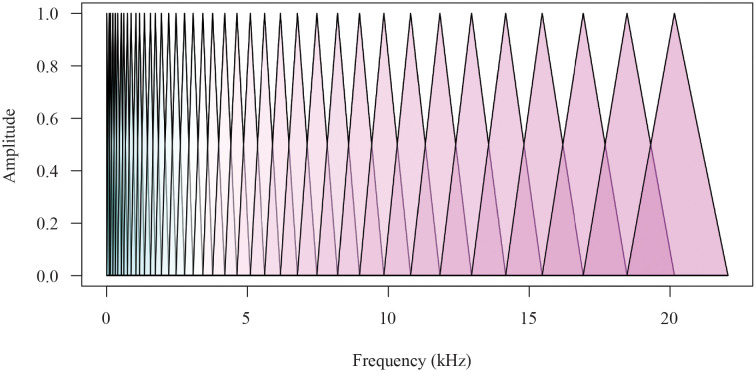
The Mel filter bank structure for 40 filters. Each peak represents the center frequency of the filters.

In speech classification tasks, numerous studies have shown that most of the discriminatory power in detecting speech variations arises from a type of individual “vocal signature” or “vocal figure print” known as the speech formants structure. Speech formants are a concentration of speech acoustic energy, usually occurring at approximately each 1,000Hz frequency band, directly related to the oscillatory modes of resonance of an individual vocal tract structure. Several alternatives can be employed to extract aspects of formant feature information, often based on basis decomposition techniques [76, 77] aiming to separate the signal into components whose frequency spectra could be preferably dominated by a single non-overlapping formant frequency. A widely used technique is to adopt warped filter basis extraction methods applied to windowed raw speech signal segments. A popular choice in practice is the Mel Frequency Cepstral Coefficients (MFCCs), see [78]. The MFCCs capture magnitude-based cepstral information, measuring the short-term power spectrum of a speech signal based on a linear cosine transform of a log power spectrum through a nonlinear mel scale of frequency [17]. This frequency scale is based on the Mel filter bank shown in Fig 7. The output of this process is a collection of functional MFCCs which captures the frequency information within several frequency bandwidths in a non-linear stationary fashion. These features have been successfully used in health diagnostics for ataxic speech ([9, 74, 75]). We are interested in the background proposed in [29]. The reader might refer to [17] for a detailed review of the MFCCs.

The main contribution of [29] is to consider phase-based cepstral features combined with the magnitude cepstrum as a human signature to detect speech abnormalities of ataxic speech. While the magnitude cepstrum has been widely used in the analysis of ataxic speech (see [79, 80]), the phase cepstrum has often been discarded for two main reasons: the difficulty in phase wrapping and the conventional view of the human auditory system as “phase deaf”. This perspective has recently changed, with several studies testifying that the change of sound phase has an instead significant impact on auditory perception [81–83]. Specifically, [29] made use of the modified group delay function (MGD) [84] to derive phase-based cepstral coefficients (MGDCCs) and combines them with magnitude cepstrum based features, i.e. the MFCCs [85, 86]. A Random Forest and an SVM framework are used to assess the discrimination power of these features in detecting ataxic speech.

The work in this paper will extend and enhance the features utilised in [29] to significantly improve the accuracy of ataxic speech symptom detection associated with Parkinson’s disease assessment in early-onset patients and its progression throughout the patients illness. We will set as the benchmark comparison the current state of the art solution of the SVM framework of [29], and we will compare our proposed EMD stochastic embedding approach combined with a tailored version of the Likelihood Ratio test to make inferences on disease state. As presented in [17] (and references within), comparing and relating such results is possible. We will further consider the background proposed by [17] and extract MFCCs on the IMFs and BLIMFs since such bases will carry the discriminant information for the performed classification task. Moreover, the bases carry less non-stationary content than the complex structure of the raw speech data, allowing for the MFCCs to be more efficient as discriminatory features in inference and testing when compared to existing methods that rely on local stationarity assumptions of Fourier-type transformations. The considered dataset, described in subsection 7.1, leads to a text-dependent environment where controls (healthy subjects) and sick patients read a given text. Reasons to employ such a specific set of sentences using the reading text task are clarified when discussing the experimental set-up in Section 7.

The other relevant feature used in [29] correspond to the MGDCCs, exploiting the modified group delay function. As studied in [42–44], the instantaneous frequency (IF) is a function assigning a frequency to a given time, whereas the group delay (GD) is a function assigning a time to a given frequency and, therefore, the question of interest here is whether the two functions are inverses of each other. In practice, this is not always the case because the IF function may not be invertible. Two conditions need to be verified for the laws of the two functions to be inverse of one another: (1) the variations in time of the IF are monotonic, and (2) the bandwidth-duration (BT) product is sufficiently large. This restricts the signals of interest to be a monocomponent signal whose IF is a monotonic function of time. Furthermore, when this is the case, the laws carry an enclosed physical meaning: the IF describes the frequency modulation of the signal, while the GD represents the time delay of the signal. Thus, when studying features based on such functions, a monocomponent signal is required, or the interpretability of the results might be misleading. Alternatively, as in our case, when such features are applied instead to the decomposed IMF basis functions after applying EMD to the speech signal, then by construction each IMF will satisfy such properties, this provides a general applicability of such interpretations from our approach, not afforded to the previous benchmark approach in general speech applications. Two of our system models (the second and the third) strongly rely on this discussion and propose stochastic embeddings based on the IMFs, which are, by definition, monocomponent functions. Furthermore, system model 3 is built upon the IFs of the IMFs. Therefore, our final aim is to provide two models distinguishing the two families of controls and Parkinson’s disease patients based on the IMFs and the IFs to depict ataxic speech.

We also include the reference benchmark features of [29] to compare our results thoroughly. These are given in Table 1. Hence, beyond MFCCs and MGDCCs, we compute the percent jitter, referring to the measurement of voice frequency perturbation, the percent shimmer, corresponding to voice amplitude perturbation, the relative average perturbation (RAP), the amplitude perturbation quotient (APQ), the pitch perturbation quotient (PPQ), the mean and the standard deviation of the cepstral peak prominence (CPP). The reader should refer to [79] for further explanations since the authors used these to detect ataxic speech for Parkinson’s disease.

**Table 1:** Description of the experimental set up. The selected benchmark features correspond to the ones of [29], i.e. MFCCs, MGDCCs, Jitter(%): frequency perturbation, Shimmer (dB): amplitude perturbation, APQ (%): amplitude perturbation quotient, PPQ (%): pitch perturbation quotient, RAP (%): relative average perturbation, CPP mean: mean of cepstral peak prominence corresponding to the mean of voice quality perturbation and CPP s.d.: variation in the cepstral peak prominence corresponding to variation in voice quality perturbation. These are extracted on the given speech signals $\tilde{s}\left(t\right)$. The configuration employed for the extraction procedure of these features are provided in subsection 7.4. Then, each system model is performed, and the GLRT is applied. Note that, SM1 is considered as benchmark model since it is the proposed reference given standard ASR direclty extract features on the raw data (as done for the bencmark introduced). Further, when it comes to SM2 and SM3, we will consider the first three IMFs or the first three BLIMFs only since they are the ones that detect the great majority of formants required for the classification of Parkinson’s disease. Both the SVM and the GLRT will be done by patient, setting up a text-dependent and a speaker-dependent environment.

Experiment Description			
System	Feature	Data	Classifier
Benchmark	MFCCs, MGDCCs, Jitter,	$\tilde{s}\left(t\right)$	SVM
	Shimmer, APQ, PPQ,	$\tilde{s}\left(t\right)$	SVM
	RAP, CPP mean, CPP s.d	$\tilde{s}\left(t\right)$	SVM
SM1	GP	$\tilde{s}\left(t\right)$	GLRT
SM2	GP-EMD	*γ*_1_(*t*), *γ*_2_(*t*)*γ*_3_(*t*)	GLRT per IMFs
SM3	GP-EMD	*γ*_1_(*t*)^(BL)^, *γ*_2_(*t*)^(BL)^, *γ*_3_(*t*)^(BL)^	GLRT per BLIMFs
SM2	EMD-MFCCs	IMF1-MFCCs	SVM per IMFs-MFCCs
		IMF2-MFCCs	
		IMF2-MFCCs	
SM3	EMD-MFCCs	BLIMF1-MFCCs	SVM per BLIMFs-MFCCs
		BLIMF2-MFCCs	
		BLIMF2-MFCCs	

### 6.2 Proposed stochastic EMD hypothesis testing framework for Parkinson’s detection

In this section, it is demonstrated how to use the GP stochastic models from SM1, SM2 or SM3 to develop a hypothesis testing framework that can be utilised to perform inference on the presence or absence of Parkinson’s disease features in speech recorded from patients. For a given system model (SM1, SM2 or SM3), the EMD method was used to extract IMFs from two different sampled populations of patients, those diagnosed at various stages of Parkinson’s disease progression vs a second population sample of healthy patients. Given the sample speech signals from each population sample, the training stage of the inference procedure involved performing EMD method on the speech signal samples, extracting IMFs and IFs, calibrating the Fisher kernel via a generative embedding model using linear time series models for each IMF, extracting the optimal IFs time-frequency partition Π* using CEM and then using the stochastic formulation of each system model SM1, SM2 or SM3 to train the subsequent GP models. Since the stochastic embedding of the EMD method under SM1, SM2, or SM3 are each based on GP models, we will be able to generically present the hypothesis testing framework as follows using a generic kernel *k*(*t*, *t*′), which will be replaced with the relevant kernel used to specify SM1, SM2 or SM3 as discussed in previous sections of this manuscript. The result of this process, described in more detail in the subsequent results section, will be an estimated representative stochastic EMD embedded GP population model for sick patients with Parkinson’s disease (distinguished by a subscripted process $\tilde{S}{\left(t\right)}_{1}$) and a corresponding estimated representative stochastic EMD embedded GP population model for the healthy patients (distinguished by a subscripted process $\tilde{S}{\left(t\right)}_{0}$) in the medical study. These were then used to develop a likelihood ratio test (LRT) hypothesis testing framework that could be utilised out-of-sample to detect unclassified patients as either not presenting with any speech disorder based symptoms consistent with Parkinson’s disease or presenting with speech disorder symptoms consistent with Parkinson’s disease. Hence, the two models that will be compared under the LRT testing framework are given by:

$$\begin{array}{c} \begin{array}{cc} {\text{Model}}_{0}:\;S_{0}\left(t\right) & ∼GP(0,k_{0}\left(t,t^{\prime }\right))\;\forall t\in \left[t_{1},t_{N}\right]\\ {\text{Model}}_{1}:\;S_{1}\left(t\right) & ∼GP(0,k_{1}\left(t,t^{\prime }\right))\;\forall t\in \left[t_{1},t_{N}\right] \end{array} \end{array}$$

This results in a null and alternative hypothesis to test given as follows:

$$\begin{array}{c} \begin{array}{c} H_{0}:\;{\tilde{S}}_{0}\left(t\right)\overset{d}{=}{\tilde{S}}_{1}\left(t\right)\;\;i.e.\;\;GP(0,k_{0}\left(t,t^{\prime }\right))=GP(0,k_{1}\left(t,t^{\prime }\right))\;\forall t\in \left[t_{1},t_{N}\right]\\ H_{1}:\;{\tilde{S}}_{0}\left(t\right)\overset{d}{\neq }{\tilde{S}}_{1}\left(t\right)\;\;i.e.\;\;GP(0,k_{0}\left(t,t^{\prime }\right))\neq GP(0,k_{1}\left(t,t^{\prime }\right))\;\forall t\in \left[t_{1},t_{N}\right] \end{array} \end{array}$$

Since a GP is also specified by its sufficient mean and covariance functions, testing for equality of distributions will be equivalent to testing for equality of the mean and covariance functions. The problem formulation in this manuscript is designed in a manner that the class of kernels utilised are restricted so that the Model_0_ is nested in the Model_1_, and hence these hypotheses can be tested with the Generalised Likelihood Ratio Test (GLRT). This is a GLRT formulation since the kernel hyper parameters are estimated. One can then obtain the test statistic by considering the log likelihood of each model under the GP stochastic embedding obtained from both the sick and healthy population samples for any of the system models (SM1, SM2 or SM3) given for samples $\tilde{s}\left(t\right)=[\tilde{s}\left(t_{1}\right),\tilde{s}\left(t_{2}\right),\ldots ,\tilde{s}\left(t_{N}\right)]$ generically by:

$$\begin{array}{c} \hat{L}=-\tilde{s}{\left(t\right)}^{⊺}{\hat{K}}_{0}^{-1}\tilde{s}\left(t\right)-\text{log}(\text{det}[{\hat{K}}_{0}])+\tilde{s}{\left(t\right)}^{⊺}{\hat{K}}_{1}^{-1}\tilde{s}\left(t\right)+\text{log}(\text{det}[{\hat{K}}_{1}]) \end{array}$$ (21)

Defining *d* as the difference in dimensionality of model parameter vectors for *H*_0_ and *H*_0_∪*H*_1_, one has an asymptotic distribution under the null hypothesis, for the test statistic given by

$$\begin{array}{c} -2\text{log}L∼X_{d}^{2} \end{array}$$

The above tests will be carried to identify the discrimination power associated with the different IMFs stochastic embedding proposed. In this way, each embedded IMF and band limited IMFs will be individually tested.

## 7 Experiments

A study of Parkinson’s speech samples is developed to assess the performance of each of the system models and their associated inference procedures presented in Section 6.2. The reference benchmark comparison will be based on the features and models introduced in [29] for the detection of ataxic speech. We aim to identify such an ataxic dysarthria symptom as a discriminative speech degradation symptom of Parkinson’s with the proposed system models for the EMD and further compare SM2 and SM3 to standard speech practices of directly applying an ASR system on the raw speech data.

We begin with an overview of the selected Parkinson’s speech dataset and its experimental setup. The first section explains the required pre-processing and the procedure for balancing the datasets since the study had an uneven number of labelled sick vs healthy patients. This is highly precious for the constructed method to avoid overfitting often occurring in ASR-SD systems. The structuring of training and testing sets is then presented. We defer the interested reader to the specialised details relating to the practical pre-processing and Fisher kernel construction methods given in the provided, sections 4 and 5 in S1 File. The validation model phase is described, and the description of our guideline reference model, introduced in 6.1 is provided. Finally, the results obtained through our proposed models are described. Table 1 shows the different features used, over which data and the corresponding classifier. The classification procedure will be conducted at a patient level, providing a text-dependent and a speaker-dependent environment. Note that the python code required for the implementation of the three system models is given within this Github page https://github.com/mcampi111, where it is possible to find a repository named “EMD-Stochastic-Embedding-for-PD-Speech” containing the code. The employed data described at [38] is given at https://zenodo.org/record/2867216#.ZAiHuRWZO3B.

### 7.1 Data description and experimental set up

The speech dataset considered for the analysis was provided by [38]. It contains speech recordings from two populations: healthy participants and patients affected at various stages of Parkinson’s disease progression. The recording environment uses a typical examination room for UK medical practices with dimensions of ten square meters in area and a reverberation time of approximately 500ms to perform the voice recordings. The voice recordings are performed in the realistic situation of doing a phone call and have been performed within the reverberation radius; hence, they can be considered “clean”. The sampling rate is standard for speech at 44.1 kHz and a bit depth of 16 Bit (audio CD quality).

The dataset is split between two sets of recordings: in the first one, the selected participants are asked to make a phone call and then read out two tests: “The North Wind and the Sun” and “Tech. Engin. Computer applications in geography snippet”. These were selected in the experimental design described in [87] since the first contains poetic structures and the second contains technical jargon, both of which are less familiar to participants’ everyday text. In the second set of recordings, the participants start a spontaneous dialogue with the test executor, who asks random questions. In our case studies, we only considered the first set of recordings. Hence, the used task to assess ataxic speech in Parkinson’s disease is reading a given text. The second set of recordings corresponding to spontaneous dialogue is considered highly challenging for this assessment. However, it could be employed in further research and used to study surveillance of the disease and its progression. The reader is referred to [87] for further detail on the collection process and experimental set-up used in the clinical setting.

We note that this database of speech signals was specifically selected given the quality of the recordings and its recording procedure. The procedure used is most aligned with the standard medical practice of relevance to telemonitoring solutions for remote Parkinson’s disease detection prior to requesting the patient to travel to a hospital for further in-person testing. This is useful for pre-screening those likely to need to travel for initial diagnosis as well as for analysis of the impact on speech for disease progression analysis for those living remotely from specialist care or those unable to easily travel from their house to the hospital on a regular basis.

There are 37 participants in total, of which 21 are healthy and 16 are sick, affected by Parkinson’s disease at different stage levels. Amongst the 21 healthy participants, 19 are female, while 2 are male. Of the 16 sick participants, 4 are female, and 12 are male. The dataset is therefore significantly unbalanced within both classes, i.e. healthy versus sick and male versus female. Furthermore, the Parkinson’s participants are labelled according to the following scores: the HYR score, the UPDRS II-5 score and the UPDRS III-18 score introduced in 6.1. Considering the UPDRS II-5 score, the Parkinson’s participants are classified in a range between 0 and 3 at maximum, particularly for the female patients, 2 are at a 0 stage level, and 2 are at a 1 stage level. In the case of the sick male patients, 5 male patients are at a 0 stage level, 4 patients at 1 stage level, 2 patients at 2 stage level and 1 patient at a 3 stage level. Hence, a further level of unbalancedness is introduced. Section 1 of the S1 File provides a more detailed summary of the described database. Table 2 summarises the above description. As a result, a procedure to balance the dataset and its pre-processing is presented in the following subsection.

**Table 2:** Description of the “Mobile Device Voice Recordings at King’s College London (MDVR-KCL)”. The number of speakers is 37, split between healthy and sick patients. Furthermore, the gender and the UPDRS II-5 score are introduced in the Table. It is possible to observe how unbalanced the dataset is, particularly regarding gender and the UPDRS II-5 score. For each speaker, the dataset provides two sets of recordings. In our experiments, we use the read text and set the scenario to a text-dependent one. Moreover, we conduct our analysis by patient, and therefore we will be in a speaker-dependent setting.

MDVR-KCL Dataset Description										
Parkinson’s disease Status	Healthy		Sick							
	Gender	Female	Male	Female				Male		
UPDRS II-5 score	−	−	0	1	2	3	0	1	2	3
# of Speakers	19	2	2	2	−	−	5	4	2	1

### 7.2 Pre-processing, balancing the dataset and construction of training and testing segments sets

This subsection outlines a brief description of the pre-processing performed to obtain a balanced selection of speech records for the testing and inference tasks undertaken. As noted, the recordings taken into account are the read text only for each participant. Within the recording procedure, each participant was asked to make a phone call and then read two different texts above mentioned. Each audio file corresponds to a continuous, unsegmented recording of the read text at the sampling rate was 44.1kHz. Therefore, we will have one audio file for each patient denoted as *s*(*t*). Depending on the patient, the reading order might change, and the recording lengths (due to different reading paces) vary between 73s and 203s. We removed the silence at the beginning and the end of the recordings and the initial participant’s dialogue with the interlocutor asking to start reading.

**Fig 8:**
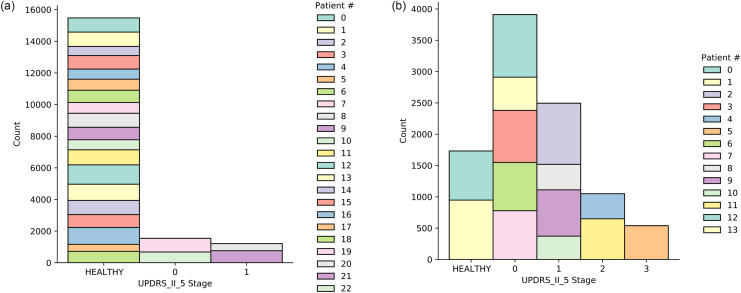
Barplots for the number of segments of length 5000 samples (approximately 0.113 seconds) for the female patients (left panels) and the male patients (right panels). The x-axis represents the different stages of the UPDRS II-5 where we also included the healthy patients. The y-axis represents the counts of the segments divided by patient.

In order to perform the EMD, the underlying signal needs to be continuous. Therefore, we fit a cubic spline with knots points placed at the sample points through each of the recordings, and we denote it as $\tilde{s}\left(t\right)$. Afterwards, we split each recording into batches of 5000 sample length for computational reasons, which approximately corresponds to 0.113 seconds (given a sample rate of 44.1kHz). Given that the audio files have different lengths, the number of resulting minibatch segments of 5000 samples for each patient differs. Fig 8 shows the number of segments for each patient divided by the scores of the UPDRS II-5 for both female (left panel) and male (right panel) patients.

As noted, one can see that the populations represented are highly unbalanced for the number of male and female patients, the different categories of the UPDRS II-5 score and the number of sick and healthy patients. To balance the representation of each patient, we compute the minimum number of segments for each patient by gender and then randomly select that minimum number of minibatches (5000 samples each batch) from each patient by sampling with replacement. We denote the minima as *N*_*f*_ and *N*_*m*_ and we have that *N*_*m*_ = 372 and *N*_*f*_ = 442. Therefore we will have *N*_*m*_×14 segments for the male patients and *N*_*f*_×23 segments for the female patients.

Once we have obtained a balanced representation of each patient with respect to the number of segments, the following step consists of constructing training and testing sets of segments for our classification task, divided into model estimation and model validation. Consider the female case as an example and note that an equivalent procedure is applied to the male case. To construct the training set, we firstly left one patient out for the testing set. Then from the remaining number of patients segments, i.e. *N*_*f*_×18 for the healthy case and *N*_*f*_×3 for the sick case, we randomly extract 80% of *N*_*f*_ corresponding to 354 segments. Hence, we will have 354 segments representing the class of healthy patients and 354 segments representing the class of sick patients, randomly extracted from 18 and 3 patients equally represented. For the testing set instead, we randomly select 20% of *N*_*f*_ from the two left out patients segments, one for the healthy and one for the sick classes, corresponding to 89 segments. Therefore, we will have 89 segments for the healthy patient left out and 89 segments for the sick patient left out. We then rotate the left out patients and repeat the procedure. This means that we perform cross-validation at a segment and a patient level, so neither class, i.e. sick or healthy, nor any patient is misrepresented in the experiments, and, as a result, over-fitting is handled as well as a fine representation of the given data. Note that, we will refer to $\tilde{s}{\left(t\right)}_{0}^{tr}$ and $\tilde{s}{\left(t\right)}_{1}^{tr}$ with *tr* = 1, …, *N*_*tr*_ for the training set and to $\tilde{s}{\left(t\right)}_{0}^{ts}$ and $\tilde{s}{\left(t\right)}_{1}^{ts}$ with *tr* = 1, …, *N*_*ts*_ for the testing set. Note that for the male case, *N*_*tr*_ = 298 and *N*_*ts*_ = 75.

### 7.3 Testing procedure for the model validation phase

The next step uses these training data sets to develop a fitting procedure which involves the construction of the generative embedding Fisher kernels from the EMD outputs as described in Section 5.1. This requires practical parts beyond the paper’s main scope, detailed in the Sections 4 and 5 in S1 File. There are two main aspects which are relevant at this point and that the reader should consider. First, the fitting procedure aims to identify fast changes that cannot be perceived by the human ear, i.e. by a doctor. Therefore, the procedure is done on mini-batches of approximately 2.2ms, meaning that each segment will be further split into mini-batches. Each mini-batch can then be characterised by a simple model whose set of hyperparameters will be informative with respect to fast changes signalling the presence/absence of the disease. Second, it is highly likely that not all mini-batches are discriminatory for such a task. Hence, a model selection criterion is required. Once a set of best discriminatory models are identified, a rule able to describe a unique family (i.e. female sick, female healthy, male sick and male healthy) of speech signals that can then be tested is required. The steps of the fitting procedure are given as follows: (1) Split the segments into mini-batches; (2) Fit a set of ARIMA models (see Section 4 in S1 File for further details on this) on each mini-batch; (3) Select the best model per mini-batch and then per segment according to the Akaike Information Criterion; (4) save the obtained model hyperparameters that will then be used to derive a Fisher score employed in the testing procedure; (5) save the proportion for each winner model, i.e. how many times a specific model for the mini-batches was selected as best over its segment. In such a way, a “weighted” rule will be defined for the definition of the Fisher score in the testing procedure. Note that we will end with *N*_*f*_ = 354 best models for the female families (i.e. both sick and healthy) and *N*_*m*_ = 298 for the male families (i.e. both sick and healthy).

The testing procedure computes the Fisher score vectors by evaluating the obtained best models on the testing data (also split by mini-batches) of each patient. By considering the healthy female case, for example, 354 models are evaluated on each mini-batch of every testing segment. In practice, one has 354 sets of hyperparameters describing one mini-batch, while the desired scenario would be having one set of hyperparameters per mini-batch. This is achieved by computing the Fisher scores for every best model per mini-batch and then aggregating them to have a unique vector testing the discriminatory power of the best models as a whole. An equivalent procedure is done for the sick female family on that same mini-batch and, therefore, one can redefine the GLRT test formulated in Eq (21) as

$$\begin{array}{c} \begin{array}{c} \hat{L}=-\left({\tilde{U}}_{\theta_{0}}^{j}\right){\left({\tilde{K}}_{0}^{j\;S}\right)}^{-1}{\left({\tilde{U}}_{\theta_{0}}^{j}\right)}^{⊺}-\text{log}(\text{det}[{\tilde{K}}_{0}^{j\;S}])\\ +\left({\tilde{U}}_{\theta_{1}}^{j}\right){\left({\tilde{K}}_{1}^{j\;S}\right)}^{-1}{\left(U_{\theta_{1}}^{j}\right)}^{⊺}+\text{log}(\text{det}[{\tilde{K}}_{1}^{j\;S}]) \end{array} \end{array}$$ (22)

**Fig 9:**
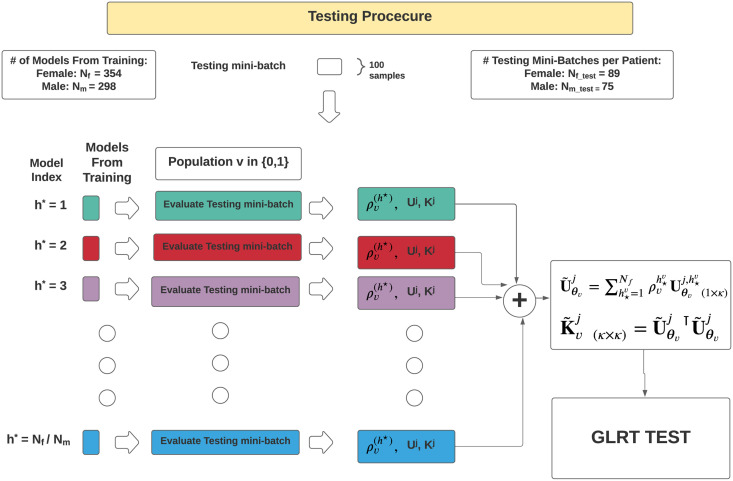
Figure showing a diagram for the steps required for the testing procedure of the model estimation phase. The GLRT test is computed on each mini-batch extracted by the segments of every patient. Note that each mini-batch is approximately 2.2.ms. The GLRT test is conducted on weighted and aggregated Fisher score vectors.

This shows that the test is done on the Fisher scores, rather than directly on the speech segments. Fig 9 shows the step of the described procedure. Furthermore, the details and derivation of such a procedure are outlined in the Section 5 in S1 File. In Eq (22), ${\tilde{U}}_{\theta_{0}}^{j}$ and $U_{\theta_{1}}^{j}$ represent the centred, weighted, aggregated Fisher scores evaluated on a testing mini-batch for healthy and sick family (of a specif gender) respectively. ${\tilde{K}}_{0}^{j\;S}$ and ${\tilde{K}}_{1}^{j\;S}$ represents the regularised Gram Matrices derived from such Fisher scores. Note that each Gram Matrix can be defined as

$$\begin{array}{c} {\tilde{K}}_{v\;\;(\kappa \times \kappa )}^{j}={\tilde{U}}_{\theta_{v}}^{j\;\;⊺}{\tilde{U}}_{\theta_{v}}^{j}\;\;\text{for}\;\;j=1,\ldots ,N_{f,t} \end{array}$$

where *v* ∈ {0, 1}. The Gram Matrix regularisation is needed since computational instability could be encountered with the inversion of such a matrix or the log-determinant and corresponds to the covariance shrinkage estimator. Once the Gram Matrices are regularised, we added the superscript “S” for notational correctness. For further details, see the section 5 in S1 File. Once the GLRT has been done on each mini-batch of every segment, then the accuracy has been computed since this is a supervised learning procedure where we know in advance the labels of each segment. The results of the accuracy are provided in Tables 4 and 6.

### 7.4 Results

In this section, we observe formant structures of the original speech signals, IMFs and BLIMFs to interpret the obtained results and the reasoning behind our proposed solutions. We first review the healthy and ill patient speech spectrograms and their quantification of acoustic energy and afterwards compare the obtained results. The results will take into account gender since male and female formants lie within different frequency bandwidths typically. We further present a subsection describing the model complexities of the IMFs and the BLIMFs to compare the differences between sick and healthy modelling features.

#### 7.4.1 Spectrograms and formant structure

**Fig 10:**
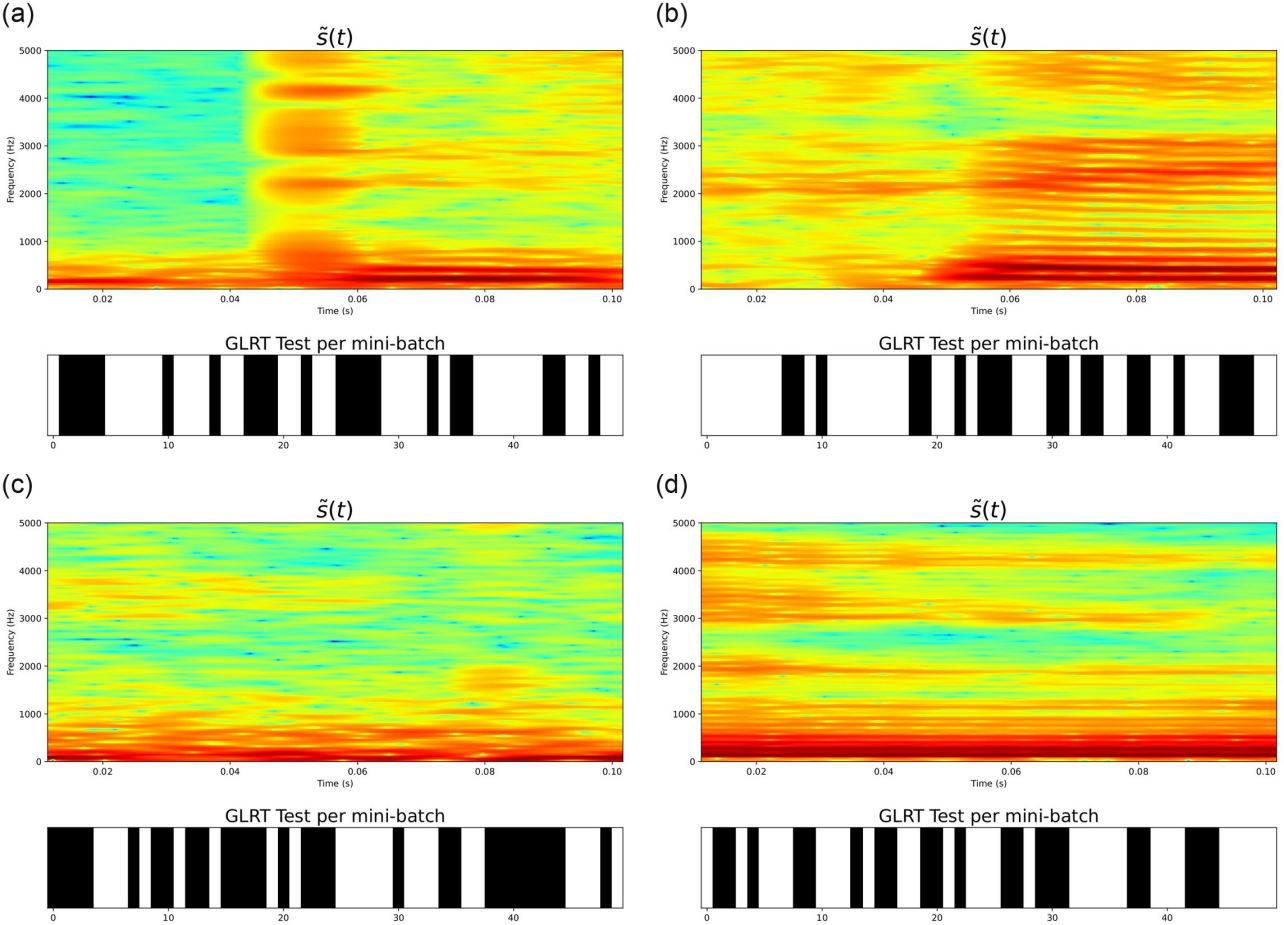
There are two panels for every plot. The top panels are spectrograms of the original speech segments for four voices. The x-axis is time (0.113 s), given in seconds, the y-axis is frequency given in Hz (0–5000Hz). The second panel represents the results of the GLRT test conducted on every mini-batch of that segment. There are 50 mini-batches per segment. White corresponds to 0 and black to 1. 0 corresponds to equality in distribution, hence no disease detected, while 1 corresponds to the detection of Parkinson’s disease. (a) Healthy female speech segment, (b) Sick female speech segment. UPDRS score equal to 1, (c) Healthy male speech segment and (d) Sick male speech segment.

Spectrograms given in Fig 10 show speech segments of 5,000 samples for four different voices: the top left panel refers to the voice of a healthy female subject, while the top right panel represents the voice of a female sick patient. The bottom panels are for male voices, healthy and sick, in the same order as above. We focus on the range of 0–5 kHz since the first five formants are visible. Hence, the y-axis varies within this range, while the x-axis represents time and is given in seconds (0.113 approximately). Focusing on the healthy subjects, the top left panel has an energy spectrum more spread out than the correspondent bottom one. This shows how, in general, female voices tend to have higher formants than male voices. Furthermore, *F*_0_, also called fundamental frequency and capturing the pitch, for male voices is more pronounced and lives within 0–1kHz, while, for female voices, it often lies at higher frequencies. This is visible in the bottom panel, where the frequency content of 0–1kHz is stronger than frequencies within the rest of the spectrum. Furthermore, formants duration over time is usually more irregular for female voices than male ones; therefore, fast changes in time will be more challenging to detect for females than males. The right spectrograms refer to speech segments of sick patients.

These plots aim to demonstrate why it is possible to accurately detect Parkinson’s disease with the proposed EMD-GP methods. One can observe the ataxic speech features present in sick patients compared to the non-ataxic speech of healthy patients. This manifests typically in clear spectral signatures that the EMD framework is able to accurately identify and then utilise in the EMD-GP testing framework for the GLRT test. Furthermore, the amount of energy intensity produced at various frequencies over time in the speech of sick patients with Parkinson’s tends to be higher than in healthy subjects. This is potentially indicative of lesser control of vocal structures used to modulate speech intensity in sick patients, consistent with patients who tend to slur or drag words.

Therefore, this paper aims to construct an effective tool able to quantify such energy changes in both domains in a data-adaptive fashion. Since the location of the formants is strongly biometric for an individual, and they carry a high level of non-stationarity, the idea is first to isolate formants through basis functions that can deal with these properties and secondly to develop a statistical methodology which quantifies formants distributions that are indeed a priori unknown. Note that, each of the shown spectrograms has a second panel below which represents the GLRT test conducted on the mini-batches of that segment and will be below discussed.

**Fig 11:**
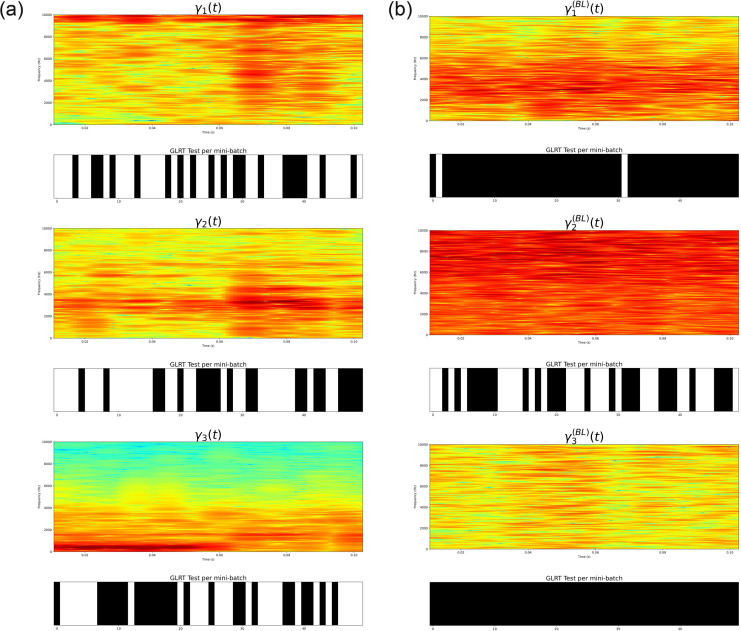
There are two panels for every plot. The top panels are spectrograms of the speech segments IMFs (left) and the BLIMFs (right) obtained from the EMD of the male speech segment given in Fig 10(d). The x-axis is time (0.113 s), given in seconds, the y-axis is frequency given in Hz (0–10000Hz). The second panel represents the results of the GLRT test conducted on every mini-batch of that IMFs or BLIMFS segment. There are 50 mini-batches per segment. White corresponds to equality in distribution, hence no disease detected, while black corresponds to the detection of Parkinson’s disease. (a) Speech segments of the first three IMFs extracted from the sick male speech segment given in Fig 10(d) and (b) Speech segments of the first three BLIMFs computed on the IMFs of the the sick male speech segment given in Fig 10(d).

If we focus on Fig 11, one can observe that there are six spectrograms. The left panels are speech segments of the first three IMFs, i.e. *γ*_1_(*t*), *γ*_2_(*t*), *γ*_3_(*t*) extracted by the speech segment related to the sick male patient in Fig 11(d). The right panels alternatively represent the spectrograms of the speech segments of the first three BLIMFs computed on the IMFs given in the left panels and denoted as $\gamma_{1}^{\text{(BL)}},\gamma_{2}^{\text{(BL)}},\gamma_{3}^{\text{(BL)}}$. This time we focused on a bigger frequency range, i.e. 0–10kHZ, to observe a broader spectrum. The figures clearly demonstrate that the first IMF captures the highest formants of the speech signal, the third and fourth formants. The second IMF detects the second formant and finally, the third IMF identifies the fundamental frequency *F*_0_. This can be observed in the left spectrograms, where the energy content decreases if one moves from the top to the bottom spectrograms.

By looking at the BLIMFs spectrograms instead, it is clear that the energy content has been reassigned within different regions since the IFs have been partitioned into an optimal partition obtained with the cross-entropy method presented in section 5.2. Indeed, $\gamma_{1}^{\text{(BL)}}$ appears to localize highest frequency content more efficiently than the basic IMF *γ*_1_(*t*). While the first IMF shows energy concentration at very high frequencies, i.e. around 9–10kHz, for most of the time, $\gamma_{1}^{\text{(BL)}}$ captures a strong energy concentration around 2kHz and 4kHz, reflecting the second and the third formants which are visible in Fig 10(d). In the case of the IMFs, these formants are split between the second basis and third basis, which detects the fundamental frequency below 2kHz. Instead, $\gamma_{2}^{\text{(BL)}}$ presents an energy spectrum which contains a lot more energy than the correspondent second IMF.

We believe that this BLIMF isolates the noise spread across the three IMFs, and, therefore, retains information that is less useful and polluted for detecting the disease. Indeed, the spectrum looks uniform in energy concentration and recalls a spectrum of the white noise signal. The last BLIMF $\gamma_{3}^{\text{(BL)}}$ cannot localize the fundamental frequency correctly. However, this is now detecting its fast frequency changes dispersed across the entire spectrum. Therefore, the CEM can find a partition identifying basis functions that provide a more efficient decomposition in formant detection.

The bottom panels of Figs 10 and 11 represent the GLRT test carried on the mini-batches of that considered speech segment, or, in the case of Fig 11, on the speech segment of the correspondent IMF or BLIMF. There are 50 mini-batches per segment; therefore, a band corresponds to 50 GLRT tests for every spectrogram. If the GLRT band is coloured in white, it indicates that the GLRT test on that mini-batch found equality in distribution and, therefore, no presence of Parkinson’s disease. In the opposite case, the GLRT test has detected differences in distributions, and it implies the detection of Parkinson’s. If one now considers Fig 10, which demonstrates the results for SM1, which does not use the EMD IMF or BLIMF structures, it is possible to observe that the GLRT performs poorly on the original data segments. It appears to detect Parkinson’s disease when there is no Parkinson’s disease since the left panels refer to the segments from healthy patients and show a GLRT band with more black tests detected in the healthy patients rather than in the sick ones. This suggests that SM1 will not perform well for the given task, which is expected given that the original signal is highly non-stationary and, therefore, challenging to model with a simple covariance function for the entire signal.

If we next consider the results for the EMD-GP model using standard IMFs, looking at the GLRT tests in Fig 11, the first two IMFs do not detect Parkinson’s disease more efficiently than the raw data. This is the case since, quite often, *γ*_1_(*t*) and *γ*_2_(*t*) capture high noise levels and, therefore, are not great candidates for performing accurate inference on disease state in the patient. Regarding IMF3, the mini-batches detecting the correct condition increase, suggesting that the fundamental frequency of male voices is a good discriminant for Parkinson’s disease detection. Such facts will be reflected in the classification results provided in Tables 4 and 6. Next, we consider the EMD-GP model using the BLIMFS. The GLRT tests of the BLIMFs perform quite differently from all the others. Particularly, the first and the third BLIMFs show perfect performances since every mini-batch (except for only two of them in $\gamma_{1}^{\left(\text{BL}\right)}\left(t\right)$) is classified correctly. Furthermore, the second BLIMF performs less effectively, suggesting that the noise affecting the formants structure can be isolated for a more discriminant decomposition. This is highly encouraging for the newly defined basis functions and will be further analysed in the discussion sections.

#### 7.4.2 Model complexity

**Fig 12:**
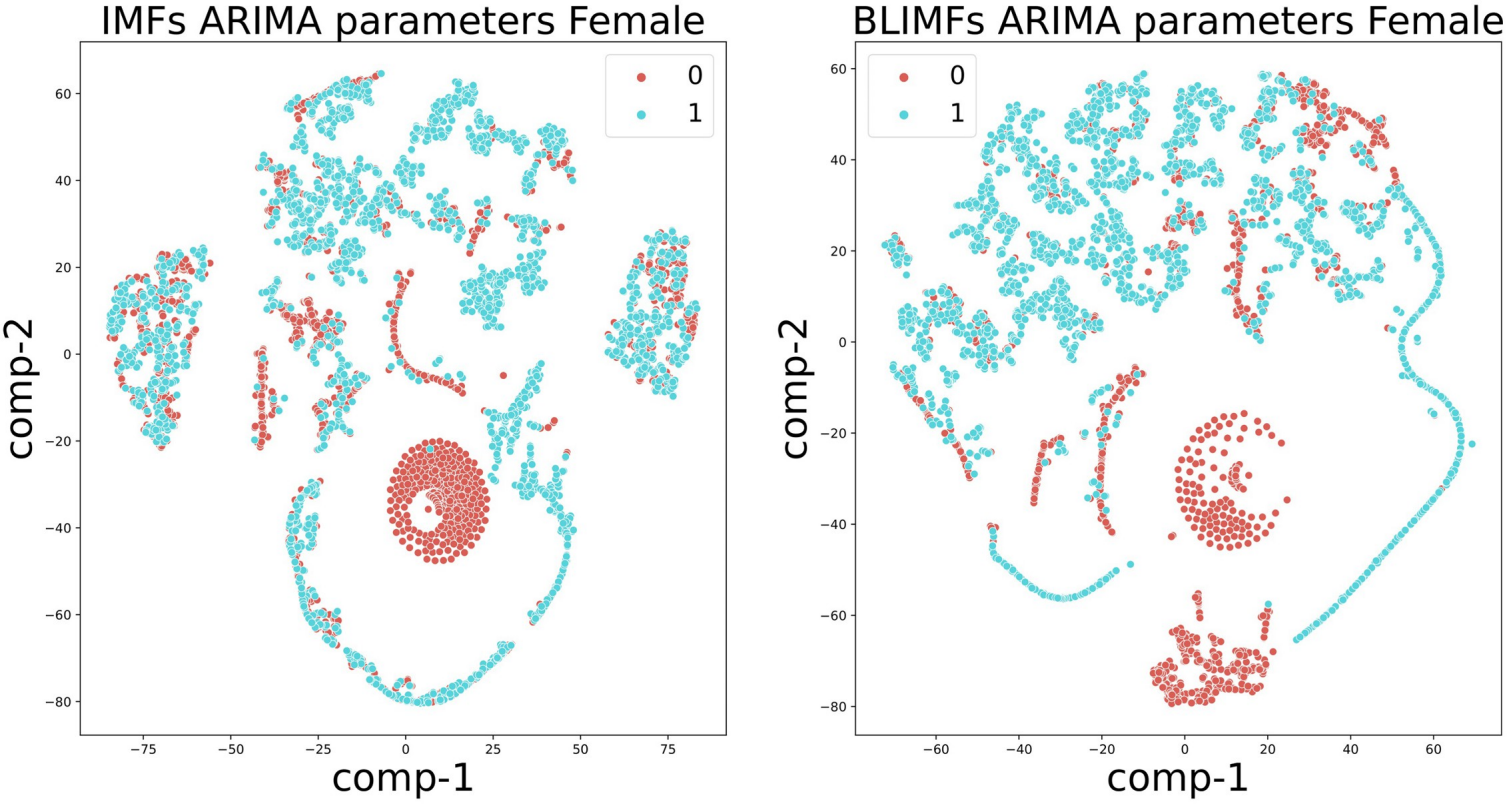
Results of t-SNE for the ARIMA parameters of the first three IMFs (left panel) and the first three BLIMFs (right panel). Note that, to run the algorithm, a PCA step was applied to reduce the initial data dimensionality, 90% of explained variation was retained. The axes represent the two dimensions identified by the t-SNE algorithm denoted as comp-1 and comp-2. Note that the azure points are denoted as 1 in the legend and refer to the parameters of the sick patients, while, the 0 points to the ones of the healthy patients.

This subsection aims to show the different model complexities provided by the computed Fisher kernel in detecting differences between healthy and sick participants according to ataxic speech feature presence or absence. Indeed, the computation of the Fisher kernel is obtained by fitting a set of nested ARIMA models with different model orders and parameter estimates. The details of the fitting procedure are provided in the Supplementary Information in detail. Hence further to the spectrograms and how these capture formant features, our idea is to present how the IMFs and the BLIMFs differentiate between speech affected by Parkinson’s vs healthy unaffected speech. To achieve such a goal, we first show Fig 12. The figure presents two panels, the left one related to the IMFs (the first three) and the right one concerning the BLIMFs (the first three again). We used the parameters of the ARIMA model fitted on these basis functions and ran two separate algorithms for visualisation purposes of this high dimensional feature space. We are able to obtain such visualisations of the high dimensinal porjections to two dimensions, showing the sub-space of optimal discriminatory structure from our EMD embeddings, between healthy and sick patient voice features, via the t-distributed stochastic neighbour embedding (t-sne), introduced by [88]. This algorithm constructs a probability distribution over pair of input data objects (the IMF feature embeddings) so that similar data are assigned a higher probability while dissimilar data has a lower probability. Afterwards, a similar probability distribution over the points in a low-dimensional map is constructed, and the Kullback-Leibler divergence between the two distributions is minimised with respect to the location of the points in the map. In practice, t-sne represents an algorithm for dimensionality reduction, acting in a more sophisticated manner to a simpler linear idea of projection sub-space discovery as the familiar standard Principal Component Analysis (PCA). Via the t-sne it is then possible to observe that there exists sub-spaces of the feature space in which discriminatory power exists between sick and health patients under the proposed IMF and BLIMF EMD stochastic feature embeddigns, see reults in Fig 12. It is clear that the t-sne shows that both IMFs and BLIMFs appear to separate the two classes of patients, as a result one may expect strong classification performance when using these features. Furthermore, the BLIMFs appear to show better separation than the IMFs. This is due to the fact that, by modelling the frequency domain rather than the time domain, fast changes characterising the formant structure of sick patents are better captured. Note that we provided plots for the female case. Equivalent results were found in the case of the male and not reported for space reasons.

**Fig 13:**
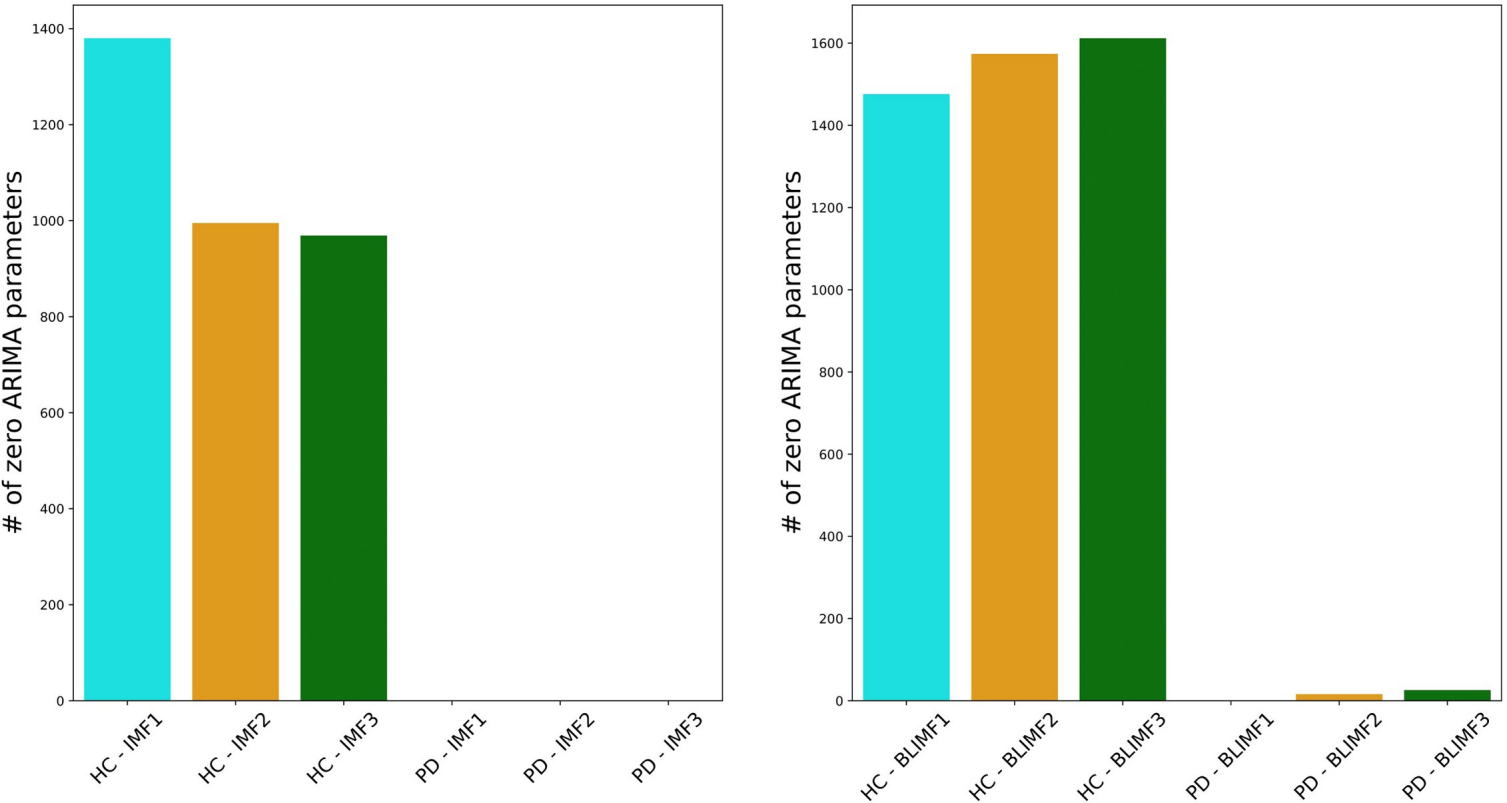
Barplots presenting the number of zero ARIMA parameters fit on the mini-batches for the female case. The left panel refer to the case of the first three IMFs (used in the system model classification and presented in the sections below) split according to healthy (HC) and sick (PD) patients. The right panel presents an equivalent plot referring to the case of he first three BLIMFs, (used in the system model classification and presented in the sections below) split according to healthy (HC) and sick (PD) patients.

We provide a second plot describing the model order complexity of the ARIMA models used to obtain the embedding for the two considered basis function methods: IMF and BLIMFs. Fig 13 presents two panels where the x-axis shows the basis functions (three for both IMFs and BLIMFs) split according to healthy and sick patients for the female case. The y-axis indicates the difference in total model order complexity of the best fitting ARIMA model (total of AR+I+MA coefficients) subtracted from the largest model order considered. Thereby, representing the difference in model order parsimony between models on different features (IMFs or BLIMFs for healthy vs sick patients). If this difference is large, then the complexity of the underlying fitted signal (i.e., the IMFs or the BLIMFs) is more parsimonious, requiring fewer parameters in the ARIMA model to achieve an accurate fit. Indeed, using fewer parameters in a specific segment explains that less autocorrelation is present across each observation. We claim that when Parkinson’s is present, then a much higher autocorrelation will be present in the formants; therefore, many more parameters are required for an efficient fit. By observing the left panel for the IMFs, the bases for the sick patients have no parameters equal to zero, meaning that they have a higher complexity, also signalling a slower autocorrelation decay in the speech features for sick patients vs healthy. This is due to the fact that there are different rates of change in their formant structure due to the presence of Parkinson’s disease and the manifestation of ataxic speech disorder symptoms that arises. For the IMFs related to the healthy participant instead, the number of zero parameters is relatively reduced across the first three IMFs. Since we are considering the female case, the first and the second IMFs are capturing the great majority of the formants; therefore, these two components, particularly the first one, tend to carry more energy content. In comparison to the sick patients, such bases require a significantly lower number of parameters to be fit accurately, supporting our conjecture and the presented results in the sections below.

The right panel shows results for the BLIMFs instead. By looking at the case of the bases for the sick patients, as for the IMFs, a meagre number of zero parameters are found, showing evidence of a more complex structure due to the presence of the disease. In the case of healthy patients, all three BLIMFs appear to have a high number of zero parameters hence providing evidence for a less complex structure compared to the IMFs. The reason behind this is that the third system model partitions the IMFs according to an optimal partition based on the IFs. This clearly shows that such a method captures the frequency content more efficiently since the energy content is split across the three bases more uniformly. In such a way, a better characterisation of each formant can be achieved and, by this mean, a better classification between sick and healthy patients will be achieved. This will be shown in the following subsections.

#### 7.4.3 Model comparisons

Tables 3–6 shows results by gender with achieved accuracy scores by benchmark and proposed models. The accuracy is defined as the sum of the true positive and true negative detected examples over the sum of true positive, true negative, false positive and false negative. Each table is split according to healthy and sick patients, ordered by their UPDRS score. In the female case, most of the patients are healthy; for the sick patients, there are only two stages, being identified as “0” and “1”. In the male case, instead, there are only two healthy patients, while a great deal are instead sick patients. The UPDRS scores range between “0” and “3”. The analysis has been conducted for male and female speakers separately because it is widely known that formants differ significantly between genders, with female formants typically lying at higher frequencies than males. Therefore, any classification or inference procedure tackling speech analysis should consider gender and not pollute the classifier with resonant frequencies that are inaccurately detected since they belong to the other gender class.

**Table 3:** Accuracy performance results of the benchlark female patients. Accuracy is computed as $\frac{\text{TP}+\text{TN}}{\text{TP}+\text{TN}+\text{FP}+\text{FN}}$. The columns show: the UPDRS score (marked as NaN in the case of healthy patients), the benchmark measures performances, corresponding to the MFCCs, MGDCCs, Jitter(%): frequency perturbation, Shimmer (dB): amplitude perturbation, APQ (%): amplitude perturbation quotient, PPQ (%): pitch perturbation quotient, RAP (%): relative average perturbation, CPP mean: mean of cepstral peak prominence corresponding to the mean of voice quality perturbation and CPP s.d.: variation in the cepstral peak prominence corresponding to variation in voice quality perturbation, as given in [29]. Note that the used classifier is the SVM. A cross-validation has been performed for any of the presented results and, therefore, the provided accuracy are the averaged accuracy scores. Configuration for the cross-validation for the benchmark features and the SVM are given in 7.4.

State-of-the-art Benchmark Female Results (Non-EMD Speech Data Based Approaches)—Accuracy													
**Healthy Patients**													
	**Benchmark (SVM)**				**Benchmark—not averaged (SVM)**			**Benchmark—standard (SVM)**					
**UPDRS**	**MFCCs**	**MGDCCs**	**MFCCs + MGDCCs**	**MFCCs**	**MGDCCs**	**MFCCs + MGDCCs**	**Jitter**	**Shimmer**	**APQ**	PPQ	**RAP**	**CPP mean**	**CPP s.d.**
NaN	0.125	0.456	0.500	0.220	0.340	0.510	0.603	0.471	0.566	0.623	0.651	0.552	0.643
NaN	0.221	0.556	0.519	0.410	0.554	0.589	0.236	0.487	0.592	0.389	0.558	0.578	0.661
NaN	0.345	0.665	0.456	0.459	0.311	0.601	0.398	0.410	0.295	0.694	0.230	0.667	0.411
NaN	0.434	0.590	0.435	0.310	0.440	0.489	0.672	0.665	0.661	0.601	0.518	0.531	0.222
NaN	0.367	0.542	0.567	0.398	0.210	0.499	0.411	0.572	0.589	0.671	0.660	0.590	0.559
NaN	0.554	0.453	0.521	0.519	0.558	0.559	0.445	0.456	0.589	0.557	0.365	0.628	0.472
NaN	0.557	0.433	0.567	0.489	0.490	0.601	0.430	0.583	0.418	0.524	0.235	0.254	0.338
NaN	0.515	0.662	0.601	0.550	0.501	0.545	0.414	0.447	0.426	0.513	0.522	0.342	0.567
NaN	0.500	0.345	0.451	0.509	0.567	0.558	0.415	0.672	0.332	0.462	0.557	0.457	0.667
NaN	0.450	0.678	0.401	0.449	0.519	0.589	0.427	0.598	0.492	0.379	0.572	0.243	0.453
NaN	0.650	0.546	0.510	0.551	0.591	0.432	0.453	0.472	0.566	0.331	0.154	0.362	0.647
NaN	0.610	0.634	0.555	0.451	0.553	0.650	0.421	0.463	0.362	0.463	0.624	0.473	0.372
NaN	0.565	0.690	0.501	0.611	0.601	0.678	0.431	0.473	0.245	0.452	0.251	0.531	0.537
NaN	0.656	0.694	0.645	0.611	0.667	0.641	0.451	0.252	0.542	0.253	0.425	0.641	0.654
NaN	0.311	0.601	0.649	0.456	0.489	0.601	0.442	0.425	0.525	0.252	0.7483	0.472	0.472
NaN	0.454	0.550	0.559	0.501	0.551	0.573	0.444	0.593	0.528	0.583	0.572	0.325	0.523
NaN	0.369	0.500	0.590	0.389	0.378	0.456	0.534	0.542	0.251	0.542	0.255	0.542	0.325
NaN	0.328	0.564	0.611	0.456	0.588	0.592	0.429	0.458	0.472	0.325	0.235	0.234	0.252
NaN	0.500	0.445	0.590	0.568	0.588	0.645	0.439	0.545	0.453	0.564	0.234	0.235	0.235
**Sick Patients**													
	**Benchmark (SVM)**				**Benchmark—not averaged (SVM)**			**Benchmark—standard (SVM)**					
**UPDRS**	**MFCCs**	**MGDCCs**	**MFCCs + MGDCCs**	**MFCCs**	**MGDCCs**	**MFCCs + MGDCCs**	**Jitter**	**Shimmer**	**APQ**	PPQ	**RAP**	**CPP mean**	**CPP s.d.**
0	0.256	0.570	0.690	0.358	0.590	0.699	0.557	0.433	0.544	0.746	0.472	0.462	0.340
0	0.543	0.601	**0.701**	0.555	0.619	**0.707**	0.497	0.511	0.566	0.513	0.673	0.374	0.362
1	0.556	0.611	**0.711**	0.501	0.640	0.699	0.558	0.443	0.556	0.462	0.323	0.476	0.453
1	0.343	0.575	**0.702**	0.410	0.595	**0.710**	0.573	0.543	0.435	0.345	0.345	0.647	0.601

**Table 4:** Accuracy performance results of the female patients. Remark that the accuracy is computed as $\frac{\text{TP}+\text{TN}}{\text{TP}+\text{TN}+\text{FP}+\text{FN}}$. The columns show: the UPDRS score (marked as NaN in the case of healthy patients), the SM1, SM2 and SM3 performances obtained with a GLRT. As outlined, the first three bases have been considered for SM2 and SM3. Furthermore, note that a cross-validation has been performed for any of the presented results and, therefore, the provided accuracy are the averaged accuracy scores. Configuration for the cross-validation for SM1, SM2, SM3 and the GLRT in 7.2. Afterwards, the results of the IMFs-MFCCs and BLIMFs-MFCCs are provided. The considered bases are the same of the SM2 and SM3. Configuration for the SVM run corresponds to the same of [29] given in 7.4.

Female Results (EMD Speech Data Based Approaches)—Accuracy																
**Healthy Patients**																
**UPDRS**	**SM1 (GLRT)**	**SM2 (GLRT)**			**SM2 (GLRT)**			**SM3 (GLRT)**			**IMFs-MFCCs (SVM)**			**BLIMFs-MFCCs (SVM)**		
	** $\tilde{s}\left(t\right)$ **	**γ**_**1**_(**t**)	**γ**_**2**_(**t**)	**γ**_**3**_(**t**)	$\gamma_{1}^{\text{s}}\left(t\right)$	$\gamma_{2}^{\text{s}}\left(t\right)$	$\gamma_{3}^{\text{s}}\left(t\right)$	$\gamma_{1}^{\left(\text{BL}\right)}\left(t\right)$	$\gamma_{2}^{\left(\text{BL}\right)}\left(t\right)$	$\gamma_{3}^{\left(\text{BL}\right)}\left(t\right)$	IMF1-MFCCs	IMF2-MFCCs	IMF3-MFCCs	BLIMF1-MFCCs	BLIMF2-MFCCs	BLIMF3-MFCCs
NaN	0.427	0.503	0.485	0.505	0.490	0.560	0.345	0.250	0.056	0.091	0.500	0.450	0.320	0.340	0.300	0.210
NaN	0.440	0.493	0.493	0.494	0.510	0.560	0.610	0.260	0.082	0.139	0.430	0.501	0.420	0.230	0/289	0.231
NaN	0.427	0.490	0.495	0.492	0.501	0.492	0.345	0.299	0.147	0.188	0.350	0.221	0.467	0.124	0.345	0.123
NaN	0.430	0.475	0.497	0.501	0.510	0.310	0.444	0.280	0.093	0.115	0.113	0.114	0.189	0.301	0.120	0.115
NaN	0.411	0.484	0.490	0.486	0.601	0.590	0.518	0.289	0.074	0.076	0.341	0.342	0. 352	0.229	0.221	0.311
NaN	0.445	0.483	0.478	0.495	0.345	0.401	0.528	0.253	0.075	0.127	0.123	0.345	0.429	0.322	0.201	0.122
NaN	0.430	0.482	0.504	0.491	0.450	0.411	0.338	0.275	0.089	0.126	0.210	0.291	0.283	0.111	0.124	0.098
NaN	0.414	0.470	0.488	0.504	0.500	0.341	0.558	0.283	0.070	0.125	0.420	0.368	0.274	0.462	0.511	0.211
NaN	0.415	0.513	0.503	0.505	0.510	0.469	0.435	0.301	0.093	0.121	0.201	0.308	0.113	0.345	0.239	0.216
NaN	0.427	0.484	0.482	0.491	0.439	0.543	0.445	0.248	0.051	0.098	0.489	0.478	0.419	0.398	0.334	0.299
NaN	0.453	0.470	0.491	0.501	0.365	0.445	0.556	0.295	0.077	0.123	0.321	0.352	0.329	0.381	0.201	0.087
NaN	0.421	0.488	0.506	0.517	0.325	0.590	0.589	0.288	0.065	0.095	0.245	0.427	0.421	0.328	0.351	0.112
NaN	0.431	0.469	0.465	0.487	0.549	0.515	0.595	0.282	0.088	0.127	0.431	0.470	0.498	0.275	0.232	0.162
NaN	0.451	0.470	0.498	0.508	0.456	0.601	0.598	0.275	0.093	0.112	0.210	0.321	0.413	0.510	0.231	0.111
NaN	0.442	0.478	0.506	0.483	0.567	0.551	0.510	0.243	0.039	0.058	0.481	0.467	0.435	0.319	0.341	0.214
NaN	0.444	0.471	0.501	0.508	0.434	0.412	0.557	0.301	0.066	0.096	0.324	0.529	0.461	0.254	0.365	0.216
NaN	0.450	0.507	0.490	0.482	0.552	0.587	0.432	0.247	0.074	0.076	0.362	0.312	0.234	0.341	0.312	0.411
NaN	0.429	0.458	0.486	0.504	0.531	0.456	0.564	0.282	0.080	0.094	0.503	0.502	0.556	0.231	0.221	0.101
NaN	0.439	0.509	0.487	0.505	0.598	0.539	0.520	0.345	0.030	0.123	0.231	0.456	0.561	0.113	0.321	0.235
**Sick Patients**																
**UPDRS**	**SM1 (GLRT)**	**SM2 (GLRT)**			**SM2 (GLRT)**			**SM3 (GLRT)**			**IMFs-MFCCs (SVM)**			**BLIMFs-MFCCs (SVM)**		
	$\tilde{s}\left(t\right)$	**γ**_**1**_(**t**)	**γ**_**2**_(**t**)	**γ**_**3**_(**t**)	$\gamma_{1}^{\text{s}}\left(t\right)$	$\gamma_{2}^{\text{s}}\left(t\right)$	$\gamma_{3}^{\text{s}}\left(t\right)$	$\gamma_{1}^{\left(\text{BL}\right)}\left(t\right)$	$\gamma_{2}^{\left(\text{BL}\right)}\left(t\right)$	$\gamma_{3}^{\left(\text{BL}\right)}\left(t\right)$	IMF1-MFCCs	IMF2-MFCCs	IMF3-MFCCs	BLIMF1-MFCCs	BLIMF2-MFCCs	BLIMF3-MFCCs
0	0.557	0.533	0.508	0.510	**0.701**	**0.705**	0.690	**0.736**	**0.995**	**0.895**	**0.750**	**0.732**	0.698	**0.853**	**0.867**	**0.701**
0	0.497	0.527	0.500	0.513	0.652	0.690	**0.711**	**0.811**	**0.959**	**0.888**	**0.747**	**0.711**	**0.716**	**0.800**	**0.830**	**0.715**
1	0.558	0.535	0.482	0.507	**0.710**	**0.701**	**0.700**	**0.710**	**0.935**	**0.882**	**0.743**	**0.801**	0.661	**0.745**	**0.860**	**0.878**
1	0.573	0.520	0.510	0.491	**0.783**	**0.711**	0.601	**0.790**	**0.950**	**0.899**	**0.742**	**0.711**	**0.706**	**0.748**	**0.880**	**0.872**

**Table 5:** Accuracy performance results of the male patients. Remark that the accuracy is computed as $\frac{\text{TP}+\text{TN}}{\text{TP}+\text{TN}+\text{FP}+\text{FN}}$. The columns show: the UPDRS score (marked as NaN in the case of healthy patients), the benchmark measures performances, corresponding to the MFCCs, MGDCCs, Jitter(%): frequency perturbation, Shimmer (dB): amplitude perturbation, APQ (%): amplitude perturbation quotient, PPQ (%): pitch perturbation quotient, RAP (%): relative average perturbation, CPP mean: mean of cepstral peak prominence corresponding to the mean of voice quality perturbation and CPP s.d.: variation in the cepstral peak prominence corresponding to variation in voice quality perturbation, as given in [29]. Note that the used classifier is the SVM. A cross-validation has been performed for any of the presented results and, therefore, the provided accuracy are the averaged accuracy scores. Configuration for the cross-validation for the benchmark features and the SVM are given in 7.4.

State-of-the-art Benchmark Male Results (Non-EMD Speech Data Based Approaches)—Accuracy													
Healthy Patients													
UPDRS	Benchmark (SVM)				Benchmark—not averaged (SVM)				Benchmark—standard (SVM)				
	MFCCs	MGDCCs	MFCCs + MGDCCs	MFCCs	MGDCCs	MFCCs + MGDCCs	Jitter	Shimmer	APQ	PPQ	RAP	CPP mean	CPP s.d.
NaN	0.410	0.515	0.519	0.500	0.511	0.558	0.379	0.583	0.264	0.327	0.453	0.463	0.472
NaN	0.643	0.590	0.571	0.519	0.576	0.598	0.379	0.463	0.527	0.274	0.463	0.665	0.655
Sick Patients																
UPDRS	Benchmark (SVM)				Benchmark—not averaged (SVM)				Benchmark—standard (SVM)				
	MFCCs	MGDCCs	MFCCs + MGDCCs	MFCCs	MGDCCs	MFCCs + MGDCCs	Jitter	Shimmer	APQ	PPQ	RAP	CPP mean	CPP s.d.
0	0.520	0.650	0.611	0.551	0.656	0.678	0.627	0.573	0.647	0.445	0.465	0.653	0.365
0	0.555	0.600	0.619	0.458	0.623	0.674	0.602	0.553	0.453	0.543	0.637	0.455	0.446
0	0.390	0.588	0.690	0.553	0.598	0.593	0.535	0.511	0.453	0.554	0.553	0.437	0.372
0	0.430	0.590	0.699	0.441	0.489	0.563	0.635	0.563	0.477	0.564	0.574	0.463	0.477
0	0.551	0.500	0.652	0.428	0.649	0.693	0.610	0.674	0.342	0.245	0.572	0.425	0.254
1	0.439	0.595	0.702	0.469	0.532	0.564	0.456	0.467	0.578	0.656	0.564	0.564	0.463
1	0.312	0.610	0.712	0.654	0.689	0.709	0.626	0.465	0.553	0.562	0.465	0.463	0.698
1	0.235	0.645	0.705	0.613	0.601	0.731	0.616	0.505	0.676	0.698	0.687	0.556	0.699
1	0.611	0.650	0.675	0.689	0.673	0.678	0.595	0.666	0.699	0.689	0.687	0.685	0.563
2	0.387	0.611	0.718	0.445	0.562	0.699	0.628	0.668	0.668	0.635	0.678	0.675	0.689
2	0.654	0.674	0.731	0.510	0.661	0.722	0.581	0.677	0.678	0.675	0.698	0.698	0.678
3	0.442	0.659	0.750	0.567	0.698	0.719	0.678	0.659	0.665	0.678	0.699	0.688	0.667

**Table 6:** Accuracy performance results of the male patients. Remark that the accuracy is computed as $\frac{\text{TP}+\text{TN}}{\text{TP}+\text{TN}+\text{FP}+\text{FN}}$. The columns show: the UPDRS score (marked as NaN in the case of healthy patients), the SM1, SM2 and SM3 performances obtained with a GLRT. As outlined, the first three bases have been considered for SM2 and SM3. Furthermore, note that a cross-validation has been performed for any of the presented results and, therefore, the provided accuracy are the averaged accuracy scores. Configuration for the cross-validation for SM1, SM2, SM3 and the GLRT in 7.2. Afterwards, the results of the IMFs-MFCCs and BLIMFs-MFCCs are provided. The considered bases are the same of the SM2 and SM3. Configuration for the SVM run corresponds to the same of [29] given in 7.4.

Male Results (EMD Speech Data Based Approaches)—Accuracy																
**Healthy Patients**																
UPDRS	**SM1 (GLRT)**	**SM2 (GLRT)**			**SM2 (GLRT)**			**SM3 (GLRT)**			**IMFs-MFCCs (SVM)**			**BLIMFs-MFCCs (SVM)**		
	$\tilde{s}\left(t\right)$	**γ**_**1**_(**t**)	**γ**_**2**_(**t**)	**γ**_**3**_(**t**)	$\gamma_{1}^{\text{s}}\left(t\right)$	$\gamma_{2}^{\text{s}}\left(t\right)$	$\gamma_{3}^{\text{s}}\left(t\right)$	$\gamma_{1}^{\left(\text{BL}\right)}\left(t\right)$	$\gamma_{2}^{\left(\text{BL}\right)}\left(t\right)$	$\gamma_{3}^{\left(\text{BL}\right)}\left(t\right)$ $\gamma_{3}^{\left(\text{BL}\right)}\left(t\right)$	IMF1-MFCCs	IMF2-MFCCs	IMF3-MFCCs	BLIMF1-MFCCs	BLIMF2-MFCCs	BLIMF3-MFCCs
NaN	0.379	0.513	0.445	0.463	0.500	0.567	0.490	0.225	0.235	0.059	0.543	0.392	0.321	0.252	0.201	0.211
NaN	0.379	0.488	0.449	0.462	0.531	0.450	0.441	0.225	0.250	0.026	0.445	0.379	0.500	0.201	0.210	0.098
**Sick Patients**																
UPDRS	**SM1 (GLRT)**	**SM2 (GLRT)**			**SM2 (GLRT)**			**SM3 (GLRT)**			**IMFs-MFCCs (SVM)**			**BLIMFs-MFCCs (SVM)**		
	$\tilde{s}\left(t\right)$	**γ**_**1**_(**t**)	**γ**_**2**_(**t**)	**γ**_**3**_(**t**)	$\gamma_{1}^{\text{s}}\left(t\right)$	$\gamma_{2}^{\text{s}}\left(t\right)$	$\gamma_{3}^{\text{s}}\left(t\right)$	$\gamma_{1}^{\left(\text{BL}\right)}\left(t\right)$	$\gamma_{2}^{\left(\text{BL}\right)}\left(t\right)$	$\gamma_{3}^{\left(\text{BL}\right)}\left(t\right)$ $\gamma_{3}^{\left(\text{BL}\right)}\left(t\right)$	IMF1-MFCCs	IMF2-MFCCs	IMF3-MFCCs	BLIMF1-MFCCs	BLIMF2-MFCCs	BLIMF3-MFCCs
0	0.627	0.499	0.528	0.521	0.690	0.611	**0.710**	**0.787**	**0.727**	**0.911**	0.678	**0.701**	**0.734**	**0.715**	**0.823**	**0.868**
0	0.602	0.493	0.537	0.534	0.601	0.699	**0.722**	**0.865**	**0.729**	**0.911**	**0.700**	0.699	**0.734**	**0.782**	**0.810**	**0.888**
0	0.597	0.502	0.527	0.549	0.610	**0.729**	**0.730**	**0.764**	**0.741**	**0.920**	0.667	0.689	**0.793**	**0.798**	**0.816**	**0.849**
0	0.635	0.480	0.522	0.523	0.673	**0.719**	**0.710**	**0.729**	**0.724**	**0.878**	**0.711**	**0.763**	**0.793**	0.699	**0.802**	**0.899**
0	0.610	0.485	0.548	0.549	**0.715**	0.690	**0.721**	**0.763**	**0.722**	**0.916**	0.672	**0.717**	**0.798**	**0.802**	**0.810**	**0.899**
1	0.615	0.496	0.551	0.522	**0.700**	**0.711**	**0.735**	**0.821**	**0.764**	**0.954**	0.659	**0.748**	**0.762**	**0.791**	**0.784**	**0.834**
1	0.626	0.502	0.548	0.545	**0.709**	**0.705**	**0.721**	**0.845**	**0.762**	**0.947**	0.698	**0.710**	**0.787**	**0.785**	**0.810**	**0.873**
1	0.616	0.505	0.546	0.575	**0.711**	0.610	**0.721**	**0.745**	0.690	**0.835**	0.699	0.689	**0.786**	**0.788**	**0.801**	**0.890**
1	0.595	0.510	0.534	0.534	0.687	**0.722**	**0.720**	**0.780**	**0.731**	**0.926**	**0.706**	**0.713**	**0.798**	**0.716**	**0.819**	**0.878**
2	0.628	0.485	0.505	0.533	**0.733**	**0.741**	**0.745**	**0.881**	**0.760**	**0.923**	0.645	**0.799**	**0.811**	**0.710**	**0.810**	**0.898**
2	0.581	0.492	0.543	0.550	**0.721**	**0.730**	**0.727**	**0.888**	**0.899**	**0.910**	0.689	**0.785**	**0.798**	**0.781**	**0.867**	**0.901**
3	0.634	0.489	0.537	0.638	**0.720**	**0.711**	**0.749**	**0.899**	**0.950**	**0.949**	0.668	**0.787**	**0.795**	**0.733**	**0.890**	**0.910**

We compare EMD-GP proposed models to reference benchmark features for speech analysis previously used in ataxic speech detection for Parkinson’s disease [29, 79]. Each model is introduced in Table 1 and in subsection 6.1. Note that, before extracting any of the state-of-the-art features, we pre-emphasise and Hamming-windowed $\tilde{s}\left(t\right)$ to avoid issues of aliasing in discrete sample MFCCs or MGDCCs representations. Each speech signal is subject to a 0.97 pre-emphasis factor. It is then segmented into frames of 25ms with 50% overlap, meaning, for a sampling frequency *f*_*s*_ = 44.1 kHz, that the total number of samples in each frame is *N*_*s*_ = 1102.5. We further extract MFCCs from the IMFs and BLIMFs, by following the approach provided in [17]. Equivalent treatments are applied to the bases before computing the IMFs-MFCCs and the BLIMFs-MFCCs.

Results of state-of-the-art features are given in Tables 3 and 5. As [29], we extracted the coefficient sets on frames of the original speech signals and then averaged them across the considered frames, resulting in 12 averaged MFCCs and 11 averaged MGDCCs for each speaker. We further compute the non-averaged and individual coefficients cases, performing classifications with only the MFCCs and the MGDCCs. Similarly, the benchmark set proposed in [29] is used. We reproduced equivalent classification procedures with a kernel-based SVM and a cross-validation procedure with 10 folds. Regarding averaging the coefficients, [29] claimed that such an operation trades accuracy for computation speed. However, most of the discriminant power lies in the abnormal changes of the various speech frames, and the averaging would smooth the energy content of the derived coefficients. The obtained low performances of these features in our work support precisely such a statement, along with the fact that the most significant problem of these decomposition techniques, i.e. the MFCCs or the MGCCs, is their required stationarity assumption, which is rarely achieved if not in optimal recording environments with silence and non-reverberation conditions. This is unattainable in standard medical facilities or with voice recordings over wireless devices such as mobile phones. A further discussion of these challenges can be found in [17]. Amongst the various benchmark considered, no features achieved an accuracy superior to 70% accuracy, limiting their use in these medical diagnostic areas. These features also rely on stationary frequency transformations which are not achieved in these practices promoting telemedicine.

The results of the EMD-GP structures are given in Tables 4 and 6, for females and males, respectively. SM2 and SM3 results are provided for the first three IMFs and BLIMFs. Results for the residual tendency and the rest of the IMFs are not presented. They have been tested, and no better results have been achieved. As highlighted above and provided in [17] (and reference within), the first three IMFs capture most of the formants structure acting as a human speech fingerprint representing a powerful discriminant tool for the characterisation of ataxic speech. Another critical point is that the original IMFs *γ*_1_(*t*), *γ*_2_(*t*), *γ*_3_(*t*) often carry a great deal of noise. Therefore, a median filter has been applied, providing a smoother version of such bases denoted as $\gamma_{1}^{s}\left(t\right),\gamma_{2}^{s}\left(t\right),\gamma_{3}^{s}\left(t\right)$.

Once the EMD is computed, the IFs have been extracted. The following step is applying the cross-entropy method to compute the BLIMFs. We select the first three IFs for this step, i.e. *ω*_1_(*t*), *ω*_2_(*t*), *ω*_3_(*t*), since the great deal of formants will be described by them. In the configuration of the CEM, we selected *M* = 3 and *D* = 5, *ρ* = 0.2, *β* = 0.6, *S* = 100, *N*_*ω*_ = 100, *N*_*τ*_ = 100 and a maximum number of CEM iteration was equal to 100. Alternatives have been considered, but similar results were obtained, and, therefore, we select the minimum number to obtain a low computational cost. Once performed, the CEM provides a set of grid points, i.e. *ω*_*m*_ and *s*_*m*,*d*_ for *m* = 1, …, *M*, *d* = 1, …, *D* which partition the time-frequency plane. Then the BLIMFs are derived as given in Eq 12 and the GLRT test is applied as for SM2.

A further point made in [17] is that the MFCCs can be more efficiently exploited when applied to the IMFs bases, which indeed capture formant structure. Moreover, the MFCCs rely on Fourier-type transformations which require stationarity of the underlying signal. Hence, deriving such coefficients on the IMFs, which carry minor levels of non-stationarity compared to the raw signals, is highly beneficial. We introduce a new feature type by applying the MFCCs to the BLIMFs. MGDCCs were also applied on such bases, but results are not shown since they are not optimally performing.

## 8 Discussion and conclusions

We start by focusing on the benchmark female accuracy scores provided in Table 3. Across the state-of-the-art features, the MFCCs combined with the MGDCCs were more reliable than using the individual sets of MFCC or MGDCCs separately. The combined benchmarks of MFCC+MGDCCs represent the standard to beat using the EMD-GP methods. These results produced an accuracy result around 70%. This is the case in both the averaged and non-averaged coefficients settings, suggesting that the technique undertaken in [29] provides an effective solution since saving part of the computational cost required for an SVM using all the coefficients. Equivalent results are achieved in Table 5 in the male case, showing the maximum accuracy result of 75%. The main issues encountered with these features include the following challenges. Firstly, there is a requirement for stationarity of the underlying signal, which is rarely respected, especially when the speech signal is not recorded in an ideal noise-free environment. In standard medical settings, there is significant background noise, there are non-ideal microphones used in phones or mobile devices. Secondly, in the case of averaging the coefficients, most of the discriminant power carried by the frames describing the individual biometric formant structures will be polluted with the average operation. The final objective is indeed identifying which time-frequency regions, by gender, can discriminate ataxic speech. This is a delicate exercise per se, which should always take into account these observations and carefully consider the possibility of contamination of the classifier when reduction of complexity is in favour of the employed method. Furthermore, when it comes to health diagnostic, an accuracy score of 70% will not be considered since it is highly risky and therefore more powerful solutions need to be considered.

Next, by looking at Tables 4 and 6, it was demonstrated that when using a GLRT test, fitting a GP model directly to the speech signal is ineffective since the covariance function (GP kernel) function is not sufficiently flexible to capture the structure required to discriminate ataxic speech features. This is true even with the data-adaptive Fisher kernel structure; it does not provide any significant results in both sets of analysis, i.e. for males or females. Indeed, the formant behaviour of the underlying signals carries a very complex structure affected by fast changes, which are not only due to the presence or absence of ataxic speech.

Therefore, such time-frequency fast variant modes require a refined modelling methodology, which, in this work, is represented by the stochastic embedding of the IMFs and the BLIMFs under the EMD-GP structures proposed. The next step is indeed to consider SM2 with the first three IMFs. These bases still do not show acceptable performances. It is often the case that the IMFs capture most of the data non-stationarity; therefore, their power in modelling fast changes may be reduced. However, by applying a median filter to $\gamma_{1}^{s}\left(t\right),\gamma_{2}^{s}\left(t\right),\gamma_{3}^{s}\left(t\right)$, better performances are obtained in this robust version. In the female case, the maximum achieved accuracy corresponds to 78%, while in the male case to 74%. What is important to notice at this stage is that in the former case, most of the discriminant power lies in the highest IMFs, i.e. *γ*_1_(*t*) and *γ*_2_(*t*), since females tend to have higher formants which are detected by higher frequency content of the EMD decomposition. In the male case, the third IMF shows more patients with the highest accuracy levels. Indeed, male voices tend to have formants at lower frequencies detected by $\gamma_{3}^{s}\left(t\right)$. This is particularly meaningful since it reflects the standard formants structure of female and male voices in general and provides useful interpretation to further develop such a modelling idea.

The best performing model came from the EMD-GP model structure based on using the first three BLIMFs defined previously and denoted by SM3. This outperformed all benchmarks and all other competitor models. The CEM has been applied to the first three IFs with configuration explained at the beginning of this section 7.4. The performances of this system model are outstanding compared to any other model. In the female case, $\gamma_{2}^{\left(\text{BL}\right)}\left(t\right)$ achieves levels of accuracy greater than 90% for any patient with any UPDRS score. $\gamma_{3}^{\left(\text{BL}\right)}\left(t\right)$ also provides high performances always greater than 88%, while $\gamma_{1}^{\left(\text{BL}\right)}\left(t\right)$ achieves accuracy scores of 73% at least. With the use of the CEM, the discriminatory power is shifted towards the second and third BLIMFs, rather than in IMF1 and IMF2, with significant performance gains achieved. This shows that the CEM can isolate more stationary basis functions characterised by the same frequency content and provide more powerful discrimination. As for the female case, all the BLIMFs for all patients in the male case provide high accuracy score levels. Highest performances are given by the third BLIMF, which achieves 90% for almost every patient.

While in the female case, the second BLIMF shows the best performances, in this case is $\gamma_{3}^{\left(\text{BL}\right)}\left(t\right)$ that carries most of the discriminatory power. This again reflects how males have lower formants than females and therefore detected by the third BLIMF. The second and the first BLIMFs well perform and provide high levels of accuracy. Furthermore, with the increase of the UPDRS score and hence the Parkinson’s stage, the accuracy increases across all the three basis functions, which suggests the BLIMFs well detect the progression of the disease.

A further set of features was provided in both Tables 4 and 6. This is based on [17] promoting the extraction of MFCCs on the IMFs and, given the novelty of this work, on the BLIMFs. As discussed, this kind of coefficient’s main issue is the stationarity requirement for the underlying signal. Using the IMFs and BLIMFs rather than the raw data allows, in both cases, i.e. males and females, a significant increase in the performances. Indeed, compared to the state-of-the-art provided by [29], these features show strong discrimination power with some combinations for the male case achieving 90% of accuracy. This proves a clear advantage in using the IMFs bases or the BLIMFs rather than the original signals. The advantage of such methods also lies in the interpretation associated with the obtained results. The IMFs-MFCCs coefficients better detecting Parkinson’s disease in the female case correspond to the ones of the first and second IMFs since capturing the highest formants of female voices hence finding discrimination power. In the case of male voices, a great deal of power lies instead in the second and third IMFs, revealing indeed the presence of formants lying at lower frequencies. We firmly believe that capturing the formant structure with such decomposition proposed methods will be the keystone to differentiate amongst the different types of dysarthria.

Two significant contributions were provided in this manuscript. The first was methodological in nature. We developed a novel technique for the stochastic embedding of the Empirical Mode Decomposition. This is lacking in the literature and introduces the definition of stochastic Multi-Kernel EMD by allowing for more robust solutions in classification or forecasting models based on non-stationary signal decomposition methods. As highlighted, two different stochastic EMD-GP embeddings have been presented. The first directly utilises the original IMFs in a GP compositional structure, while the second relies on an optimal cross-entropy-based procedure used to define band-limited IMFs (BLIMFs), which produce distributions more consistent with stationarity properties, making the fitting of GP models in the EMD-GP based BLIMF stochastic embedding more reliable than that obtained using only the original EMD IMFs. The selection of the optimal partitions to characterise the BLIMFs utilised a novel use of the cross entropy method based on importance sampling distribution to derive the optimal time-frequency partition employed for defining the BLIMFs. The introduction of the BLIMFs in the literature allows for probabilistic statements directly on the frequency domain, which has been a significant challenge in the literature for decades.

The second significant contribution produced was an essential demonstration of the utility of the stochastic embedding models for the EMD-GP frameworks, using both IMFs and BLIMFs. This allowed for the formulation of an ASR-SD system relying on such bases. It was shown that the stochastic EMD-GP embedding structures could be used in a GLRT-based inference testing procedure for speech signals to detect ataxic speech features. This is a critical task to solve when detecting the possibility of Parkinson’s disease in patients from those who do not display standard ataxic speech features. It was demonstrated that using the BLIMFs and GP stochastic embedding structures produced accuracies for the detection of ataxic speech in Parkinson’s patients with far greater accuracy than current state-of-the-art methods using SVMs and also outperformed standard GP models that did not utilise the EMD frameworks. This has been the case even when the adopted state-of-the-art kernel designs are based on a generative embedding framework for time-series kernels based on Fisher kernels. We furthermore proved the relevance of IMFs and BLIMFs by characterising novel features based on the fact that the application of the MFCCs on the raw data would always suffer from the stationarity requirements of these methodologies. Hence, the need for the proposed decomposition techniques further provides a relevant interpretation. We believe that the proposed EMD-GP frameworks hold great potential for developing other speech disorder analyses and detection of symptoms consistent with different neurological disorders, especially accurately when utilised in real-world recording environments using mobile phones in open doctors’ office environments or hospitals, where background noises can be significant. We demonstrated that even in such recording settings, it was still possible to diagnose ataxic speech accurately. This shows a substantial improvement over the current state-of-the-art methods we implemented compared to the real data case study.

## Supporting information

S1 File (PDF)
