# Supplementary material for: Ataxic speech disorders and Parkinson’s disease diagnostics via stochastic embedding of empirical mode decomposition
Source: PLoS One. 2023 Apr 26;18(4):e0284667. doi: 10.1371/journal.pone.0284667 (PMC10132693; doi:10.1371/journal.pone.0284667)
Supplement: S1 File — (PDF) [file pone.0284667.s001.pdf]

Supporting Information for Ataxic Speech Disorders and Parkinson's Disease Diagnostics via Stochastic Embedding of Empirical Mode Decomposition

Marta Campi, Gareth W. Peters, Dorota Toczydlowska

1 CERIAH team, Institut de L'Audition, Institut Pasteur, Paris, France
2 Department of Statistics & Applied Probability, University of California Santa Barbara (UCSB), Santa Barbara, California
3 School of Mathematics and Physical Science, University of Technology Sydney, Sydney, Australia

¤Current Address: CERIAH team, Institut de L'Audition, Institut Pasteur, Paris, France
marta.campi.11@gmail.com

Abstract

In this Supplementary Information document, we present extra material required for the paper "Ataxic Speech Disorders and Parkinson's Disease Diagnostics via Stochastic Embedding of Empirical Mode Decomposition". The organization of the Supplementary Information is presented in the introduction and then different sections are presented.

Author summary

Dr. Marta Campi. Dr. Marta Campi received her B.Sc in Mathematical Statistics and Data Processing (SMID) at the Department of Mathematics of the School of Mathematical, Physical and Natural Sciences at the University of Genoa, Italy. She then received an MSc in Financial Econometrics taught jointly between the Department of Economics and Essex Business School at University of Essex, Colchester, UK. After that, she took an MRes in Financial Computing from the Computer Science Department at University College London (UCL), London, (UK) followed by an MPhil from the Statistical Science Department at UCL. She is currently a Ph.D. student at the Statistical Science Department of UCL. During her Ph.D. she has been invited at the Institute of Statistical Mathematics, Tokyo (Japan) at the Department of Statistical Modeling as research fellow to investigate aspects of speech cyber-security problems. Afterwards, she started to conduct research in the field of speech health diagnostic. She has been awarded her PhD in March 2022. She is currently a PostDoc at the Hearing Institute of Pasteur in Paris, France within the CERIAH team.

Prof. Gareth W. Peters. Prof. Gareth W. Peters is the "Janet and Ian Duncan Endowed Chair Professor in Actuarial Science" and "Chair Professor in Statistics for Risk and Insurance" in the Department of Applied Probability and Statistics in University of California Santa Barbara. Previously, Prof. Peters was the Chair Professor for Statistics in Risk and Insurance in the Department of Actuarial Mathematics and Statistics, in Heriot-Watt University in Edinburgh, where he was also the Director of the Scottish Financial Risk Academy (SFRA). Previously he held tenured positions in the

Department of Statistical Sciences, University College London, UK and the Department of Mathematics and Statistics in University of New South Wales, Sydney, Australia. Prof. Peters was also the Nachdiploma Lecturer in Machine Learning for Risk and Insurance at ETH Zurich in the Risk Laboratory. Prof. Peters has made in excess of 150 international invited presentations, speaker engagements including numerous key note presentations. He has delivered numerous professional training courses to c-suite executive level industry professionals as well as numerous central banks. Prof. Peters has published in excess of 150 peer reviewed articles on risk and insurance modelling, 2 research text books on Operational Risk and Insurance as well as being the editor and contributor to 3 edited text books on spatial statistics and Monte Carlo methods.

Dr. Dorota Toczydlowska . Dr. Dorota Toczydlowska received her B.Sc in Mathematics at the University of Warsaw, Poland. She then received an MSc in Financial Mathematics again at the University of Warsaw, Poland. After that, she took an MRes in Financial Computing from the Computer Science Department at University College London (UCL), London, (UK) followed by an MPhil from the Statistical Science Department at UCL. She then received a PhD at the Statistical Science Department of UCL. Dr. Toczydlowska has been a Postdoctoral Researcher at University of Technology, Sydney for two years.

1 Introduction

In this Supplementary Information document we provide further materials required for the understanding of the main paper. Note that, apart from the code for the three system models implemented, at this Github page `https://github.com/mcampi111`, it is possible to find a repository named “EMD-Stochastic-Embedding-for-PD-Speech” in which further notebooks have been provided for some of the sections below presented.

The document is organised as follow: firstly, we provide evidence of why the Fisher kernel is required in the implementation of this stochastic embedding. Traditional kernel matrices will not be able to fit the speech signals and, therefore, an ad hoc kernel structure is highly needed. This is presented in the first section. Afterwards, further details of the considered dataset are provided. Section 4 presents the steps of the fitting procedure required to extract the best models for the construction of the Fisher scores in the testing procedure. Section 5 presents the steps required to implement the testing procedure and how to implement the Gram Matrices for the GLRT test.

2 Gram Matrices and Covariance Matrices

Fig. 1 shows four different panels. The top panels present two randomly selected segments for the raw data of two male voices with their associated empirical covariance matrices (the bottom plots). Remark that the empirical covariance is computed as $\tilde{s}(t)^{i,\intercal}\tilde{s}(t)^{i}$ for the i-th segment. Each segment is made of 5000 samples corresponding to approximately 0.13 seconds given that the signals were recorded at 44.1 kHz. The top left panel is a segment of a male, healthy patient, while the the top right panel represents a segment of a male, sick patient. Fig. 2, instead, represents two Gram Matrices obtained by using the radial basis function kernel which are evaluated on a uniform grid of points of length 5000 samples (as the covariance matrices). The length scale hyperparameter was set to $l = 0.1$ for the left panel and $l = 2$ for the right panel.

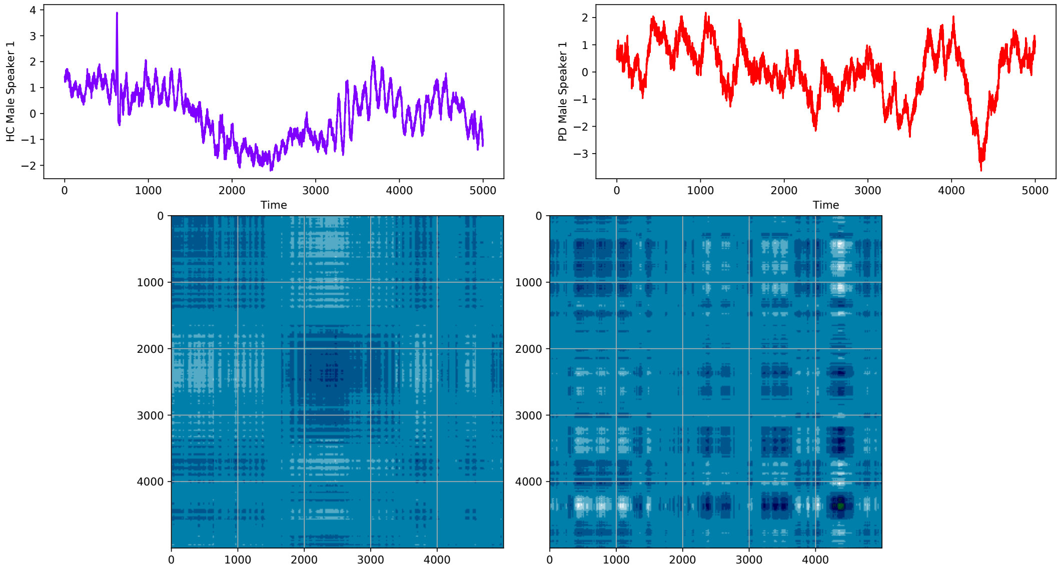

Fig 1. Raw speech segments (top plots) for two male voices wit the associated empirical covariance matrices (bottom plots). In the top panels, the x-axis corresponds to time. This is expressed in terms of number of samples, i.e. 5000 samples which is equivalent to 0.13 seconds for a recording frequency of 441. kHz. The y-axis is the amplitude of the considered speech segments. The bottom panels show the empirical covariance matrices of the above segments. If the segment is denoted as $\tilde{s}(t)^i$, then the empirical covariance matrix is computed as $\tilde{s}(t)^{i,\intercal}\tilde{s}(t)^i$.

The plots for the empirical covariance matrices show that the underlying structures of the original data are not trivial and that any classical stationary kernel as the radial basis function would fail in detecting it efficiently. As a result, the authors decided to employ the data driven kernel known as the Fisher Kernel.

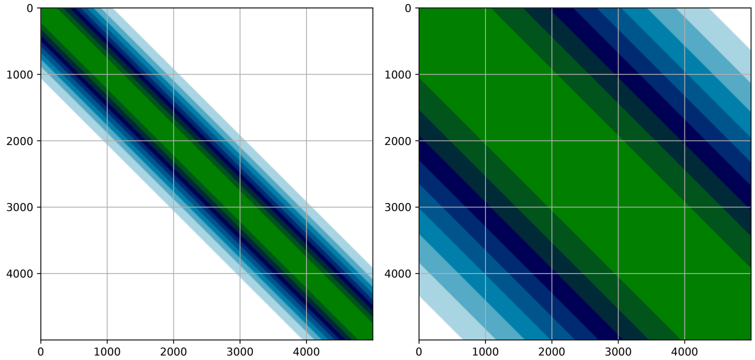

Fig 2. Gram Matrices of the radial basis function kernel evaluated on a uniform grid of points of length 5000 with two hyperparameters for the length scale. The left panel represent a Gram Matrix with $l = 0.1$. The right panel represent a Gram Matrix with $l = 2$.

3 The King's College Dataset

In this section, we provide further information about the King's College Dataset employed for the experiments within the main body of the paper. The Parkinson

participants are labelled according to the following scores: the HYR score with a range between 0 and 5 and then the UPDRS II-5 score and the UPDRS III-18 score.

The HYR score is known as the The Hoehn and Yahr Scale and was firstly published in 1967 (see [1]) and is used to measure how Parkinson's symptoms progress and the level of disability. Stage 0 corresponds to less severe labelled as " No signs of disease", while stage 5 the most severe given as "Needing a wheelchair or bedridden unless assisted". By considering the UPDRS II-5 score, the Parkinson's participants are classified in a range between 0 and 3 at maximum, particularly for the female patients, 2 are at a 0 stage level and 2 are at a 1 stage level. In the case of the sick male patients, 5 male patients are at a 0 stage level, 4 patients at 1 stage level, 2 patients at 2 stage level and 1 patient at a 3 stage level. The top barplots of Fig. 3 represents a summary of the described database. The barplots are separated by gender which is shown on the x-axis. Note that the left barplot provides information about the healthy patients while the right one about sick patients. The bottom barplots represent the number of ill patients split according to their UPDRS II-5 score (which goes from 0 to 3 in the dataset).

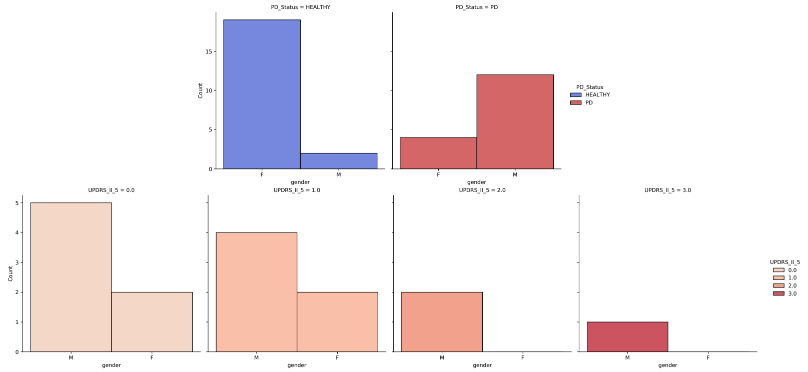

Fig 3. Barplots describing the participants of the considered case study. The upper panel is divided in two separate barplots. The left show the number of healthy participants of the datataset (controls) and the right one shows the number of sick patients. The x-axis is split within both barplots between gender and the y-axis shows the counts of the patients. The lower panel shows four different plots describing the sick patients divided by UPDRS II-5 score. The left barplot shows the sick patients split by gender with UPDRS II-5 score equal to 0. Then, from left to right, equivalent barplots are presented with the UPDRS II-5 score increasing from 0 to 3, which is the maximum assigned score for only one male patient. The x-axis is split between gender and the y-axis shows the count of the patients.

4 The Fitting Procedure for The Estimation Model Phase

In this section, the fitting procedure of the time series models is presented. Consider the female case, for example. Denote the interpolated signals through a cubic spline for a female Parkinsons's voice as $\tilde{s}(t)_1$ and for a healthy female voice as $\tilde{s}(t)_0$, with $t \in [t_0, \dots, t_N]$. Hence, the 0 index refers to a female voice not affected by Parkinson's, while the 1 index refers to a female voice affected by it. An equivalent notation can be considered for a male patient. The original voices are firstly split into segments of length 5000. Therefore, the notation for one segment will become $\tilde{s}(\boldsymbol{t}_i)_0$ and $\tilde{s}(\boldsymbol{t}_i)_1$, where $i = 1, \dots, N_f$ are the indices referring to the segment number for one of the two groups, i.e healthy or Parkinson female patients, respectively, and $\boldsymbol{t}_i$ corresponds to an

input vector belonging to the following mesh

$$\begin{aligned}\boldsymbol{T} &= \left[\boldsymbol{t}_1, \boldsymbol{t}_2, \ldots, \boldsymbol{t}_{N_f}\right] \\ &= \left[\left[t_1, \ldots, t_{5000}\right], \left[t_{5001}, \ldots, t_{10000}\right], \ldots, \left[t_{N-4999}, \ldots, t_N\right]\right]\end{aligned}$$

Note that the same number of segments were randomly selected for the two classes of healthy and sick patients. Select now the segments for the healthy female voice denoted as $\tilde{s}(\boldsymbol{t}_i)_0$, $i = 1, \ldots, N_f$. The goal is to characterise their local structure through a collection of scorings directly depending on the generative model inducing the data generating process of such a speech type, i.e. healthy and female. To achieve this result, one further splits each segment $\tilde{s}(\boldsymbol{t}_i)_0$ into mini-batches of length 100 sample points (corresponding to 2.2 ms). Therefore, one will have $\tilde{s}(\boldsymbol{t}_i^j)_0$ with $j = 1, \ldots, 50$ and $i = 1, \ldots, N_f$. We further redefine the mesh for the input variable set $\boldsymbol{T}$ referring to a segment $\tilde{s}(\boldsymbol{t}_i)_0$ as

$$\begin{aligned}\boldsymbol{t}_i &= [\boldsymbol{t}_i^1, \ldots, \boldsymbol{t}_i^{50}] \\ &= \left[[t_1, \ldots, t_{100}]^i, [t_{101}, \ldots, t_{200}]^i, \ldots, [t_{4901}, \ldots, t_{5000}]^i\right]\end{aligned}$$

for $i = 1, \ldots, N_f$. Note that, for each mini-batch $\tilde{s}(\boldsymbol{t}_i^j)_0$, a set of ARIMA models given in Table 1 will be fit without an intercept. Instead, for each mini-batch $\tilde{s}(\boldsymbol{t}_i^j)_1$, only an ARIMA(3,1,3) with intercept included will be fit. The main reason to do so is that a mini-batch belonging to the sick patients hence $\tilde{s}(\boldsymbol{t}_i^j)_1$ will have a much more complex structure due to faster changes in the speech and, therefore, will require more parameters to be efficiently detected. For a healthy mini-batch instead, all the models given in the table will be fit. Remark that a general ARIMA model with parameters p for the autoregressive model order, q for the moving-average model order and d representing the number of differencing required to make the time series stationary, is given as follows

$$\alpha(B)\ (1-B)^d\ \tilde{s}(\boldsymbol{t}_i^j)_0 = \beta(B)\ w(\boldsymbol{t}_i^j)_0$$

where B is a lag operator such that $\alpha(B) = 1 - \alpha_1 B - \cdots - \alpha_p B^p$, $\beta(B) = 1 + \beta_1 B + \cdots + \beta_q B^q$ and $w(\boldsymbol{t}_i^j)_0$ is white noise. The fitting procedure aims to extract the Fisher score vector and hence deriving the Fisher kernel.

**ARIMA Model**	**p**	**q**
M^1	0	0
M^2	1	0
M^3	0	1
M^4	1	1
M^5	2	0
M^6	2	1
M^7	0	2
M^8	1	2
M^9	2	2
M^{10}	3	0
M^{11}	3	1
M^{11}	3	2
M^{13}	0	3
M^{14}	1	3
M^{15}	2	3

Table 1. Fitted ARIMA model for every sub-batch $\tilde{s}(t)_0^{i,b}$ with $b = 1, \ldots, 50$. Note that the sub-indices i and j corresponds to number of segments for the healthy and sick patients, respectively, regardless the gender. Hence, for example, for the female case, $i, j = 1, \ldots, N_f$. The parameter d is omitted since it was set equal to 1 for each of the model.

One has $15 \times 50 \times N_f$ fitted models in total for the healthy mini-batches, and the intent is to identify the one that best describes the considered populations of segments, hence the healthy female one. Note that an equivalent procedure will be carried for the healthy male mini-batches. Instead, for the sick mini-batches, one will have $1 \times 50 \times N_f$ fitted models. The same procedure is applied in the male case.

The fitting procedure for the healthy mini-batches is now introduced. Denote the winning model as $M_0^{h_\star,i,j}$, where $h_\star$ is the h-th winning model across the 15 given in Table 1 for each segment $\tilde{s}(\boldsymbol{t}_i^j)_0$. To identify it, consider the Akaike information criterion (AIC). Define AIC for every fitted model on every mini-batch $\tilde{s}(\boldsymbol{t}_i^j)_0$ as follows

$$\mathrm{AIC}_0^{i,j,h} = 2\kappa_0^{i,j,h} - 2\hat{\mathcal{L}}_0^{i,j,h} \; \forall i, \; \forall j$$

where $\kappa_0^{i,j,h}$ is the number of estimated parameters in the model and $\hat{\mathcal{L}}_0^{i,j,h}$ represents the log-likelihood for model h computed for the mini-batch $\tilde{s}(\boldsymbol{t}_i^j)_0$ over the input vector $\boldsymbol{t}_i^j$ defined as

$$\hat{\mathcal{L}}_0^{i,j,h} = \mathcal{L}(\tilde{s}(\boldsymbol{t}_i^j)_0, \boldsymbol{t}_i^j; \hat{\boldsymbol{\theta}}_0) = \sum_{j=1}^{100} \log \ell_{\boldsymbol{t}_i^j}(\tilde{s}(\boldsymbol{t}_i^j)_0, \boldsymbol{t}_i^j; \hat{\boldsymbol{\theta}}_0)$$

Table 2 shows the AICs scores computed from the model fits obtained on all the mini-batches for the healthy female population. The following step is to extract the best model on every mini-batch amongst the 15 fitted models. By referring to Table 2, this means that one model per row will be selected.

**Mini-batch**	$\mathbf{M}^1$	$\mathbf{M}^2$	....	$\mathbf{M}^{15}$
$\tilde{s}(t)_0^{1,1}$	$\text{AIC}_0^{1,1,1}$	$\text{AIC}_0^{1,1,2}$	...	$\text{AIC}_0^{1,1,15}$
$\tilde{s}(t)_0^{1,2}$	$\text{AIC}_0^{1,2,1}$	$\text{AIC}_0^{1,2,2}$	...	$\text{AIC}_0^{1,2,15}$
...	...	...	...	...
$\tilde{s}(t)_0^{1,50}$	$\text{AIC}_0^{1,50,1}$	$\text{AIC}_0^{1,50,1}$	...	$\text{AIC}_0^{1,50,15}$
$s(t)_0^{2,1}$	$\text{AIC}_0^{2,1,1}$	$\text{AIC}_0^{2,1,2}$	...	$\text{AIC}_0^{2,1,15}$
...	...	...	...	...
$\tilde{s}(t)_0^{2,50}$	$\text{AIC}_0^{2,50,1}$	$\text{AIC}_0^{2,50,2}$	...	$\text{AIC}_0^{2,50,15}$
...	...	...	...	...
$\tilde{s}(t)_0^{N_f,1}$	$\text{AIC}_0^{N_f,1,1}$	$\text{AIC}_0^{N_f,1,1}$	...	$\text{AIC}_0^{N_f,1,15}$
...	...	...		... ...
$\tilde{s}(t)_0^{N_f,50}$	$\text{AIC}_0^{N_f,50,1}$	$\text{AIC}_0^{N_m,50,2}$	...	$\text{AIC}_0^{N_m,50,15}$

Table 2. Table summarising all the scorings collected for the mini-batches of the female healthy population of patients, i.e. $\tilde{s}(t)_0$. Note that an equivalent procedure will be applied for the male case.

The best model $M_0^{h_\star}$ will be the one minimising the AIC and hence showing

$$\text{AIC}_0^{h_\star,i,j} = \min_h \text{AIC}_0^{i,j,h} \quad \forall\, i, j$$

where $h = 1, \ldots, 15$. Afterwards, the set of winners models for each $\tilde{s}(\boldsymbol{t}_i^j)_0$ is identified and given as

$$\left\{ M_0^{h_\star,1,1}, \ldots, M_0^{h_\star,1,50}, M_0^{h_\star,2,1}, \ldots, M_0^{h_\star,2,50}, \ldots, M_0^{h_\star,N_f,1}, \ldots, M_0^{h_\star,N_f,50} \right\}$$

The next step consists of selecting N_f winner models, hence one for every segment $\tilde{s}(\boldsymbol{t}_i)_0$ amongst its mini-batches $\tilde{s}(\boldsymbol{t}_i^j)_0$ with $j = 1, \ldots, 50$, and, therefore, the ones that provides

$$\text{AIC}_0^{h_\star,i,j} = \min_j \text{AIC}_0^{h_\star,i,j} \quad \forall\, i$$

where $i = 1, \ldots, N_f$. Hence, N_f winning models are selected fitted over the mini-batches $\tilde{s}(\boldsymbol{t}_i^j)_0$ as

$$\left\{ M_0^{h_\star,1}, M_0^{h_\star,2}, \ldots, M_0^{h_\star,N_f} \right\}$$

Note that, in the above notation, the index of the mini-batches j is dropped since the best model with respect to each segment i is selected. However, the reader should remember that each selected model corresponds to the one fitted over the mini-batches of length 100 samples. Hence, the best model for the segments i was selected amongst the fitted models over the mini-batches $j = 1, \ldots, 50$. In order to construct a weighted Fisher score for the population of healthy female patients proposed in the texting procedure, compute the proportion ρ_0^i reflecting the number of times a model $M_0^{h_\star,i}$ appeared within the set of winning models over the mini-batches as

$$\rho_0^i = \frac{\left| \left\{ M_0^{h_\star,1,1}, M_0^{h_\star,1,2}, \ldots, M_0^{h_\star,1,50}, M_0^{h_\star,2,1}, \ldots, M_0^{h_\star,N_f,50}, \right\} = M_0^{h_\star,i} \right|}{N_f} \quad \forall\, i \qquad (1)$$

Note that $0 \leq \rho_0^i \leq 1$ for $i = 1, \ldots, N_f$ and $\sum_{i=1}^{N_f} \rho_0^i = 1$. Therefore, from this fitting model procedure, a set of N_f winning models and their associated proportion computed as given above will be computed for the female healthy subjects. Remark that the same practice will be applied for the case of the male healthy participants and a set of N_m winning models will be extracted.

For the case of the sick female patients, the procedure goes exactly as the one presented so far. However, the reader should bear in mind that, given the more complexity of the speech signals associated with the presence of Parkison's disease, then only time series ARIMA model fitted to the mini-batches given as $\tilde{s}(\boldsymbol{t}_j^i)_1$ for $j = 1, \ldots, 50$ and $i = 1, \ldots, N_f$ is a (3,1,3) ARIMA model with an intercept. Hence the first step of model selection over the mini-batches will not be required. Furthermore, by following such a procedure, the models for sick and healthy populations will be nested, and the reference model will be the one of the sick patients indeed. In such a way, the GLRT test will provide reliable results given the requirements of nested models.

Note that, the presented procedures consider the observed approximated original signal, i.e. $\tilde{s}(\boldsymbol{t}_j^i)_0$ and $\tilde{s}(\boldsymbol{t}_j^i)_1$ with varying indices i and j depending on the different families. The same procedures will be repeated on the IMFs, and the band-limited IMFs and Fisher score vectors will be equivalently derived. Fig. 4 provides an overview of the fitting procedure proposed for the healthy subjects. It starts with the healthy patient voices on the left, presents the procedure to obtain the segments and then the mini-batches. Afterwards, 15 ARIMA models as given in Table 1 are fitted to each mini-batch. The following step selects the winning model over each mini-batch, and then the model selection stage to then construct the Fisher score vectors is used in the testing procedure. Indeed, the take out of the fitting procedure will be the winning models for each population and their associated proportions.

The best model is chosen over the 50 mini-batches of a segment for every segment of the healthy population (i.e. male or female). Then a collection of Fisher scores defined in the following section will be given. The same procedure applies to the case of sick patients; however, at the stage of the fit, there will be only one model considered.

The next step foresees the description of the testing procedure for the validation model phase which will construct the GLRT test implemented with Fisher vectors for detecting Parkinson's disease. This is presented in the following sections.

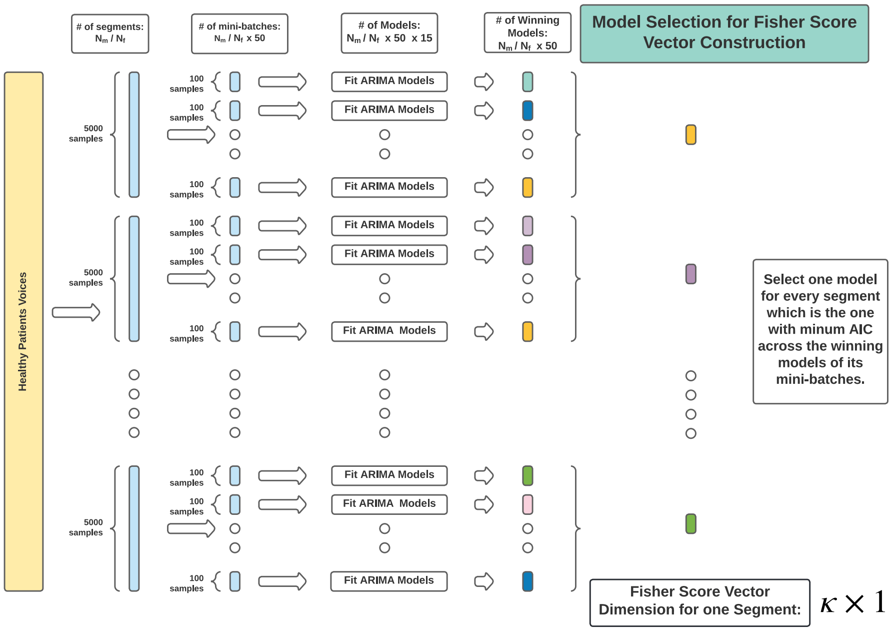

Fig 4. Figure showing a diagram for the steps required for the testing procedure of the model validation phase for the healthy subjects (controls).

5 Testing Procedure for the Model Validation Phase

The testing procedure employed for the model validation phase of analysis is presented in this section. In applying the validation analysis on the testing data, one uses the models obtained from the training phase and evaluate them to the testing data to calculate the test statistic for the GLRT.

In order to perform this evaluation there is a procedure undertaken which is described in the remainder of this section. The objective is to obtain a unique Fisher score computed by aggregating information coming from the set of N_f models for the female case or N_m for the male case which are then subsequently used to conduct a GLRT test with such a derived quantity. This will be done over the test mini-batches for each participant that were not used in the training set of data. Consider the following steps described for the female case as an example, the male case is analogous.

Consider a test segment denoted as $\tilde{s}(\boldsymbol{t}_i)^{\text{ts}}$, where the input variable $\boldsymbol{t}_i$ corresponds to a time index for the i-th segment of the interpolated speech of length 5000 samples. This test segment is then further partitioned into what are termed mini-batches. In this process, each $\tilde{s}(\boldsymbol{t}_i)^{\text{ts}}$ is subsequently split into mini-batches of length 100 sample points (corresponding to 2.2ms) and so this produces for each segment a collection of mini-batches $\tilde{s}(\boldsymbol{t}_i^j)^{\text{ts}}$ with $j = 1, \ldots, 50$ and $i = 1, \ldots, N_{\text{f,test}}$.

Based on the training stage, it will have produced N_f fitted models obtained from the fitting procedure for both poluation samples, i.e. sick and healthy. Each of these models is then evaluated on the constructed testing mini-batches. Note that there is no re-fitting at this stage but just the evaluation of the testing data. Once that is obtained, then the extraction of the Fisher score vectors is required. The procedure for the computation of the Fisher score vector is given as follows.

Consider a test mini-batch denoted as $\tilde{s}(\boldsymbol{t}_i^j)^{\text{ts}}$. For simplicity of the notation and without loss of generality, the index of the segment is dropped since the testing procedure will be conducted at a mini-batch level. Hence, define the set of testing mini-batches as $\tilde{s}(\boldsymbol{t}^j)^{\text{ts}}$. Note that, there will $N_{f,t} = N_{f,\text{test}} \times 50$ mini-batches per participant in the female case. Hence the index j will vary as $j = 1, \ldots, N_{f,t} = 4450$. An equivalent reasoning applies in the male case where one will have $N_{m,t} = N_{m,\text{test}} \times 50$. The index for the extracted model from the fitting procedure will be denoted as $h_\star^0 = 1, \ldots, N_f$ and $h_\star^1 = 1, \ldots, N_f$, for the healthy and sick families, respectively. Once evaluated the log-likelihood on the set of mini-batches of length 100 samples, then the Fisher scores for each model $h_\star^0$ and $h_\star^1$ will be computed for every mini-batch j and will be given as follows:

$$\boldsymbol{U}^j_{\boldsymbol{\theta}_0 \ (100\times\kappa_0^{j,h_\star^0})} = \nabla\boldsymbol{\theta}_0(\mathcal{L}_0^{j,h_\star^0}) \ \forall\, j,\ \forall h_\star^0$$
$$\boldsymbol{U}^j_{\boldsymbol{\theta}_1 \ (100\times\kappa_1^{j,h_\star^1})} = \nabla\boldsymbol{\theta}_0(\mathcal{L}_1^{j,h_\star^1}) \ \forall\, j,\ \forall h_\star^1$$

Since the testing procedure will proceed equally on these two introduced Fisher scores, the following notation is introduced

$$\boldsymbol{U}^j_{\boldsymbol{\theta}_v \ (100\times\kappa_v^{j,h_\star^v})} = \nabla\boldsymbol{\theta}_v(\mathcal{L}_v^{j,h_\star^v}) \ \forall\, j,\ \forall h_\star^v$$

where the index $v = 0, 1$. Note that the index j is in the right-hand side of the above equation since the log-likelihood considered refers to model $h_\star^v$ evaluated on the mini-batch j. Furthermore, the Fisher score is evaluated at each point of the sample, i.e. the mini-batch j, where this score is a matrix $(100 \times \kappa_0^{i,j,h_\star})$, where 100 is the number of samples of the mini-batch and $\kappa_0^{j,h_\star^v}$ is the number of parameters of the model evaluated on that mini-batch given as

$$\kappa_0^{j,h_\star^v} = p + d + 2$$

To construct the Gram matrices required for the GLRT test, firstly the Fisher score is centred as follows:

$$\boldsymbol{U}^{j\ C}_{\boldsymbol{\theta}_v\ \ (100\times\kappa_v^{j,h^v_\star})} = \mathbf{V}^{\intercal}\text{diag}\begin{bmatrix}\hat{\sigma}_{1,1} & .. & .. \\ .. & \hat{\sigma}_{1,1} & .. \\ .. & .. & .. \\ .. & .. & \hat{\sigma}_{\kappa,\kappa}\end{bmatrix}^{-1}\mathbf{V}\ \forall\, j,\ \forall h^v_\star$$

where

$$\mathbf{V} = \left[\boldsymbol{U}^{j}_{\boldsymbol{\theta}_v\ \ (100\times\kappa_v^{j,h^v_\star})}(t) - \hat{\boldsymbol{\mu}}_{\boldsymbol{U}^j_{\boldsymbol{\theta}_v}(t)}\right]$$

$$\hat{\boldsymbol{\mu}}_{\boldsymbol{U}^j_{\boldsymbol{\theta}_v}} = \sum_{t=1}^{100}\boldsymbol{U}^{j}_{\boldsymbol{\theta}_v\ \ (100\times\kappa_0^{j,h^v_\star})}(t,:)\ \ \forall\, j,\ \forall h^v_\star$$

$$\left[\hat{\boldsymbol{\sigma}}_{\boldsymbol{U}^j_{\boldsymbol{\theta}_v}}\right]_s = \frac{\sqrt{\left(\boldsymbol{U}^{i}_{\boldsymbol{\theta}_0\ \ (100\times\kappa_v^{i,h^v_\star})}(t,:)\ -\ \hat{\boldsymbol{\mu}}_{\boldsymbol{U}^j_{\boldsymbol{\theta}_0}}(t,:)\right)^2}}{100}\ \ \forall\, j,\ \forall h^v_\star$$

Note that $\hat{\boldsymbol{\mu}}_{\boldsymbol{U}^j_{\boldsymbol{\theta}_v}}$ represents the sample mean and $\left[\hat{\boldsymbol{\sigma}}_{\boldsymbol{U}^j_{\boldsymbol{\theta}_v}}\right]_s$ represent sample standard deviation estimates of the Fisher score, respectively and are computed over the 100 samples of the mini-batch j linked to its log-likelihood $\mathcal{L}_v^{j,h^v_\star}$, for every MLE estimate. For simplicity, in the notation of the sample mean and sample standard deviation estimates, the dimensionality of the Fisher score is dropped. To avoid ambiguity, within the standard deviation formulation, taken over the columns of the Fisher score, i.e. on the 100 samples for each MLE estimate, and highlight that this calculus is done over the column and not over the entire matrix, $t(:)$ has been introduced. The following step consists of summing up the evaluated Fisher score over the 100 samples for each parameter and hence obtaining

$$\boldsymbol{U}^{j,\ C}_{\boldsymbol{\theta}_v\ \ (1\times\kappa_v^{j,h^v_\star})} = \sum_{t=1}^{100}\boldsymbol{U}^{j\ C}_{\boldsymbol{\theta}_v\ \ (100\times\kappa_v^{j,h^v_\star})}(t)\ \ \forall\, j,\ \forall h^v_\star$$

The left-hand side of the above Fisher score is now of dimension $(1\times\kappa_b^{j,h^v_\star})$ and does not depend on t anymore. This is because the gradients previously evaluated for each parameter at the values $\boldsymbol{t}$ of the given mini-batch $\tilde{s}(\boldsymbol{t}^i_j)^{\text{test}}$ are now summed up together over the vector $\boldsymbol{t}$ and, therefore, a Fisher score vector evaluated at the MLE estimates is now obtained. However, the important step in this construction is that these Fisher scores are centered across the dimension t. Also, the centring indicator has been dropped on the left-hand side, but the reader should bear in mind that these Fisher vectors have been centered for computational stability reasons. Remark now that each mode $h^v_\star$ corresponds to a winning model extracted from the fitting procedure and that the models differ amongst them. They carry the same order in the case of sick patients, i.e. always a (3,1,3) ARIMA model, but in the case of the healthy patients, these models dfferet between them. Each Fisher score vector has, therefore, a different dimension. To construct a unique Fisher score, the obtained Fisher score vectors are modified by padding zeros within the vector up to the number of maximum possible parameters, being $3+3+2=8$. However, the vector will be ordered in terms of the comprised parameter and formally given

$$\boldsymbol{T} = [\delta, \alpha_1, \alpha_2, \alpha_3, \beta_1, \beta_2, \beta_3]$$

where, in order, δ is the intercept of the ARIMA fitted model, $\alpha_1, \alpha_2, \alpha_3$ are the AR parameters and $\beta_1, \beta_2, \beta_3$ the MA paramters. Define now a padding operator given as O

given as

$$\boldsymbol{U}^{j,h^v_\star}_{\boldsymbol{\theta}_v\ (1\times\kappa)} = O\left[\boldsymbol{U}^{j,\,C}_{\boldsymbol{\theta}_v\ (1\times\kappa_v^{j,h^v_\star})}\right]\ \ \forall\ j,\ \forall h^v_\star$$

such that it will return a Fisher vector zero-padded for the elements of $\boldsymbol{T}$ in $\boldsymbol{U}^i_{\boldsymbol{\theta}_v\ (1\times\kappa_v^{j,h^v_\star})}$ that are not present. Hence, this new Fisher vector and will always be of dimension $(1\times\kappa)$ with $\kappa = 8$. Note that, for the healthy category, the intercept position will always be zero by construction. Note that the index for the model $h^v_\star$ is now on the left-hand side. Now, at this point, one will have one Fisher score vector of dimension $(1\times\kappa)$ for every population $v = 0, 1$, every mini-batch $j = 1, \ldots, N_{f,t}$, every model $h^v_\star$. To aggregate the information related to every model evaluated on the testing data and hence capturing structural properties provided by the Fisher vector, for every mini-batch, all the Fisher vectors from every model will be summed up together as

$$\tilde{\boldsymbol{U}}^j_{\boldsymbol{\theta}_v} = \sum_{h^v_\star=1}^{N_f} \rho_v^{h^v_\star}\boldsymbol{U}^{j,h^v_\star}_{\boldsymbol{\theta}_v\ (1\times\kappa)}\ \ \forall j$$

where $\rho_v^{h^v_\star}$ is the proportion computed in Eq. 1 since each Fisher score is weighted according to the proportion of the winning times of that model. Note that in the fitting procedure explanation this was denoted as ρ^i_v and $i = 1, \ldots, N_f$ corresponded to the number of models extracted on a mini-batch which provided the best fit and, therefore, i and $h_\star$ indicates the same quantity. Next, the Gram matrix for the mini-batch $\tilde{s}(\boldsymbol{t}^j_i)_v$ will be defined as

$$\tilde{\mathbf{K}}^j_{v\ (\kappa\times\kappa)} = \tilde{\boldsymbol{U}}^j_{\boldsymbol{\theta}_v}{}^{\intercal}\tilde{\boldsymbol{U}}^j_{\boldsymbol{\theta}_v}\ \ \text{for}\ \ j = 1, \ldots, N_{f,t}$$

To regularise the above matrix due to computational instability that could lead to issues encountered with the inversion of such a matrix or the log-determinant, a covariance shrinkage estimator was considered. The covariance shrinkage estimator of $\tilde{\mathbf{K}}^j_{v\ (\kappa\times\kappa)}$ is given by

$$\tilde{\mathbf{K}}^{j\ S}_{v\ (\kappa\times\kappa)} = (1-\gamma)\,\tilde{\mathbf{K}}^j_{v\ (\kappa\times\kappa)} + \gamma\,\mathbf{Q}\,\mathbb{I}_\kappa\ \kappa$$

where γ is some shrinkage factor, $\mathbb{I}_\kappa$ is the identity matrix of dimension κ and the matrix $\mathbf{Q}$ is given as

$$\mathbf{Q} = \frac{\operatorname{tr}\left[\tilde{\mathbf{K}}^j_{v\ (\kappa\times\kappa)}\right]}{\kappa}$$

Once this is derived, then the GLRT test can be computed for every testing mini-batch j for female case (as for the male ones) as follows:

$$\begin{aligned}\hat{L} = &-(\tilde{\boldsymbol{U}}^j_{\boldsymbol{\theta}_0})\left(\tilde{\mathbf{K}}^{j\ S}_0\right)^{-1}(\tilde{\boldsymbol{U}}^j_{\boldsymbol{\theta}_0})^{\intercal} - \log\left(\det\left[\tilde{\mathbf{K}}^{j\ S}_0\right]\right)\\ &+(\tilde{\boldsymbol{U}}^j_{\boldsymbol{\theta}_1})\left(\tilde{\mathbf{K}}^{j\ S}_1\right)^{-1}(\boldsymbol{U}^j_{\boldsymbol{\theta}_1})^{\intercal} + \log\left(\det\left[\tilde{\mathbf{K}}^{j\ S}_1\right]\right)\end{aligned}$$

In practice, the Generalised Likelihood Ratio Test is evaluated for Fisher score vectors derived from the winning models of the testing set segments with the constructed Gram matrices obtained through the fitting procedure. Fig. **??** in the main body of the paper provides a diagram summarising the steps required for the testing procedure. It is applied to one testing mini-batch and then will be repeated on each of the remaining testing mini-batches. As presented, each model for the two categories of participants will be evaluated on the given mini-batch. Afterwards, according to the steps introduced above, the two Fisher score vectors will be derived by aggregating the individual Fisher scores evaluated with the different model parameters and will provide $\tilde{\boldsymbol{U}}^j_0$ and $\tilde{\boldsymbol{U}}^j_1$. At that point, the Gram Matrices evaluated on that mini-batch can be

computed, and the GLRT test will be then calculated. This process will be repeated for each mini-batch and every patient. Results are provided in the main paper, where the proportion of mini-batches failing to reject the null hypothesis, i.e. being sick, will be shown. The GLRT test will be evaluated for system model one on the approximated signal, while, for the other two system models, the same procedure will be conducted on the first three IMFs.

References

1. Hoehn MM, Yahr MD, et al. Parkinsonism: onset, progression, and mortality. Neurology. 1998;50(2):318–318.
